# Monitoring forest cover and land use change in the Congo Basin under IPCC climate change scenarios

**DOI:** 10.1371/journal.pone.0311816

**Published:** 2024-12-02

**Authors:** Yisa Ginath Yuh, Kouamé Paul N’Goran, Angela Kross, Marco Heurich, H. Damon Matthews, Sarah E. Turner

**Affiliations:** 1 Department of Geography, Planning and Environment, University of Concordia, Montreal, Canada; 2 World Wide Fund for Nature, Regional Office for Africa/Cameroon Country Program Office, Yaoundé, Republic of Cameroon; 3 Faculty of Environment and Natural Resource, University of Freiburg, Freiburg, Germany; 4 Institute for Forest and Wildlife Management, Inland Norway University of Applied Sciences, Koppang, Norway; The Australian National University, AUSTRALIA

## Abstract

The Congo Basin tropical forests are home to many endemic and endangered species, and a global hotspot for forest fragmentation and loss. Yet, little has been done to document the region’s rapid deforestation, assess its effects and consequences, or project future forest cover loss to aid in effective planning. Here we applied the Random Forest (RF) supervised classification algorithm in Google Earth Engine (GEE) to map and quantify decadal changes in forest cover and land use (LCLU) in the Congo Basin between 1990 and 2020. We cross-validated our LCLU maps with existing global land cover products, and projected our validated results to 2050 under three climate change scenarios, using the Multiperceptron Artificial Neural Network and Markov chain algorithms of the Idrissi Land Change modeller from TerrSet. We found that, over 5.2% (215,938 km^2^), 1.2% (50,046 km^2^), and a 2.1% (86,658 km^2^) of dense forest cover were lost in the Congo Basin between 1990–2000, 2000–2010, and 2010–2020, totaling approximately 8.5% (352,642 km^2^) loss estimated between 1990–2020. For the period 2020–2050, we estimated a projected 3.7–4.0% (174,860–204,161 km^2^) loss in dense forest cover under all three climate change scenarios (i.e., 174,860 km^2^ loss projected for SSP1-2.6, 199,608 km^2^ for SSP2-4.5, and 204,161 km^2^ for SSP5-8.5), suggesting that approximately 12.3–12.6% (527,502 km^2^–556,803 km^2^) of dense forest cover could be lost over a 60-year period (1990–2050). Our study represents a novel application of spatial modeling tools and Machine Learning algorithms for assessing long-term deforestation and forest degradation within the Congo Basin, under human population growth and IPCC climate change scenarios. We provide spatial and quantitative results required for supporting long-term deforestation and forest degradation monitoring within Congo Basin countries, especially under the United Nations Framework Convention on Climate Change (UNFCCC) REDD+ (Reduce Emissions from Deforestation and Forest Degradation) program.

## 1. Introduction

Forests cover approximately one third of the Earth’s surface and serve as home to diverse species of plants, animals and fungi, however these ecosystems are threatened due to increased human socioeconomic needs and the impacts of climate change [1, 2]. According to the United Nations Food and Agricultural Organization (FAO), over 420 million hectares (Mha) of forest cover have been lost globally since 1990, with approximately 110 Mha loss between 2010 and 2020 [3]. Africa alone has lost over 3.9 Mha over the last 10 years, a trend expected to continue for the long-term, especially in the Congo Basin where the activities of humans are increasing [4], and the effects of climate-driven vegetation change expected to be exercebated by socioeconomic land use change activities [5].

The tropical forests of the Congo Basin cover approximately 178 Mha [6], and are home to over 10,000 plant, 1000 bird, 400 mammal, 280 reptile, and 700 fish species [7]. However, the Congo Basin is losing over 1Mha of forest cover per year as a result of often externally operated neocolonial economic activities (e.g., industrial logging, small and large-scale clearing for agriculture, and development projects such as the construction of roads and buildings) [2]. Changes in human population density (used as a partial indicator for a combination of anthropogenic drivers, impacts and pressures arising from economic neocolonialism) and human-driven climate change are also contributing factors in forest declines, land use and land cover change (LULCC), and habitat fragmentation and associated loss in habitat connectivity–trends that are expected to continue [2, 8]. However, very little has been done to document the region’s rapid deforestation and forest degradation, to assess its effects and consequences, or to project future forest cover loss to aid in effective planning.

Deforestation and forest degradation are major factors contributing to desertification, biodiversity loss, reduction in ecosystem services, and critically, increased global carbon emmissions [9–13]. For example, between 2001–2019, it has been estimated that globally, over 8.1 ± 2.5 GtCO2 (gigatonnes of carbon dioxide) were emitted to the atmosphere per year as a result of deforestation and forest degradation [12]. Tropical and subtropical regions were estimated to contribute over 78% of these emissions, with primary socioeconomic forest disturbance activities such as small scale clearing for agriculture and logging in forests, contributing between 2–2.4% of all tropical forest carbon emissions [12].

The Congo Basin, which functions as a large tropical carbon sink, and where many large-scale anthropogenic land use activities are supported by local and externally operated neocolonial economic practices and policies [2], is a region of the world where issues with Global carbon emissions seem particularly alarming [12]. With an annual loss of approximately 1Mha of forest reported for this region, and an anticipated fivefold increase in forest loss expected for the future [2], there is an urgent need to monitor and assess long-term deforestation and forest degradation patterns in this region. Such monitoring projects could assist the United Nations Framework Convention on Climate Change (UNFCCC) REDD+ (Reduce Emissions from Deforestation and Forest Degradation) program by providing reliable spatial and quantitative data and maps required for establishing long-term mitigation plans. Moreover, forest cover loss and land use change problems are expected to negatively affect the livelihoods of most rural communities in the tropics (including those in the Congo Basin). Negative impacts include reduced access on customary rights to traditional or community forest managed lands by country governments, which then lead to increased poverty, hunger, and reduced access to non-timber forest medicinal products by forest-dependent communities [11, 14]. In the Congo Basin in particular, almost all forested areas are state-owned, and there already exist several conflicts between national governments and customary land owners [15], suggesting that loss in forest cover could lead to a loss in access to community forests lands, through stricter forest policies. To therefore support the long-term planning of a sustainable forest management system between local communities and national governments, and ensure more social and economic equity for all stakeholders and landrights holders, there is a need to assess large-scale and long-term changes in forest cover and land use patterns in this region. Such studies can provide the baseline information required by various national governments, local communities and supporting conservation organizations for establishing a beneficial sustainable forest management framework for all.

Several studies have attempted to monitor forest cover and land use change in the Congo Basin [2, 16–26]. However, several important variables remain poorly documented, and interactions among socioeconomic and demographic pressures, biophysical disturbances and climate change are often unexamined in land use and land cover change (LULCC) mapping in this region. Climate change, in particular, is expected to impact land use and land cover (LULC) patterns in several regions across the globe (e.g., the Amazon Basin of South America [27]; U.S.A [28]; Europe [29]. Climate change has been projected to interact with other drivers of land use change [e.g., socioeconomic variables such as wood extraction, domestic costs for land, labor and timber, price increase for cash crops, and agricultural and infrastructural expansions; soil erosion; topography; institutional factors related to neocolonial forest policies and poor forest governance; and other human pressures that correlate with population density] to substantially alter land use and land cover change in the long-term [30–32]. To provide accurate assessments of land cover change dynamics within the Congo Basin, for decision making and planning purposes, the effects of climate change need to be considered in present and projected LULCC mapping scenarios.

Most studies in the Congo Basin have mapped and quantified forest cover and land use changes at relatively small geographical scales (e.g., country and landscape scales: [20, 22, 26], or over relatively short time periods (e.g., 1–15 years: [2, 16–20, 22, 24, 26]). However, to the best of our knowledge, no study yet has made comprehensive projections of future forest and land cover conditions that incorporate the predicted effects of socio-economic, demographic, and ecological impacts.

Many datasets on forest cover and land use patterns are freely available for this region. However, these datasets are either limited to short time scales or provide inaccurate data for the Congo Basin e.g., [33, 34]. For example, the Copernicus Global Land Cover dataset and products from the European Land Monitoring Service contain data only for the 2015–2019 period (https://lcviewer.vito.be/download). Collections from the NASA Moderate Resolution Imaging Spectroradiometer (MODIS) Global Forest and Land Cover Scenes contain data only for the years 2010 and 2020, with several limitations identified as a result of inaccuracies with some LULC classes [33]. Datasets generated by the Environmental Systems Research Institute (ESRI), using 10m resolution ESA Sentinel-2 imagery are limited to the year 2020 [34].

Conventional data on forest cover, contributed by various nations, are available through the United Nations Food and Agricultural Organization [3, 35, 36]. However, these datasets are incomplete or lack consistency in geographic coverage and data collection approaches, making harmonization and synthesis difficult [37, 38]. Other global LULC datasets generated by prominent remote sensing researchers exist [39–42]. However, these datasets are either limited to shorter periods (e.g., 2000 or 2020), or to single LULC classes [e.g., global forest cover [39, 40]]; or to global built environments and croplands [41, 42]

Mapping tools and quantitative models can help describe and predict large-scale spatiotemporal changes, for present and future forest cover and land use patterns under corresponding climate change scenarios, as well as various representations in response to demographic and socioeconomic variables. This integrated approach can provide baseline information required for landscape conservation, management and planning for the Congo Basin, and it can inform our overall understanding of global climate change and biodiversity loss.

In this study, therefore, we aimed to: 1) Generate accurate land cover maps for the Congo Basin for the period from 1990 to 2020, and quantitatively assess decadal changes in land cover patterns; 2) Model and project the 1990–2020 land cover maps to the year 2050, under various scenarios of socioeconomic effects, demographic factors, and climate change, to quantify the potential contributions of these aspects to LULCC. We hypothesize that large-scale changes in forest cover will have occurred over the past several decades, in relation to other land use types (e.g., croplands, grasslands, savannas, and built-up areas). We expect further that these changes to forests lands will have been influenced significantly by logging and clearing for agriculture, other human pressures related to increased population density, and climate change. We expect forest cover loss to continue for the year 2050 in response to both anticipated increases inhuman population density, as well as increased global warming.

## 2. Materials and methods

### 2.1. The study area

**Fig 1:**
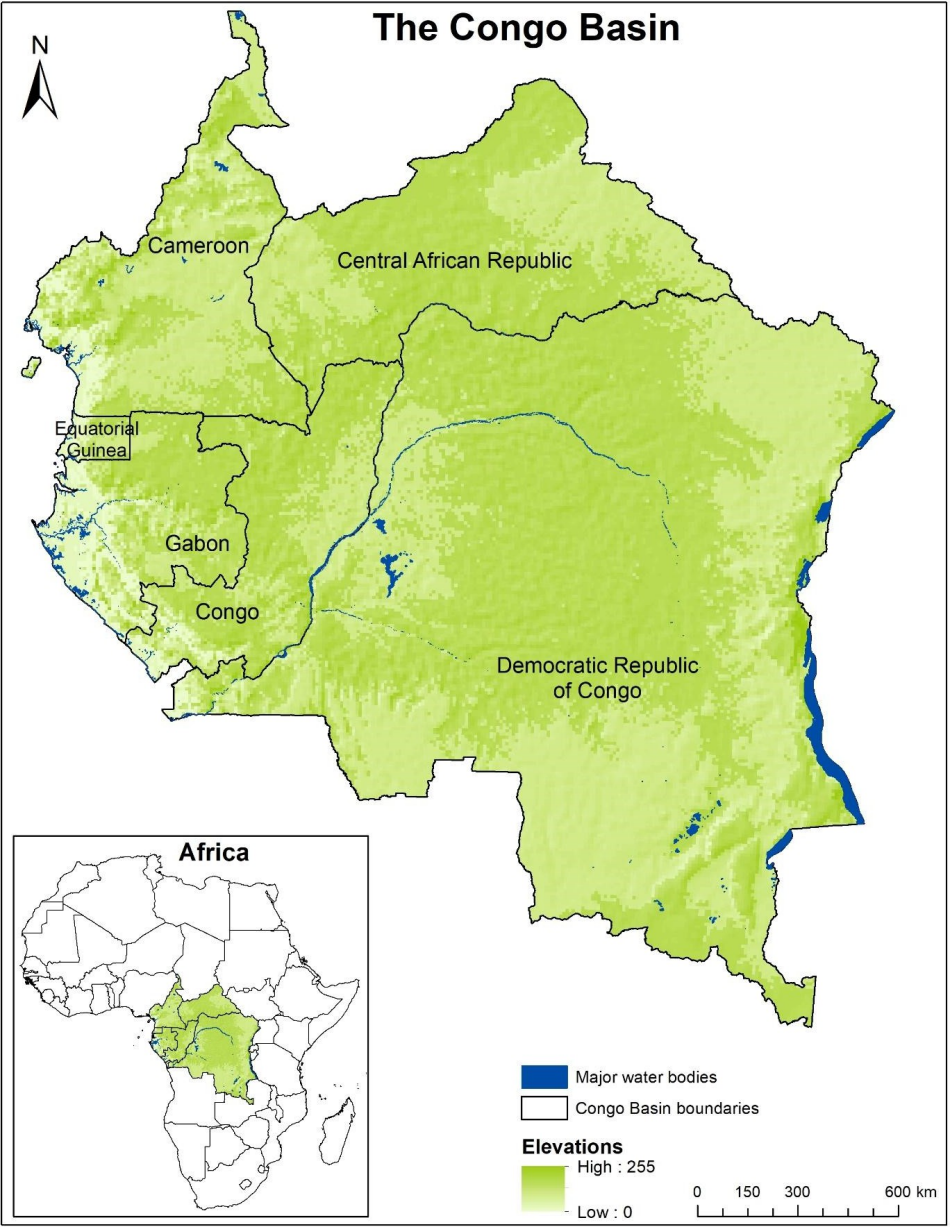
Map of the study area. Map shows the six Central African countries constituting the Congo Basin. Map created by Yisa Ginath Yuh, using open-source spatial layers on administrative boundaries, topography, and water bodies freely available within the WWF Congo Basin geospatial database.

The study area comprises the entire Congo Basin (Fig 1), which lies between latitudes 4°N and 5°S and longitude 12°E, covering six Central African countries: Cameroon, Central African Republic, Gabon, Republic of Congo, Democratic Republic of Congo and Equatorial Guinea. This region covers a total surface area of approximately 4.2 million km^2^ (Table 1), forming the World’s second largest tropical forest (after the Amazon Forest of South America). Prominent flagship faunal species found in this area include central chimpanzees (*Pan troglodytes troglodytes*), western lowland gorillas (*Gorilla gorilla gorilla*), forest elephants (*Loxodanta cyclotis*), bonobos (*Pan paniscus*), and hyena (*Crocuta crocuta*). Prominent floral families include Bambusoideae, Araceae, Araliaceae, Flacourtiaceae, and Marantaceae.

**Table 1:** Congo Basin countries and surface area covered.

Country name	Abbreviation	Total Area (km^2^)
Cameroon	CMR	468,305
Central African Republic	CAR	647,127.5
Congo	RC	345,686.6
Equatorial Guinea	EG	26,982.4
Gabon	GB	265,267.8
Democratic Republic of Congo	DRC	2,498,343.5
**Total Area**		**4,251,712.8**

Annual rainfall ranges from 1500-2000mm and mean annual temperature ranges from 18–28°C across the region [43]. Regional and global climate models, simulated for this region under 21^st^ century warming conditions suggest that mean annual temperatures will rise by approximately 2–4°C by the end of the 21st century, while annual precipitation rates will drop by approximately 6–27% [44, 45]. These changing climatic conditions are expected to have profound effects on vegetation change dynamics in this region, conditions similar to those reported for western and central Sahel regions [46, 47].

The study area is also well known for experiencing high rates of land use activity driven by a variety of socioeconomic pressures [2], most often associated with issues related to economic neocolonialism, poverty, poor governance, and Neo-Malthusianism [48]. Such activities include industrial-scale logging by national and international corporations, small scale clearing for agriculture, large-scale agro-industrial land use, mining, infrastructural development, as well as non-timber forest use for food, medicine, and firewood, and extraction and export of resources to high income countries that foster and perpetuate socioeconomic inequalities [2]. Activities such as industrial logging and small-scale clearing for agriculture contribute to over 45–90% forest disturbance; such perturbations to the forests are also exacerbated by infrastructural development activities that contribute between 4–16% of forest disturbance [2].

Human population, a partial indicator for a combination of anthropogenic drivers of natural system degradation, and impacts and pressures arising from issues associated with economic neocolonialism [48], is also reported to be fast increasing in this region. According the United Nations Population Division, the Congo Basin has experienced an increase in human population of approximately 111 million people between 1990–2020 (i.e., a rise from over 70 million people in the year 1990 to over 181 million people in the year 2020) [49], with over 50–70% of the entire population living in rural areas and in close proximity to forests. Most of these rural people depend on shifting cultivation for subsistence, firewood and charcoal production, and the use of non-timber forest products as food sources and health products [50].

### 2.2. Land use and land cover mapping

To generate LULC maps for our study area for 1990–2020, we used the Google Earth Engine (GEE) cloud computing platform: a platform developed for dealing with large amounts of data. Because our study area covers a total land surface area of approximately 4.2 million km^2^, requiring the processing of over 9 billion raster pixels, we applied the approach used by Midekisa et al. [51] to map LULC. This approach involves using the GEE cloud computing platform to acquire, preprocess, train and classify satellite images using embedded image preprocessing and supervised classification algorithms.

#### 2.2.1. Image acquisition and preprocessing

Using the GEE platform, we acquired Landsat collection 2 surface reflectance Image mosaics from the NASA Earth Observation System, including Landsat 5 and 7 Thematic Mapper images for the years 1990, 2000 and 2010, and Landsat 8 Enhanced Thematic Mapper images for the year 2020 [52–54] (https://www.usgs.gov/landsat-missions/landsat-collection-2-surface-reflectance). We preprocessed the mosaicked images through cloud cover removal with the quality assessment (QA) bands of the acquired Landsat images, using the GEE built-in cloud screening algorithm, and following the approach used by Midekisa et al. [51]. Because cloud removal generally leads to a loss in raster pixels in cloud dominated areas, we acquired Landsat image mosaics for three-year windows for each study period, and applied Midekisa and colleagues’ (2017) approach to calculate the median of all cloud-free pixels for images acquired for each three-year window, to fill areas with missing pixels. For example, for the year 2010, we calculated the median of all cloud-free image pixels acquired between January 1^st^, 2009, and December 31^st^, 2011. This procedure was applied for the years 1990, 2000, and 2020, to generate cloud-free image composites with a complete number of pixels.

Pixel averaging generally affect the reflectance of certain bands within image composites, thereby causing a mismatch in the reflectance of some earth features such as vegetation, water and wetlands, and barren and built-up areas. To therefore, minimize the effects of band reflectance mismatch for the said land cover features, we normalized the image composites for each year of study by computing the median values of the Normalized Differential Vegetation Index (NDVI) and Normalized Differential Water Index (NDWI) for each three-year window [51], and adding them as additional band layers to the Lansat image composites. We further improved the normalization process by adding night-time light images from the National Oceanic and Atmospheric Administration (NOAA) center [51, 55], so as to ease the identification and separation of urban and other built-up areas from open savannas or barelands, which could have similar reflectances in our image band composites. NOAA operates two remote sensing night-time sensors (the Visible Infrared Imaging Radiometer Suite (VIIRS) and the Defense Meteorology Satellite Program (DMSP) Operational Line-scan System (OLS)) that capture visible and Near Infrared (NIR) lights emitted from earth surface features such as built-up areas at night, especially with very little to no cloud cover. For each year of study, we computed the median values of stable night-time light image bands from the NOAA DMSP-OLS sensors for each three-year window and added the Map layers to our normalized images [56, 57].

#### 2.2.2. Image processing

*(a) Sampling training data*. In sampling training datasets, we first identified LULC categories suitable for the study area, following the criteria used in producing the 2010 and 2020 MODIS Global Land Cover Products by the National Aeronautics and Space Administration (NASA) agency [33], as well as the approaches used in generating other single LULC classes, such as the 2000 and 2020 Global Forest Cover Data [39, 41], and the 2000 and 2020 Global Croplands and Built-up data [41, 42]. We built on knowledge from these existing LULC products because they have been generated with high degrees of accuracy, and adequately validated through statistically significant correlations with ancillary datasets from the United Nations Food and Agricultural Organization (FAO), as well as with other global land cover products generated by the NASA Global Ecosystems Dynamics Investigation (GEDI) Service. We therefore used these datasets as references to conduct a balanced and stratified random sampling of pixel points for each suitable LULC class identified for the study area, following sampling approaches applied in Yuh et al. [58]. We identified a total of eight LULC classes (dense forest, cropland, open savanna and bareland, woody savanna, grassland savanna, built-up areas, wetland, and water bodies) (S1 Table), and as such, sampled a total of 5000 pixel points (approximately 625 points for each identified LULC class), constituting the maximum allowable number of points for executing land cover change mapping in Google Earth Engine. When sampling forests (dense forest and woody savanna), for example, we defined forests as areas of land with no primary agricultural or urban land use disturbance, covered with trees ≥ 5m in height [41], and a canopy cover ≥ 20% [33, 59]. Dense forest and woody savanna were separately sampled based on the criteria of Friedl and Sulla-Menashe [33] who defined dense forests as having a > 60% canopy cover, and woody savannas as having a canopy cover of between 30% and 60%. Areas of forests with canopy covers of between 20–29% were removed from our forest sampling because they were highly dominated by agricultural and built areas, and could cause sampling bias under additional criterias defined by the United Nations Food and Agricultural Organization [41]. In sampling cropland, we used the criteria of Friedl and Sulla-Menashe [33] that defined croplands as land use areas with at least 60% of the area cultivated with agricultural crops. In sampling built-up areas, we used the criteria established in Potapov et al. [41], defining built-up areas as human-made landscapes associated with built environments such as commercial and residential infrastructure, urbanized areas, and roads. In sampling water bodies, we followed the criteria used in Friedl and Sulla-Menashe [33], defining water bodies as inland areas covered with at least 60% permanent water, and not obscured by objects above the surface such as buildings, tree canopies, and bridges. In sampling wetlands, we used the criteria of Friedl and Sulla-Menashe [33] that defined wetlands as vegetated and non-vegetated lands inundated with between 30–60% water and usually forming swampy areas or peatlands. For grassland and open savannas, we again applied the criteria of Friedl and Sulla-Menashe [33] that defined grassland savannas as land use areas dominated by herbaceous vegetation or grasses, and open savannas as land use areas that are non-vegetated or contain less than 10% vegetation cover.

*(b) Land use and land cover classification*. To classify LULC for each year of our study, we first divided the sampled training datasets into 80% test and 20% validation subsets. For each test and validation data point, we extracted the Landsat spectral bands (Red, Green, Blue, and Near Infrared bands), NDVI, NDWI, and night-time light layers to be used as covariates in the mapping process. We then modeled and predicted LULC for each year of study by training the test data with the Random Forest decision tree classification algorithm (RF) embedded in the GEE platform. We trained the model using 50 decision trees (DT), allowing the model to randomly select the number of test samples to be trained in each DT by default. RF represents an ensemble of machine learning models that use bootstrap methods to build many single decision tree models [60–62]. The overall model uses a subset of explanatory variables (e.g. Landsat bands) to split observation datasets into a subset of homogenous samples used in building each decision tree [61, 63]. The model has been successfully used in large-scale forest cover mapping [39, 64, 65], wetland mapping [66, 67], cropland mapping [68], and land cover mapping [51, 62, 69].

To validate the LULC classification accuracies for each year of study, we applied two different approaches. In the first approach, classification accuracies (the percentage of accurately classified pixels) were computed and compared with the test and validation datasets using a confusion or error matrix generated with a pivot table in Microsoft Excel (version 2016), following the approach of Yuh et al. [26]. An error matrix is a table used to quantify the classification performance or accuracy of a machine learning algorithm using a set of test and validation datasets. In LULC mapping, the matrix clearly determines the error between two LULC classes i.e., identifies potentially mislabeled land use types. Three commonly used accuracies were computed: overall accuracy, producer’s accuracy (also called the error of omission), and user’s accuracy (also called the error of commission). An overall accuracy (OA) defines the percentage of correctly classified LULC pixels across all classes in a given LULC map [70]. A producer’s accuracy (PA) defines the percentage agreement between the number of pixels correctly classified for a given land cover category and the ground truth observation within a given LULC map [70]. For example, PA for forest = number of correctly classified forest pixels in the classified image / total number of forest pixels in the ground truth dataset (error of omission). A user’s accuracy (UA) defines the percentage agreement between the number of pixels correctly classified for a given land cover category and the total number of pixels classified for the given land cover category in a given land cover map [70]. For example, UA for forest = number of correctly classified forest pixels in the classified image / total number of classified forest pixels in the classified image (error of commission). In the second approach, we applied the method used by Yuh et al. [58]; therefore we employed a Pearson’s correlation test to compute the correlation strength between the generated LULC products in our study and LULC products from already published studies, such as the Hansen et al. [39] Global Forest Cover Data, the 2010 and 2020 MODIS global land cover products [33], and the global built-up land and cropland data published in Potapov et al. [41], and Potapov et al. [42]. The definitions used for our LULC classifications were consistent with those used for our validation datasets, making our correlation tests highly accurate and free from biased estimates. To therefore create the correlation tests, we first, ensured that all the aforementioned land cover products were projected to a similar coordinate reference system of WGS 84, UTM Zone 33N, with the global products further downscaled to a 30m resolution, in order to match the resolutions and rows and column values of our classified land cover products. For each land cover category in each year of available data between the global products and our mapped products, we performed a Boolean operation by reclassifying each land cover category independently with a value of 1 and all other land cover categories with a value of 0 (for example, for dense forests, existing in our land cover maps as well as in the said global land cover products, we reclassified them with a value of 1 and set all other categories to a value of 0). We finally performed a raster stack in R, between the reclassified data for each category between our mapped products and the global available data, and sampled approximately 10000 pixel values on similar XY locations between the global products and our mapped products for the correlation analysis.

#### 2.2.3. Land use and land cover projections

*(a) Acquisition of predictor variables*. To project the 1990–2020 land cover data to the potential land cover in the year 2050, we used three predictor categories that have been proven to be important forest cover loss drivers in the Congo Basin and LULCC drivers in other regions of the world [2, 23–25, 57–59]. These include: land use change driven by socioeconomic factors such as industrial logging, small-scale clearing for agriculture, and large-scale agro-industrial clearing [2]; ecological or climate-driven factors such as landscape topography (slope and elevation), forest fires [71] and climatic variables (temperature and precipitation) [30–32]; and demographic factors, including human population density [72–74], which we used as a partial representation of anthropogenic influence. Although population density itself is not the primary driver of land use change, it is correlated with several aforementioned socioeconomic land use change drivers, as well as other socioeconomic drivers [75], such as those arising from issues with economic neocolonialism (e.g. forest resource exploitation in the region by higher income countries, increase agricultural productivity for export to higher income countries, and socioeconomic inequalities amongst and within nations) [48]. Neocolonialism, an instrument of imperialism, is defined as the exploitation of the less developed parts of the world by powerful and higher income countries in a way that impoverishes (rather than develops) the less developed world, with investments increasing rather than decreasing the gap between the higher and low income countries of the world [76].

Datasets on socioeconomic factors were acquired from a recently published study by Tyukavina et al. [2]. To reduce variable redundancy, we grouped the logging and clearing datasets into a single composite indicator, defined in our model as “logging and forest clearing.” Because developmental areas such as roads, bridges and buildings contribute in facilitating socioeconomic land use activities [77–81], as well as acting as important drivers of land use change [30, 31, 82–84], we acquired datasets on the location and size of built-up areas (e.g. roads and buildings) from mapped datasets by Potapov et al. [41], from which we created an auxiliary socioeconomic factor, “distance to built-up areas.”

Datasets on human population density, consistent with both country-level population and gridded urban fractions, were acquired from the Veiko Lehsten climate and population projection data, archived in the Lund University database (https://dataguru.lu.se/app#worldpop). The datasets consist of human population density projections covering each year between 2010 and 2100, modeled under three shared socioeconomic pathways (SSP1, SSP2 and SSP5), through the Coupled Model Intercomparison Project 6 (CMIP6) of the Intergovernmental Panel on Climate Change (IPCC) [85]. The datasets were mapped at a 1 km spatial resolution, and are consistent with both Shared Socioeconomic Pathways (SSP) and Representative Concentration Pathway (RCP) scenarios of the IPCC framework [85]. We therefore, acquired datasets for the periods between 2010 and 2050, corresponding to our LULC projection periods.

Biophysical factors, including landscape topography (slope and elevation) and the location, size and frequency of forest fires were calculated from digital elevation data acquired from the US geological survey database (http://srtm.usgs.gov/index.php), and from the 2000–2020 MODIS fire database (https://modis.gsfc.nasa.gov/data/dataprod/mod14.php) respectively.

Climatic datasets, including reanalyzed and projected temperature and precipitation (monthly maximum and minimum temperatures, and annual precipitation) datasets were acquired from the IPCC-AR6 ensemble climate projections of the CMIP6 project [86, 87], with future projections data acquired under three Shared Socioeconomic Pathways (SSP1, SSP2, and SSP5), consistent with the low, moderate and high greenhouse gas emission scenarios (RCP 2.6, 4.5, and 8.5) of the IPCC framework (SSP1-2.6, SSP2-4.5 and SSP5-8.5). The SSP1-2.6 climate change scenario represents conditions whereby societies move towards a more sustainable practice in energy and fossil fuel use and global carbon dioxide (CO_2_) emissions are cut to net zero and global temperatures peak during the second half of this century. The SSP2-4.5 represents conditions where CO_2_ emissions start falling as we approach the middle of the 21^st^ century, but do not reach net zero by the end of the 21^st^ century, leading to continued global temperature increases throughout the century. The SSP5-8.5 scenario represents a continued energy-intensive and fossil fuel-based economy, leading to large increases in CO2 emissions and very substantial temperature increases by the end of the century. All datasets were projected at similar coordinate reference systems (WGS 1984, UTM zone 33N), and resampled at similar spatial resolutions (30m resolution) to ease the LULC modeling process. S2 Table provides a full description of these datasets and the available sources.

*(b) The modeling process*. The 1990–2020 land cover maps were projected to 2050 using the Idrisi Land Change Modeler (ILCM) from TerrSet [88, 89], considered under various socioeconomic, demographic, biophysical and climatic change scenarios. The ILCM was developed by Clarks Lab (https://clarklabs.org/terrset/land-change-modeler/) as a stochastic and ensemble model that simulates LULCC between two time steps (T1 and T2), following a set of transition rules assigned to each land cover type, and influenced by anthropogenic and natural disturbance factors [89]. The model uses the Multi-Layer Perceptron Artificial Neural Network (MLP-ANN) [90] that applies a backpropagation modeling approach (i.e. backward transmission of errors from output nodes to input nodes) with a set of explanatory spatial variables to create transition potential maps (i.e., maps of transitions from a single land use class to other land use types) between two time steps (T1 and T2). The transition potential maps were then modeled in an ensemble approach with the Markov chain algorithm to generate LULC maps for a future time step (T3) [91]. The Markov chain algorithm is a stochastic modeling algorithm that, following a set of transition rules, models the probability that a system will remain stable in the future or will change from its previous or current state to a different future state [92].

To project LULCC to the year 2050, we applied the approach of Gibson et al. [91], which involves using the 2010 (T1) and 2020 (T2) LULC maps as baseline maps to: first, create transition potential maps with the MLP-ANN approach, and second, project the transition potential maps to the year 2050 (T3) using the Markov chain algorithm, and under three population change and climate change scenarios (SSP1-2.6, SSP2-4.5 and SSP5-8.5). In creating transition potential maps, we first identified all possible LULCC trajectories between the two current time steps (T1 and T2) (i.e., change from one LULC class in 2010 to another in 2020), following the approach of Yuh et al. [26]. Second, we created transition sub-models that included: grouping LULC transitions or trajectories with respect to the underlying drivers of change [91, 93], quantified using an Ordinary Least Squared Regression (OLS) model (S4–S11 Tables). S3 Table shows the different transition sub-models that were created, including the identified underlying drivers of change, which were selected based on a ≥ 20% influence, following the approach used by Gibson et al. [91]. For each transition model, we evaluated the performance for each LULC transition [i.e., the percentage accuracy for a given LULC type to transition from one land use type to another between two time periods (T1 and T2), under a set of predictor variables]. We used the transition performance results to select the most accurate transitions and predictors to be modeled in an ensemble approach with the Markov chain algorithm of the ILCM.

To ensure accuracy in our future LULC projections, we first used the ILCM and the aforementioned predictors to model our 1990–2000 LULC maps to the years 2010 and 2020, using a Pearson’s correlation test to cross validate the modeled outputs with our 2010 and 2020 LULC maps. Our modeled outputs for the years 2010 and 2020 strongly and significantly correlated with our 2010 and 2020 LULC maps (overall correlation strength: R = 0.8, p < 0.05 for both 2010 and 2020) (S2 Fig and S12 Table), suggesting the reliability of the ILCM in projecting LULCC. We therefore used the 2010 and 2020 LULC maps as baseline maps for projecting LULCC to the year 2050 under three climate and human population change scenarios (SSP1-2.6, SSP2-4.5 and SSP5-8.5). For future modeling, we used similar predictor datasets on logging, fire, and distance to built-up areas acquired under current conditions, as projected future versions of these datasets were not available.

### 2.3. Land use and land cover change detection

We quantified decadal changes (1990–2000, 2000–2010, and 2010–2020) in forest cover and land use patterns, using the ArcGIS geometry tool, following the approaches of Yuh et al. [26] and Yuh et al. [58]. This approach involved: converting our classified LULC maps for each year of study from raster maps to vector shape files; intersecting the datasets on a decadal basis with the ArcGIS intersect tool; creating a sub field “From-TO”; and performing change detection mapping and calculations (in km^2^) from one LULC class to another between two time steps, using spatial statistics with the ArcGIS geometry tool. We further applied a similar approach for quantifying changes between current and projected future conditions (2020–2050) under all three climate change scenarios. We compared these changes at both regional and country scales.

## 3. Results

### 3.1. Model accuracy and LULC mapping and projections

**Fig 2:**
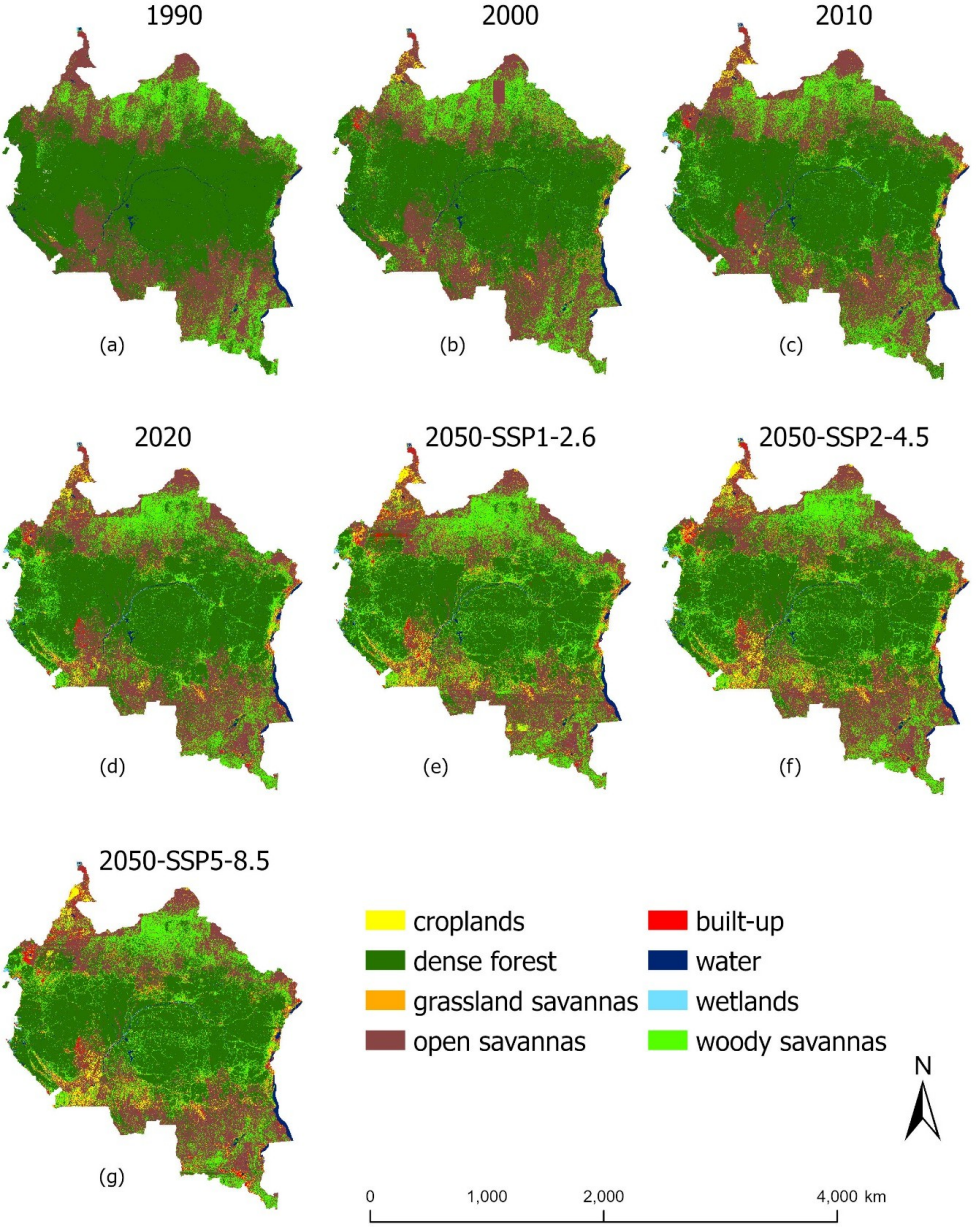
Land cover classification maps for the Congo Basin, for the years 1990, 2000, 2010, 2020, and 2050 (projected under three human population change and climate change scenarios: SSP1-2.6, SSP2-4.5, SSP5-8.5). Map generated with the RF Machine learning model, using the UUSGS Landsat 5 and 7 Collection 2 Level 2 data, freely available for public use with no data restrictions, nor permissions required: https://www.usgs.gov/faqs/are-there-any-restrictions-use-or-redistribution-landsat-data.

The current LULC maps (generated under four time steps: 1990, 2000, 2010 and 2020), as well as projected data for the year 2050 (maps generated under all three population and climate change scenarios: SSP1-2.6, SSP2-4.5 and SSP5-8.5) are shown in Fig 2 (Maps with country boundaries are shown in S1 Fig).

**Fig 3:**
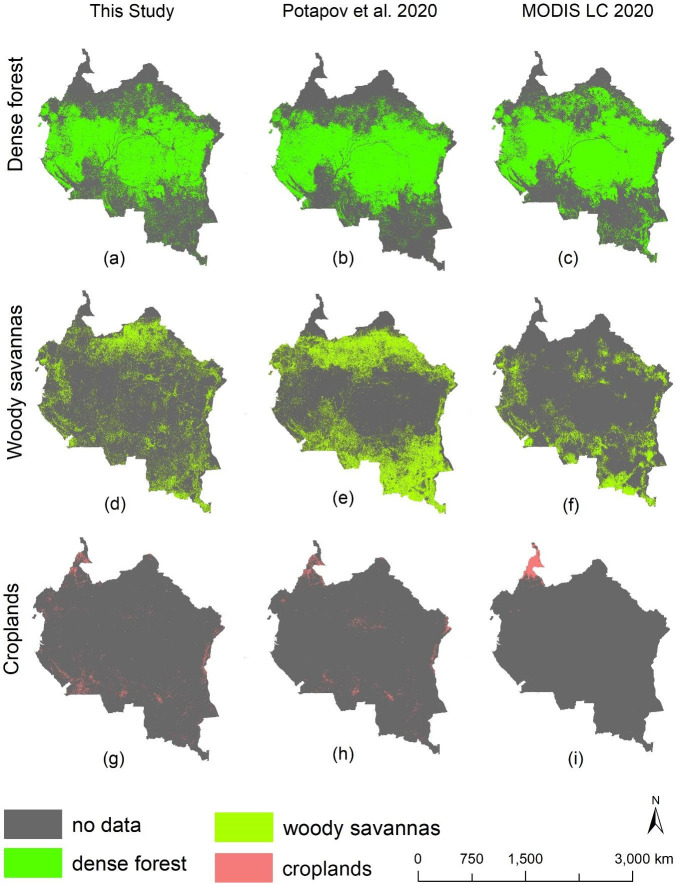
Maps showing comparison between our LULC classes and LULC data extracted from the MODIS global land cover products [33], as well as datasets generated by Potapov et al. **[41], and Potapov et al. [42].** Map comparisons are for the year 2020, and represent a comparison between dense forest (a-c), woody savanna (d-f) and croplands (g-i). Maps generated by Yisa Ginath Yuh, using spatial land cover layers from MODIS, and Potapov et al. [41], and Potapov et al. [42]. MODIS datasets are freely available for public use without any data restrictions, nor permissions required: https://developers.google.com/earth-engine/datasets/catalog/MODIS_061_MCD12Q1#terms-of-use. Spatial land cover layers from Potapov et al. [41], and Potapov et al. [42] are freely available for public use within the global land analysis and discovery database, without any data restrictions or permissions required (https://glad.umd.edu/ard/home).

**Fig 4:**
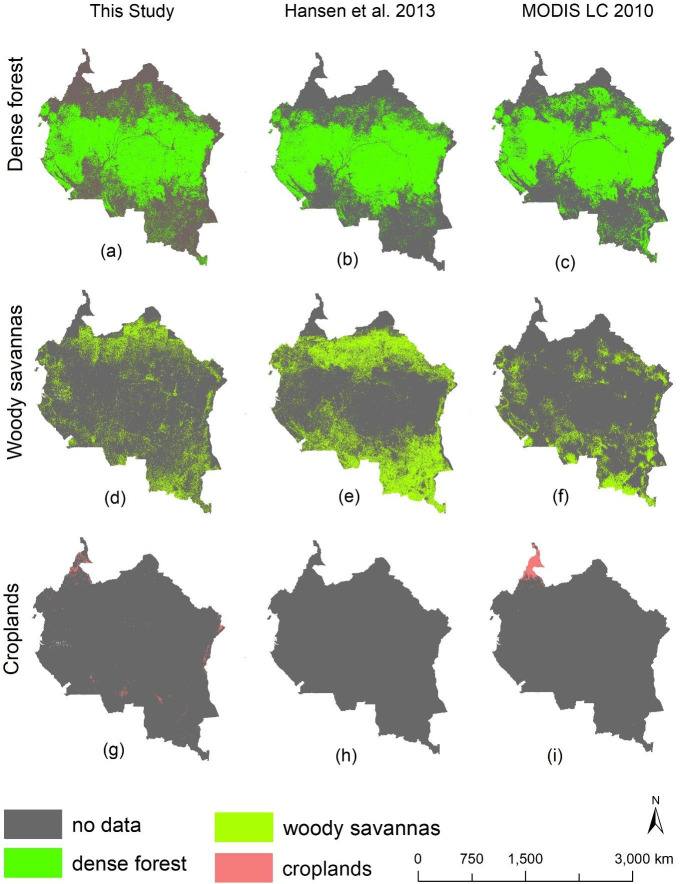
Maps showing comparison between our LULC classes and LULC data extracted from the MODIS global land cover products, [33] as well as products published by Hansen et al. **[39], and Potapov et al. [42].** Map comparisons are for the year 2010, and represent a comparison between dense forest (a-c), woody savanna (d-f) and croplands (g-i). Maps generated by Yisa Ginath Yuh, using spatial land cover layers from MODIS, Hansen et al. [39], Potapov et al. [42]. MODIS datasets are freely available for public use without any data restrictions, nor permissions required: https://developers.google.com/earth-engine/datasets/catalog/MODIS_061_MCD12Q1#terms-of-use. Spatial land cover layers from Potapov et al. [42] are freely available for public use within the global land analysis and discovery web database, and without any data restrictions or permissions required (https://glad.umd.edu/ard/home). The Hansen et al. [39] forest cover datasets are freely available for public use within the University of Maryland open access web database, and without any data restrictions nor permissions required (https://earthenginepartners.appspot.com/science-2013-global-forest/download_v1.5.html).

Our RF model produced an overall classification accuracy of >90% in all four current conditions (Tables 2–5). To further validate these accuracies, we correlated the generated LULC maps with existing land cover products, using a Pearson’s correlation test, and following the approach of Yuh et al. [58], and our results show strong and statistically significant correlations with map products published in Hansen et al. [39], Friedl and Sulla-Menashe [33], Potapov et al. [41], and Potapov et al. [42]. For example, our dense forest areas were strongly and significantly correlated with dense forest extracted from Potapov et al. [41] and Hansen et al. [39] (R = 0.8, p < 0.05 for the year 2020, and R = 0.9, p < 0.05 for the year 2010). Furthermore, we found strong and statistically significant correlations between our croplands and the croplands data published in Potapov et al. [42] (R = 0.9, p < 0.05 for the years 2000 and 2020 respectively). We also found strong and significant correlations between our built-up data and datasets published in Potapov et al. [41] (R = 0.8, p < 0.05 for the years 2000 and 2020 respectively). Correlation results for other LULC classes are shown in Tables 6–8 and Figs 3 and 4.

**Table 2:** Accuracy assessment for the year 1990.

LULC class	Croplands	Dense forest	Grassland savanna	Open savanna/ barelands	Built-up areas	Water bodies	Wetlands	Woody savanna	Overall User’s accuracy
Croplands	85	3	0	24	0	0	0	1	75.2
Dense forest	0	1310	0	19	0	0	0	33	96.2
Grassland savanna	0	17	178	24	0	0	0	18	75.1
Open savanna/barelands	1	22	3	1082	2	0	0	32	94.9
Built-up areas	0	3	0	23	67	0	0	1	71.3
Water bodies	0	1	0	3	0	150	0	0	97.4
Wetlands	0	2	0	13	0	0	57	7	72.2
Woody savanna	0	63	0	63	0	0	0	616	83
**Total**	**86**	**1421**	**181**	**1261**	**69**	**150**	**57**	**708**	**83%**
**Overall producer’s accuracy (%)**	**98.8**	**92.2**	**98.3**	**85.8**	**97.1**	**100**	**100**	**87**	
**Overall accuracy = 94.9%**									

**Table 3:** Accuracy assessment for the year 2000.

LULC class	Croplands	Dense forest	Grassland savanna	Open savanna/ barelands	Built-up areas	Water bodies	Wetlands	Woody savanna	Overall User’s accuracy
Croplands	96	1	0	28	2	0	0	3	76.8
Dense forest	2	1334	1	14	0	0	0	37	96.1
Grassland savanna	1	11	173	36	0	0	0	16	73
Open savanna/barelands	1	20	1	1074	1	1	0	44	94
Built-up areas	3	1	0	21	67	0	0	2	71.3
Water bodies	0	2	0	4	0	154	0	0	96.3
Wetlands	0	1	0	22	0	0	53	3	67.1
Woody savanna	0	69	0	63	0	0	0	610	82.2
**Total**	**103**	**1439**	**175**	**1262**	**70**	**155**	**53**	**715**	**82.1%**
**Overall producer’s accuracy (%)**	**93.2**	**92.7**	**98.9**	**85.1**	**95.7**	**99.4**	**100**	**85.3**	
**Overall accuracy = 93.8%**									

**Table 4:** Accuracy assessment for the year 2010.

LULC class	Croplands	Dense forest	Grassland savanna	Open savanna/ barelands	Built-up areas	Water bodies	Wetlands	Woody savanna	Overall User’s accuracy
Croplands	95	0	0	32	0	0	0	3	73.1
Dense forest	1	1329	1	9	0	0	0	48	95.7
Grassland savanna	0	9	175	33	0	0	0	20	73.3
Open savanna/barelands	1	17	1	1089	2	0	1	31	95.4
Built-up areas	1	0	0	22	68	0	0	3	72.3
Water bodies	0	3	0	4	0	153	0	0	95.6
Wetlands	0	1	0	19	0	0	55	4	69.6
Woody savanna	2	16	3	20	0	0	0	701	94.5
**Total**	**100**	**1375**	**180**	**1228**	**70**	**153**	**56**	**810**	**83.7**
**Overall producer’s accuracy (%)**	**95**	**96.7**	**97.2**	**88.7**	**97.2**	**100**	**98.2**	**86.5**	
**Overall accuracy = 95%**									

**Table 5:** Accuracy assessment for the year 2020.

LULC class	Croplands	Dense forest	Grassland savanna	Open savanna/ barelands	Built-up areas	Water bodies	Wetlands	Woody savanna	Overall User’s accuracy
Croplands	101	1	2	20	3	0	0	3	77.7
Dense forest	0	1345	1	11	1	0	0	30	96.9
Grassland savanna	0	7	179	29	0	0	0	22	75.5
Open savanna/barelands	2	17	1	1090	3	0	0	29	95.4
Built-up areas	2	0	0	6	85	0	1	0	90.4
Water bodies	0	2	0	4	0	154	0	0	96.3
Wetlands	0	1	0	7	0	1	67	3	84.8
Woody savanna	0	23	1	22	0	0	0	696	93.9
**Total**	**105**	**1396**	**184**	**1206**	**92**	**155**	**68**	**783**	**88.9**
**Overall producer’s accuracy (%)**	**96.2**	**96.3**	**97.3**	**90.4**	**92.4**	**99.4**	**98.5**	**88.9**	
**Overall accuracy = 94.9%**									

**Table 6:** Validation of our LULC data for the year 2020. We validate our results through statistically significant correlations with datasets from other published studies. We report correlation strengths only for datasets that are available from the cited studies, and following the approach used in Yuh et al. [58].

Land cover class	Modis global land cover products		Global forest cover data		Global croplands data		Global built-up data	
	Data available?	Correlation strength	Data available?	Correlation strength	Data available?	Correlation strength	Data available?	Correlation strength
Croplands	Yes	0.3	No	NA	Yes	0.9	No	NA
Dense forest	Yes	0.8	Yes	0.8	No	NA	No	NA
Grassland savanna	Yes	0.4	No	NA	No	NA	No	NA
Open savanna/ barelands	Yes	0.5	No	NA	No	NA	No	NA
Built-up areas	Yes	0.3	No	NA	No	NA	Yes	0.8
Water bodies	Yes	0.9	No	NA	No	NA	No	NA
Wetlands	Yes	0.9	No	NA	No	NA	No	NA
Woody savanna	Yes	NS	Yes	NS	No	NA	No	NA
**Overall correlation**		**0.6**		**0.8**		**0.9**		**0.8**

* Modis global land cover products [33]; Global forest cover data [39, 41]; Global croplands data [42]; Global built-up data [41]; NA = Not Applicable; NS = Non-significant

**Table 7:** Validation of our LULC data for the year 2010. We validate our results through statistically significant correlations with datasets from other published studies. We report correlation strengths only for datasets that are available from the cited studies, and following the approach used in Yuh et al. [58].

Land cover class	Modis global land cover products		Global forest cover data		Global croplands data		Global built-up data	
	Data available?	Correlation strength	Data available?	Correlation strength	Data available?	Correlation strength	Data available?	Correlation strength
Croplands	Yes	0.4	No	NA	No	NA	No	NA
Dense forest	Yes	0.9	Yes	0.8	No	NA	No	NA
Grassland savanna	Yes	0.2	No	NA	No	NA	No	NA
Open savanna/ barelands	Yes	0.5	No	NA	No	NA	No	NA
Built-up areas	Yes	0.2	No	NA	No	NA	No	NA
Water bodies	Yes	0.8	No	NA	No	NA	No	NA
Wetlands	Yes	0.7	No	NA	No	NA	No	NA
Woody savanna	Yes	0.7	Yes	0.8	No	NA	No	NA
**Overall correlation**		**0.6**		**0.8**		**NA**		**NA**

* Modis global land cover products [33]; Global forest cover data [39, 41]; Global croplands data [42]; Global built-up data [41]; NA = Not Applicable; NS = Non-significant

**Table 8:** Validation of our LULC data for the year 2000. We validate our results through statistically significant correlations with datasets from other published studies. We report correlation strengths only for datasets that are available from the cited studies, and following the approach used in Yuh et al. [58].

Land cover class	Global forest cover data		Global croplands data		Global built-up data	
	Data available?	Correlation strength	Data available?	Correlation strength	Data available?	Correlation strength
Croplands	No	NA	Yes	0.9	No	NA
Dense forest	Yes	0.9	No	NA	No	NA
Grassland savanna	No	NA	No	NA	No	NA
Open savanna/ barelands	No	NA	No	NA	No	NA
Built-up areas	No	NA	No	NA	Yes	0.8
Water bodies	No	NA	No	NA	No	NA
Wetlands	No	NA	No	NA	No	NA
Woody savanna	Yes	0.7	No	NA	No	NA
**Overall correlation**		**0.8**		**0.9**		**0.8**

* Global forest cover data [39, 41]; Global croplands data [42]; Global built-up data [41]; NA = Not Applicable; NS = Non-significant

Model performances for future projections (i.e., the % accuracy of each LULC category in transitioning from one given land use type to another between two time steps, under the influence of a given change driver) are shown in Table 9. We show performance results only for the most accurate LULC transitions in each transition sub-model.

**Table 9:** Percentage accuracy of each LULC category in transitioning from one land use type to another between two time steps, under the influence of a given change driver.

LULC transition		% transition accuracy/ skill measure	Key drivers
From	To		
Dense forest	Built-up	98	Distance to built-up areas and population density
Dense forest	Croplands	85	Distance to built-up areas, logging and forest clearing, and population density
Dense forest	Grassland savannas	95	Logging and forest clearing, maximum temperatures, wildland fires, and population density
Dense forest	Woody savannas	81	Maximum temperatures, minimum temperatures, and logging and forest clearing
Dense forest	Open savannas/barelands	96	Distance to built-up areas, population density, wildland fires, and logging and forest clearing
Grassland savannas	Dense forest	82	slope
Open savannas/barelands	Dense forest	70	slope
Woody savannas	Dense forest	79	Slope and minimum temperatures
Grassland savannas	Croplands	93	Population density
Woody savannas	Croplands	80	Population density
Croplands to built-up areas	Built-up	94	population density
Grassland savannas	Built-up	88	Distance to built-up areas and population density
Open savannas/barelands	Built-up	93	Distance to built-up areas and population density
Woody savannas	Built-up	96	Distance to built-up areas and population density
Grassland savannas	Woody savannas	71	Precipitation, and slope
Grassland savannas	Water bodies	78	Precipitation and slope
Grassland savannas	Wetlands	70	Precipitation
Open savannas/barelands	Woody savannas	80	Minimum temperature, slope and elevation
Open savannas/barelands	Grassland savannas	76	Precipitation, elevation, and slope
Open savannas/barelands	Water bodies	87	Precipitation and slope
Open savannas/barelands	Wetlands	81	Precipitation, minimum temperatures, and slope
Water bodies	Wetlands	97	Maximum temperatures, and slope
Wetlands	Water bodies	78	Precipitation
Woody savannas	Grassland savannas	82	Population density, logging and forest clearing, and maximum and minimum temperatures

### 3.2. LULC quantification and contributions of predictor variables to LULCC

From our LULC maps, we provide estimates of the total area (in km^2^) occupied by each LULC category in each year of study, as well as quantify detected change areas, i.e., changes from one land use type to another between two time steps, under decadal time scales. Quantified areas and detected changes between 1990–2000, 2000–2010, 2010–2020, and 2020–2050 (under all three climate change scenarios) are shown in Tables 10 and 11 (country-level information: S13–S18 Tables), while transitional changes (i.e., change from one land cover type in time (T1) to another in time (T2)) are shown in S11 Fig and S19 Table.

**Table 10:** Area and proportion of land cover classes in each year of study in the Congo Basin.

	1990		2000		2010		2020		2050					
									SSP1-2.6		SSP2-4.5		SSP5-8.5	
**LULC class**	**Area (km2)**	**% Area**	**Area (km2)**	**% Area**	**Area (km2)**	**% Area**	**Area (km2)**	**% Area**	Area (km2)	% Area	Area (km2)	% Area	Area (km2)	% Area
croplands	1,834.3	0	32,796.8	0.8	35,335.9	0.9	86,181	2.1	182,107	4.4	177,414.7	4.4	187,798.5	4.7
dense forest	2,342,579.7	56.9	2,126,641.4	51.7	2,076,595.9	50.5	1,989,937.8	48.3	1,815,078	44.3	1,790,329	44.3	1,785,777	44.6
grassland/savannas	22,806.6	0.6	50,503.7	1.2	38,129.5	0.9	52,908	1.3	65,855.4	1.6	58,847	1.5	58,891.3	1.5
open savannas/barelands	1,224,361.8	29.8	1,244,488.2	30.3	1,277,365.4	31.0	1,205,256.9	29.3	1,114,673	27.2	1,123,401	27.8	1,110,498	27.7
built-up areas	1,407.9	0	19,740.6	0.5	26,814	0.7	46,548.2	1.1	92,837	2.3	93,346.8	2.3	103,189.2	2.6
water bodies	52,710.2	1.3	62,117.1	1.5	56,215.8	1.4	57,970.2	1.4	56,714.9	1.4	56,793.1	1.4	56,782.4	1.4
wetlands	1,283.4	0	4,923	0.1	9,311.3	0.2	12,388	0.3	11,684.3	0.3	11,717.8	0.3	11,676.5	0.3
woody savannas	468,275.4	11.4	572,513.9	13.9	59,5872.4	14.5	665,461.9	16.2	758,233.6	18.5	732,603.5	18.1	691,153.7	17.3
Total	4,115,259.3	100	4,113,724.7	100	4,115,640.2	100	4,116,652	100	4,097,184	100	4,044,454	100	4,005,766	100

**Table 11:** Quantified decadal changes in land cover patterns in the Congo Basin, between 1990–2020.

	1990–2000		2000–2010		2010–2020		2020–2050					
							SSP1-2.6		SSP2-4.5		SSP5-8.5	
**LULC classes**	**Area (km2)**	**% Area**	**Area (km2)**	**% Area**	**Area (km2)**	**% Area**	**Area (km2)**	**% Area**	**Area (km2)**	**% Area**	**Area (km2)**	**% Area**
croplands	30,962.4	0.8	2,539.2	0.1	50,845.1	1.2	95,926	2.3	91,233.7	2.3	101,617.5	2.6
dense forest	-215,938.3	-5.2	-50,045.5	-1.2	-86,658.2	-2.1	-174,859.6	-4	-199,608.4	-4	-204,161	-3.7
grassland/savannas	27,697.1	0.7	-12,374.2	-0.3	14,778.5	0.4	12,947.4	0.3	5,939	0.2	5,983.3	0.2
open savannas/barelands	20,126.4	0.5	32,877.2	0.8	-72,108.5	-1.8	-90,583.5	-2.1	-81,855.6	-1.5	-94,759.3	-1.6
built-up areas	18,332.7	0.4	7,073.4	0.2	19,734.3	0.5	46,288.8	1.2	46,798.6	1.2	56,641.0	1.5
water bodies	9,406.9	0.2	-5,901.3	-0.1	1,754.3	0	-1,255.3	0	-1,177.1	0	-1,187.8	0
wetlands	3,639.6	0.1	4,388.3	0.1	3,076.7	0.1	-703.7	0	-670.2	0	-711.5	0
woody savannas	104,238.5	2.5	23,358.5	0.6	69,589.5	1.7	92,771.7	2.3	67,141.6	1.9	25,691.8	1.1

We summarize results for four LULC types that predict significant changes to help inform land use planning. They include: Dense forest, woody savannas, built-up areas, and croplands. These LULC categories provide important spatial information that guides our understanding of trends in forest cover dynamics, agricultural land use dynamics, and changes in infrastructural development: information highly important for proper land use planning, and the sustainable management of forest ecosystems and forest resources. For these classes, we present quantified areas and hotspot maps of losses and gains (or expansions) in areas covered by each class for each change period.

#### 3.2.1. Forest cover dynamics

*(a) Dense forest cover*. Our results (Tables 10 and 11, and Fig 7A and 7B) show that dense forest in the Congo Basin declined from 56.9% (2,342,580 km^2^) in the year 1990 to 51.7% (2,126,641 km^2^) in the year 2000, accounting for over 5.2 percentage points (pp) (215,938 km^2^) net loss in the Congo Basin’s dense forest cover over the 10-year period. From 2000–2010, dense forest further declined by 1.2 pp (50,046 km^2^)–i.e., from 51.7% (2,126,641 km^2^) in the year 2000 to 50.5% (2,076,596 km^2^) in 2010. Between 2010 and 2020, a 2.1 pp (86,658 km^2^) loss in dense forest cover was further experienced in the region; dense forest declined from 50.5% (2,076,596 km^2^) of land in the region in 2010 to 48.3% (1,989,938 km^2^) in 2020. For the period 2020–2050, we modeled a 3.7–4 pp (174,860–204,161 km^2^) loss in dense forest cover under all three climate change scenarios. These results generally show that the Congo Basin has a net loss of over 8.6 pp (352,642 km^2^) of its entire dense forest cover over the last 30 years, with an anticipated 12.3 pp (556,803 km^2^) net loss over the full 60-year period of our study (1990–2050). Our findings further show that large areas of dense forest cover loss occurred in areas where dense forests have transitioned either to woody savannas, open savannas/barelands or grassland savannas, or were converted to croplands and built-up areas (Fig 8A, S11 Fig, and S19 Table). Key predictors of dense forest loss in this region include logging and forest clearing (R^2^ = 0.67, p < 0.05), higher maximum and minimum temperatures (R^2^ = 0.66, p < 0.05), wildland fires (R^2^ = 0.42, p < 0.05), population density (R^2^ = 0.21, p < 0.05), and proximity to built-up areas (R^2^ = 0.26, p < 0.05) (S6 Table). In terms of individual land use transitions, forest logging and clearing, human population density, and distance to built-up areas contribute to between 85% and 98% transition accuracy from dense forests to built-up areas and croplands, while wildland fires, maximum and minimum temperatures, and human population density contribute to over 81–95% accuracy in the loss in dense forest cover to grasslands and woody savannas (Table 9).

At the country level, the largest share of dense forest loss is predicted for the DRC, which lost over 140,834 km^2^ of dense forest cover between 1990 and 2000, 4935 km^2^ between 2000 and 2010, and 74,030 km^2^ between 2010 and 2020, with an anticipated loss of 121,832 to 125,382 km^2^ predicted for the period, 2020–2050 (S15 Table). The loss in dense forest cover within the DRC is approximately 7 times greater than losses experienced within other individual Congo Basin countries under all four time scales and under all four change periods (S13–S18 Tables and S3–S8 Figs).

**Fig 5:**
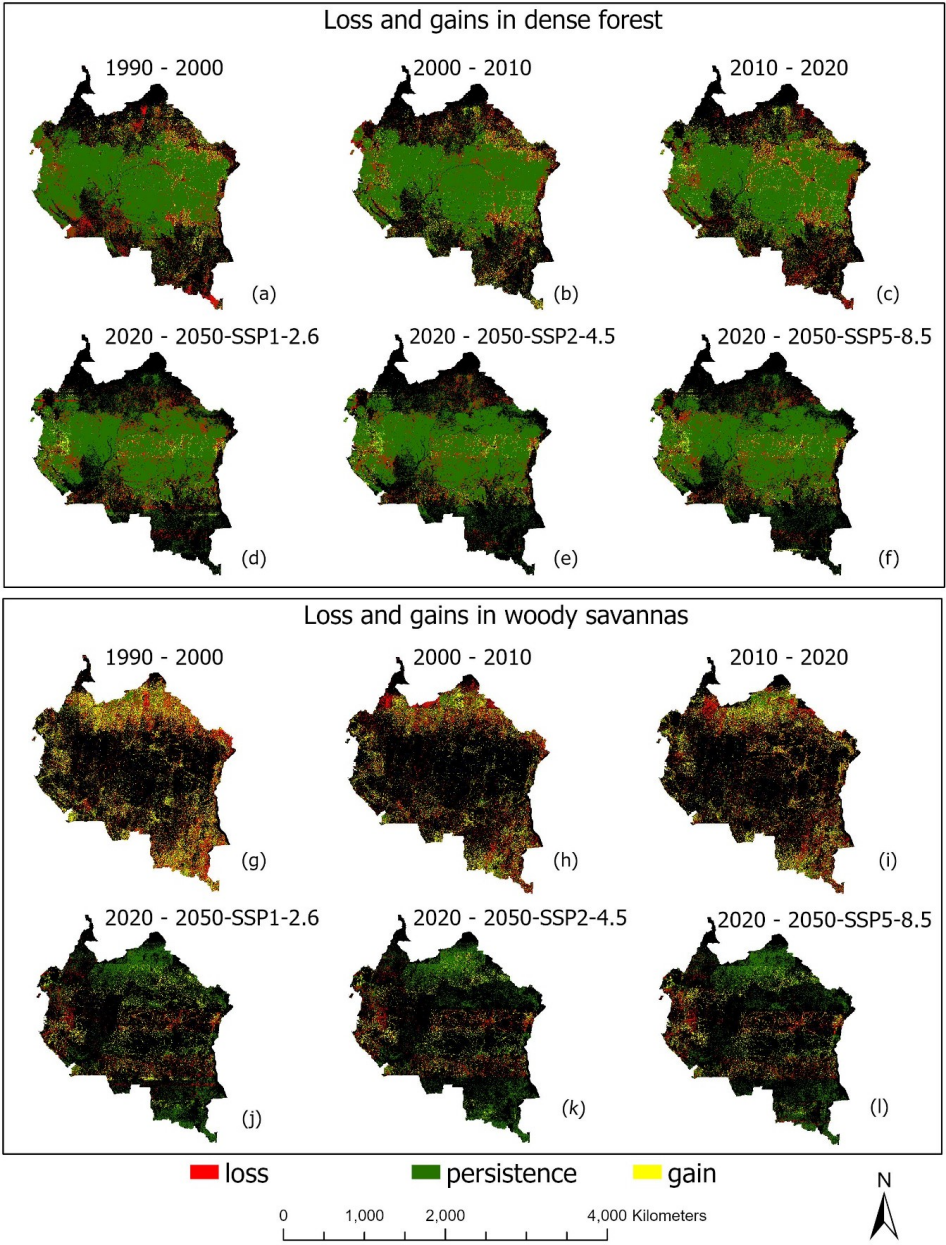
Hotspot maps of losses and gains in dense forests and woody savanna areas within the Congo Basin under six land cover change periods (1990–2000, 2000–2010, 2010–2020, and 2020–2050 under three climate change scenarios: SSP1-2.6, SSP2-4.5, and SSP5-8.5). a-f show loss and gains in dense forest while g-l show loss and gains in woody savannas. Map generated with the RF Machine learning model, using the UUSGS Landsat 5 and 7 Collection 2 Level 2 data, freely available for public use with no data restrictions nor permissions required: https://www.usgs.gov/faqs/are-there-any-restrictions-use-or-redistribution-landsat-data.

Despite the overall loss in dense forest cover, there have also been some areas that have transitioned to dense forest cover from other LULC types, leading to a small gain in dense forest cover in some areas over the last 30 years, with much smaller gains predicted for the future (137,966 km^2^ between 1990 and 2000, 118,584 km^2^ between 2000 and 2010, 216,654 km^2^ between 2010 and 2020, and 95,616 to 103,877 km^2^ from 2020 to 2050). The measured and projected gains are due to changes, actual and predicted, in other LULC types to dense forest cover over the study period (S19 Table). For example, between 1990 and 2000, over 74,176 km^2^, 59,119 km^2^, and 3,018 km^2^ of open savannas/barelands, woody savannas and grassland savannas have been converted to dense forest cover respectively. Between 2000 and 2010, the respective conversions were 79,181 km^2^, 121,462 km^2^, and 10,869 km^2^, while between 2010–2020, they were 70,060 km^2^, 129,135 km^2^, and 6,792 km^2^. We estimate a conversion of approximately 65,746–96,644 km^2^ of woody savannah areas to dense forests by the year 2050 (an area about 2–3 times the size of Belgium (https://statisticstimes.com/geography/countries-by-area.php), and our findings show that the main or key drivers of dense forest cover gain are precipitation and slope parameters, which contribute 69% and 59% influence respectively (Table 9). Fig 8A–8C show comparisons in dense forest cover losses and gains, as well as dense forest transitions within the Congo Basin, while Fig 5A–5F shows hotspot maps of gains and losses (country-level comparisons: S9 and S10 Figs).

*(b) Woody savannas*. Woody savanna areas increased from 11.4% (468,275 km^2^) in 1990 to 13.9% (572,514 km^2^) in the year 2000, accounting for over 2.5 pp (104,239 km^2^) increase in these areas within the Congo Basin (Tables 10 and 11). Between 2000 and 2010, woody savanna areas further increased by 0.6% (23,359 km^2^), i.e., from 13.9% (572,514 km^2^) in the year 2000 to 14.5% (595,872 km^2^) in the year 2010. Finally, between the years 2010 to 2020, we found an additional 1.7 pp (3,077 km2) increase in woody savanna areas ([increased from 14.5% (595,872 km^2^) in 2010 to 16.2% (665,462 km^2^) in 2020], with an anticipated 1–2.3 pp (25,692–92,772 km^2^) gain in woody savanna areas modeled for the year 2050. Overall, there has been an increase in woody savanna areas in the Congo Basin of about 4.8 pp (197,187 km^2^) over the last 30 years, with a 5.9 pp (222,878 km^2^) increase projected over a 60-year period (1990–2050). Key drivers of woody savanna increase in this region include logging and forest clearing (R^2^ = 0.83, p < 0.05), precipitation levels (R^2^ = 0.93, p < 0.05), and slope of the land (R^2^ = 0.31, p < 0.05) (S11 Table). Fig 7G and 7H show trends and changes in woody savanna areas over all four change periods, while Figs 5G–5L and 8J–8L show a comparison in woody savanna gains and losses, quantified transitions, and hotspot maps of gains and losses respectively (country-level comparisons: S9 and S10 Figs).

#### 3.2.2. Changes in built-up areas

Results from Fig 7C and 7D show a continuous expansion in built-up areas under the decadal change periods, with a two-fold expansion expected by the year 2050. Built-up areas increased from 1,408 km^2^ in the year 1990 to 19,741 km^2^ in the year 2000, accounting for over 18,333 km^2^ expansion within the Congo Basin. From 2000 to 2010, built-up areas further expanded by 7,074 km^2^ (from 19,741 km^2^ in the year 2000 to 26,814 km^2^ in 2010). For 2010–2020, a 19,734 km^2^ expansion in built-up areas was further experienced in the region; built-up areas increased from 26,814 km^2^ in the year 2010 to 46,548 km^2^ in the year 2020. For 2020–2050, we project an expansion of built-up areas between 46,289 to 56,641 km^2^ under all three population and climate change scenarios. These results generally show that the Congo Basin has experienced a net expansion in built-up areas of about 45,140 km^2^ over the last 30 years, with an increase of approximately 101,781 km^2^ predicted over a 60-year period (1990–2050).

At the country level, the largest expansion in built-up areas is predicted for the DRC and Cameroon (S15 and S18 Tables). The DRC has experienced an expansion in built-up areas of approximately 7,698 km^2^ between 1990 and 2000; 2,358 km^2^ between 2000 and 2010; and 10,538 km^2^ between 2010 and 2020, with an anticipated 21,300 to 29,548 km^2^ expansion predicted for the period 2020–2050. Cameroon has experienced an expansion in built-up areas of approximately 7,341 km^2^ between 1990 and 2000; 715 km^2^ between 2000 and 2010; and 5,672 km^2^ between 2010 and 2020, with an anticipated 14,395 to 16,330 km^2^ expansion predicted for the period 2020–2050. Key predictors of built-up expansions (conversions from woody savannas, grasslands, and open savannas, as well as dense forests, to built-up areas include human population density (R^2^ = 0.3, p < 0.5), and distance to built-up areas (R^2^ = 0.4, p < 0.5) (S4 Table).

**Fig 6:**
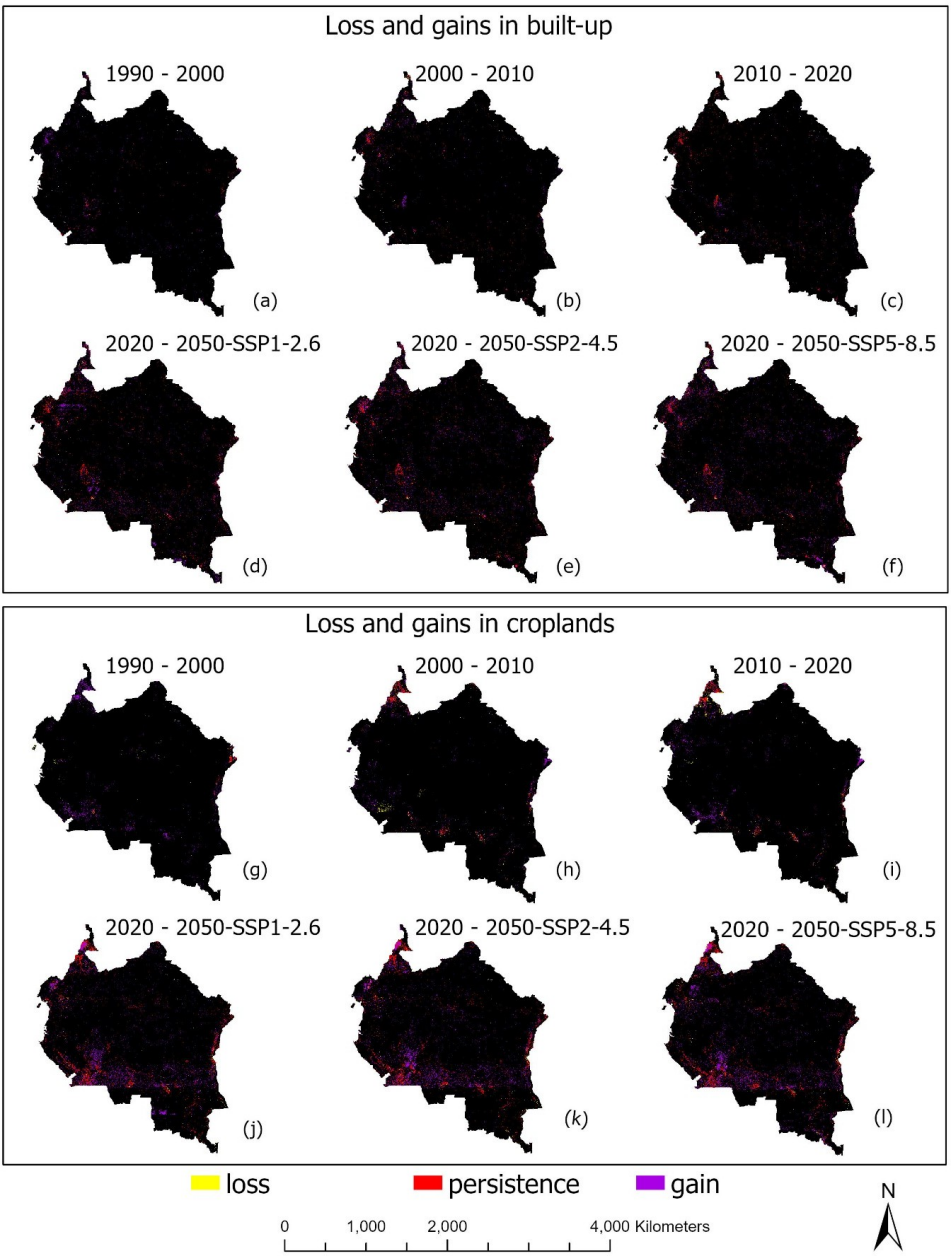
Hotspot maps of losses and gains in built-up and cropland areas within the Congo Basin under six land cover change periods (1990–2000, 2000–2010, 2010–2020, and 2020–2050 under three climate change scenarios: SSP1-2.6, SSP2-4.5, and SSP5-8.5). a-f show loss and gains in built-up areas while g-l show loss and gains in cropland areas. Map generated with the RF Machine learning model, using the UUSGS Landsat 5 and 7 Collection 2 Level 2 data, freely available for public use with no data restrictions nor permissions required: https://www.usgs.gov/faqs/are-there-any-restrictions-use-or-redistribution-landsat-data.

Although the Congo Basin has experienced large expansions in built-up areas over the last 30 years, small proportions of built-up abandonment have also been recorded, with much smaller abandonments predicted for the period up to the year 2050. For example, between the years 1990–2000, approximately 3,007 km^2^ of built-up areas were abandoned and converted to other land use types. Between 2000 and 2010, over 1,645 km^2^ of built-up areas were abandoned, while between 2010 and 2020, over 3,388 km^2^ of built-up areas were abandoned. For the periods 2020–2050, we predict abandoned built-up areas of between 2,703 to 2,890 km^2^ under all three population and climate change scenarios. Fig 8D–8F show comparisons in built-up area gain and loss as well as quantified transitions within the Congo Basin between 1990 and 2050, while Fig 6A–6F show hotspot maps of built-up area expansions and losses predicted under all four change periods (country-level information: S3–S10 Figs). Key predictors of built-up abandonment include slope of the land (R^2^ = 0.7, p < 0.5), and distance to built-up (R^2^ = 0.4, p < 0.5).

#### 3.2.3. Changes in croplands

**Fig 7:**
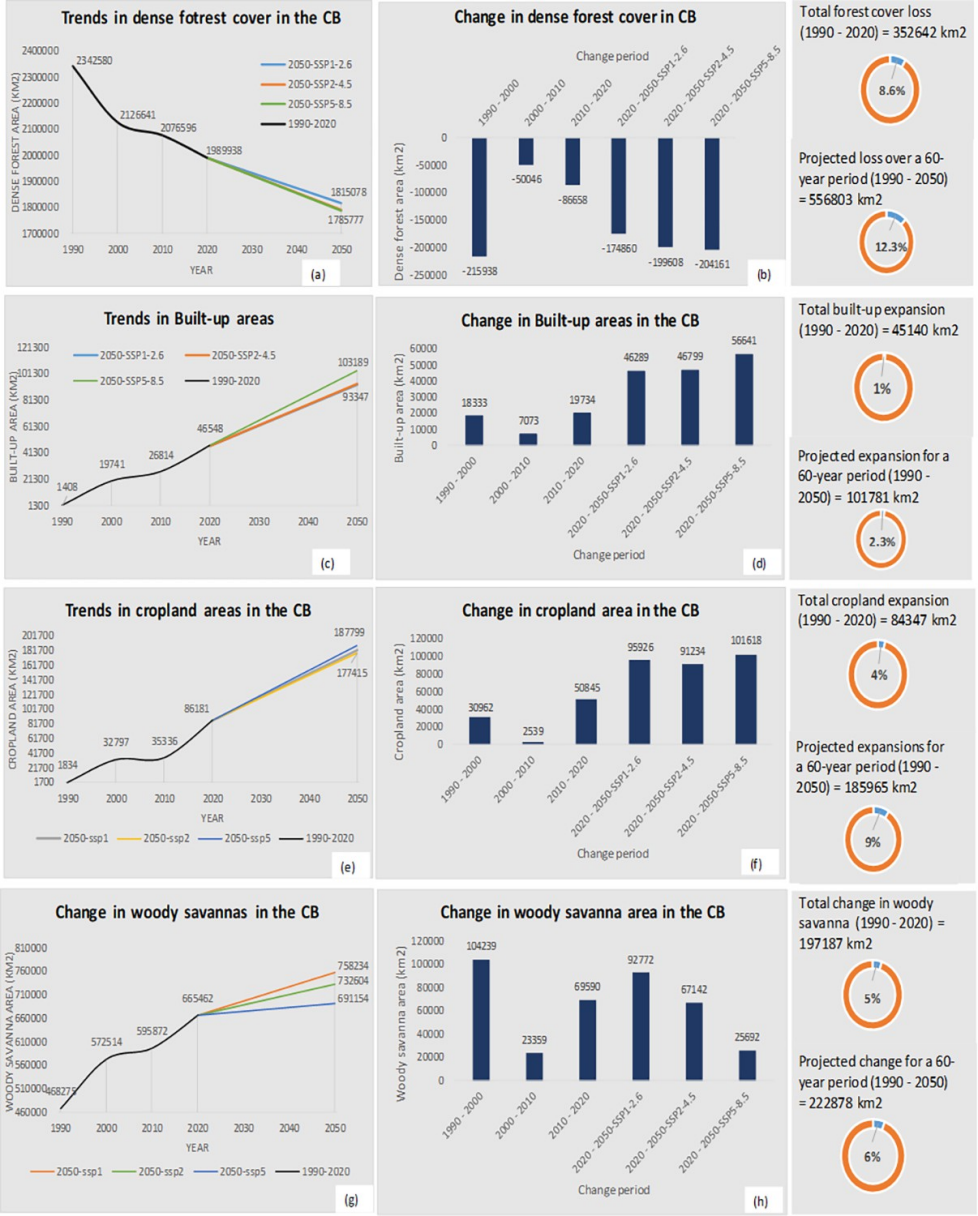
Trends and changes in dense forest cover, built-up, cropland, and woody savanna areas within the Congo Basin between 1990–2020, projected to the year 2050 under three climate change scenarios: SSP1-2.6, SSP2-4.5, and SSP5-8.5. Figs a and b show trends and changes observed in dense forest areas, including information on total and projected forest cover loss for a 60-year period (1990–2050). Figs c and d, e and f, and g and h show similar trend and change information for built-up areas, croplands, and woody savannas respectively. *CB = Congo Basin.

Like built-up areas, our results shown in Tables 10 and 11 and in Fig 7E and 7F also reflect a consistent expansion in cropland areas over time, with a two-fold expansion expected by the year 2050. Cropland areas increased from 1,834 km^2^ in the year 1990 to 32,797 km^2^ in the year 2000, accounting for over 30,962 km^2^ expansion in cropland areas within the Congo Basin. Between 2000 and 2010, cropland areas further expanded by 2,539 km^2^ (from 32,797 km^2^ in the year 2000 to 35,336 km^2^ in 2010), and by the year 2020, a 50,845 km^2^ expansion in cropland areas occurred in the region (from 35,336 km^2^ in 2010 to 86,181 km^2^ in 2020). For the period 2020–2050, we have projected an increase of between 91,234 and 101,618 km^2^ of cropland areas in the region under all three climate change scenarios.

At the country level, the largest expansions in cropland area occurred in the DRC and Cameroon (S15 and S18 Tables). The DRC experienced a cropland area expansion of approximately 14,112 km^2^ between 1990 and 2000. Between 2000 and 2010, over 3,016 km^2^ expansion occurred, while from 2010 to 2020, 27,948 km^2^ expansion occurred. We projected that cropland areas could increase by 47,683 to 54,759 km^2^ in the DRC between the years 2020 and 2050 (S5 Fig). In Cameroon, over 11,812 km^2^ of cropland expansion occurred from 1990 to 2000. Between 2000 and 2010, there was approximately 4,331 km^2^ cropland expansion, while between 2010 and 2020, Cameroon experienced an expansion in cropland areas of about 6,619 km^2^. We projected an increase in cropland areas from 17,804 to 21,277 km^2^ in Cameroon between the years 2020 and 2050 (S8 Fig). We found that the key driver of cropland expansion (i.e. the conversion of woody savannas, grasslands, and open savanna areas to croplands) was increased human population density (R^2^ = 0.2, p < 0.5).

**Fig 8:**
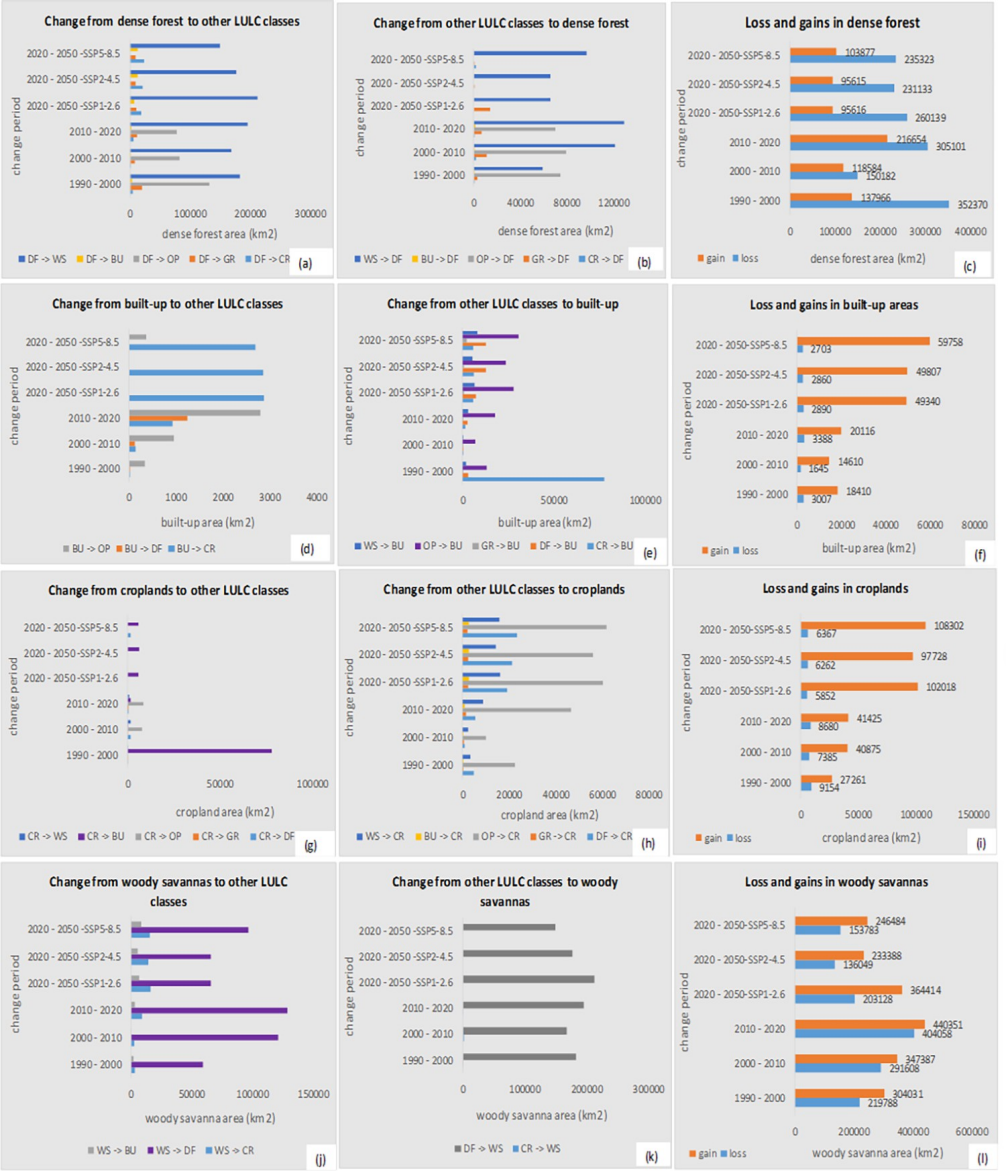
Transitional changes (from-to), as well as loss and gains in dense forest cover, built-up, cropland, and woody savanna areas within the Congo Basin between 1990–2000, 2000–2010, 2010–2020, and 2020–2050 quantified under three climate change scenarios: SSP1-2.6, SSP2-4.5, and SSP5-8.5. Figs a-c show quantified information on transitional changes, as well as loss and gains in dense forest areas, while Figs d-f, g-i, and j-l show change information for built-up, croplands, and woody savanna areas respectively. *WS = woody savannas, BU = built-up, DF = dense forest, CR = croplands, OP = open savannas, GR = grassland savannas.

Though croplands generally expanded over time in our study area, we also observed abandonment of small cropland areas over the last 30 years, with much smaller abandonments predicted for the future. For example, from 1990 to 2000, approximately 9,154 km^2^ of cropland areas were abandoned and converted to other land use types. Between 2000 and 2010, over 7,385 km^2^ of cropland areas were abandoned, while between 2010 and 2020, over 8,680 km^2^ were further lost to other land use types. For 2020 to 2050, we predict an abandonment in cropland areas of from 5,852 to 6,367 km^2^ under all three population and climate change scenarios. Fig 8G–8I show comparisons in cropland area gain and loss as well as quantified transitions within the Congo Basin between 1990 and 2050, while Fig 6G–6L shows a hotspot map of cropland area gains and losses under all four change periods (country level information: S3–S10 Figs). Key drivers of cropland loss include wildland fires (R^2^ = 0.36, p < 0.5), and high maximum temperatures (R^2^ = 0.66, p < 0.5).

## 4. Discussion

We generated accurate LULC maps for the Congo Basin for the last 30 years (1990–2020), and modeled future changes (to 2050) under three climate change scenarios (SSP1-2.6, SSP2-4.5 and SSP5-8.5). We generated these datasets at a fine spatial resolution of 30m, and validated our current LULC maps through statistically significant correlations with existing map products [i.e., [33, 39, 41, 42]]. We mapped and quantified important LULCC trajectories under current and future change conditions, and as well, examined the contributions of socioeconomic, demographic, biophysical and climate change factors to LULCC within this region. We therefore provide novel, reliable and consistent spatial information that deals with the problem of data uncertainty and inconsistencies compiled by the FAO, as reported in Grainger [37] and Matthews [38]. Our datasets provide the baseline information required for landscape planning and management in this region.

Our results revealed dramatic changes in LULC within the Congo Basin, under increased socioeconomic pressures, anthropogenic disturbances and climate change conditions. First, we found that dense forests are declining at rates of approximately 0.3% (11,700 km^2^) per year, with over 8.6% (352,642 km^2^) loss of dense forest areas estimated for the last 30 years. A large proportion of dense forest areas have been converted to built-up areas, croplands, woody savannas, and open savannas (Fig 8A and S19 Table), and our findings show that the main drivers of these conversions are logging and forest clearing, wildland fires, and that land in proximity to built-up areas is more susceptible to changes from these and other human pressures that correlate with population density, as well as higher maximum temperatures (S6 Table). For example, we found that logging and clearing of forests, human population density, and proximity to built-up areas contributed to between 85–98% transition accuracy from dense forests to built-up areas and croplands, while wildland fires, maximum and minimum temperatures, and human population density contribute to over 81–95% accuracy in the loss in dense forest cover to grasslands and woody savannas (Table 9). As human population and land use needs continue to grow and the effects of global warming continue to persist [85, 94], we project a continuous loss in dense forest cover between the years 2020–2050, under all three population and climate change scenarios. Our findings on current losses in dense forest cover corroborate those of Tyukavina et al. [2] and Kirilenko & Sedjo [95] that have shown that the Congo Basin is losing approximately 1Mha (10,000 km^2^) of its forest cover per year as a result of industrial logging, and other types of forest clearing. Although deforestation (which we assume is the loss of dense forest to other LULC types) accounts for over 44% of dense forest cover loss in this region (S19 Table), we found that a large proportion of dense forest areas (approximately 56%) have been degraded through conversion to woody savanna areas (S19 Table); the total forest cover loss in our study area (dense forest + woody savannas) is approximately 5180 km^2^ per year (See Tables 10 and 11). We report overall forest cover loss results that are similar to those generated for other tropical forest regions such as those in Indonesia. Our dense forest cover loss results were also similar to those reported for a neo-tropical forest region, the Brazilian Amazon [81–83]. For example, the Brazilian Amazon Forest experienced a loss in forest cover of approximately 788,300 km^2^ between 1988 and 2018, accounting to over 14,500 km^2^ loss in dense forest cover per year. These results are relatively close to estimates for loss of dense forest cover found in our study [96]. The Indonesian Bornean forest has experienced a total forest cover loss of approximately 168,000 km^2^ between 1973 and 2010, suggesting an annual loss of about 4,500 km^2^ [97]–results that are close to the average annual estimates that we report for all Congo Basin forests (dense forest + woody savannas). These results do not reflect exact time frames as reported in our study, but are however, very close in terms of quantitative estimates and time frames and suggest that loss of dense tropical forest cover is a global issue, not just a regional one.

Forest cover loss (particularly in tropical regions) has been closely linked to anthropogenic land use, associated with socioeconomic pressures [2], arising from increases in human population density [72–74]. These factors interact with many drivers of anthropogenic environmental change, and reflect many factors associated with economic neocolonialism [48]. According to a 2022 report from the United Nations Population Department, the population of Central Africa has increased from approximately 70 million people in the year 1990 to over 181 million people in the year 2020 [49]. Over 50–70% of the entire population in this region lives in rural areas and in close proximity to forests, with the livelihoods of most rural people dependent on shifting cultivation for subsistence, firewood and charcoal production, and the use of non-timber forest products as food sources and health products [50]. A 2016 report by Mosnier [98] has shown that large-scale deforestation in the Congo Basin is attributable to increased agricultural land use, with a case study from the DRC revealing that over 12–27% of forest areas were converted to croplands. While some of this is subsistence farming, much of this conversion arises from the cultivation of high demand cash crops, such as palm oil, corn, cassava, beans, groundnut, sweet potatoes, rice and millet [98], to support rural and urban populations, and international markets that push cash crop production and buy cash crops for export. As with the DRC, we found that Cameroon is also experiencing large-scale expansion of cropland areas, due to increases in local population density and associated need for subsistence, and also as a result of demand for large-scale cash crop exports to neighboring countries such as Gabon, Equatorial Guinea, and the CAR, that depend on Cameroon for many food products [99]. Cameroon is particularly well known for its large-scale conversion of forest areas to oil palm plantations, banana plantations, and cassava, cocoyam, plantain and cocoa farms [100]. For example, oil palm expansion has been reported to contribute to over 67% of the loss in forest cover within Southwest Cameroon [100]. Large-scale expansion of cocoa farms within forest habitats has also been reported for several western African countries, including Cameroon [101]. These findings are in line with our projections for the region.

We also found that the Congo Basin is experiencing large-scale expansions in built-up areas, with a two-fold increase expected by the year 2050. Several infrastructural development projects are ongoing within Congo Basin countries, potentially contributing to the expansion of built-up lands mapped and projected in this study. For example, large-scale road construction (e.g., the $235 million Ketta-Djoum Road project to link the capital cities of Cameroon and the Republic of Congo; the 285-km road linking Ndende in Gabon and Dolisie in the Republic of Congo; and the 500 km project that links Kribi in Cameroon to the Nabeba and iron-ore deposits in the Republic of Congo [102]). Such infrastructural developments contribute to the increase in built-up areas within the Congo Basin by facilitating movement from one urban area to another, and access to formerly remote areas [102]. The development of infrastructure depends on timber; most wood production companies in the Congo Basin carry out industrial logging, with over 7–20% timber products extracted per hectare within timber producing landscapes [103, 104], and our results suggest that such logging practices contribute significantly to forest cover loss in this region. For example, it is reported that over 2–3 million m^3^ of timber are harvested in Cameroon per year [105, 106]. In the DRC, over 3–4 million m^3^ of timber extraction has been reported [107]. If timber exploitation and agricultural land conversions continue to increase, all our projections suggest that the Congo Basin will experience extensive loss in its dense forest cover to croplands and built-up areas by the year 2050 (S19 Table).

We also found significant climatic drivers of LULCC in this region. Limited information is available in the scientific literature on the contributions of climate change to forest cover and land use change in the Congo Basin, although some studies are reporting conversions from grassland savannas to woody savannas in western and central Sahel regions, as a result of increased rainfall and recovery from droughts [46, 47], as well as the exacerbation of climate-driven vegetation change as a result of socioeconomic land use activities [5]. In this study, however, we provided a focused analysis that presents evidence that climate change is a significant and direct contributor to LULC change in this region. We found that higher maximum temperatures contributed significantly to the loss of dense forest cover with associated land cover change to grassland and woody savannas (Table 9 and S14 Table). We also found that higher precipitation contributed significantly to the increase in size and frequency of waterbody and wetlands in our study area (Table 9, and S9 and S10 Tables).

Under future climate change scenarios, we modeled a continued loss in forest cover and expansion of croplands, and concluded that these land cover change patterns were influenced by changing temperature conditions. Climatic changes have been reported to contribute significantly to forest cover and land use change in several regions of the world. For example, in Canada and USA, rising and extremely high temperatures have been shown to correlate with ignition of forest fires, which burn large patches of forested areas yearly, thereby converting them to Tundra forests, grasslands or woody savannas, at least temporarily [108, 109]. Model projections for future scenarios of climate change show shifts in forest cover to other land use types within parts of these regions by the end of the 21^st^ century [110]. Similar scenarios are reported for some tropical regions of the world, such as in the South American Amazon forest [27, 111], and the Indian forests [112, 113]. Our model projections for the Congo Basin align with results reported in these case studies, as well as in studies conducted at global scales [114–116].

The productivity of croplands is also expected to suffer from extreme temperature conditions, where significant proportions of cropland areas are expected to be abandoned and converted to either grasslands, woody savannas, or open savannas, over time. Although we project more expansions than loss in croplands, some studies have shown that climate change will pose serious threats to food production in many regions of the world, with rural Africa expected to be highly vulnerable [94, 117, 118], including the Congo Basin [119, 120]. With the current practices and prospects of Climate-Smart Agriculture (CSA) [121], however, there is evidence that cropland areas will expand in the future in response to the needs of the local human population, but especially driven by the demand from the Global North for export cash crops such as cocoa, tea and palm oil [122]. Our model projects such expansion under all three climate change scenarios. Climate-Smart Agriculture, defined here as climate resilient approaches to agriculture that recognize and integrate challenges associated with food severity and security and climate change is becoming increasingly important for fostering agricultural adaptation in many regions across Africa; suggesting higher prospects for increased crop productivity in the long run (reviewed in). For example, in Ghana, Nigeria, Senegal, and Burkina Faso, the planting of drought-resistant crops as a Climate-Smart Agricultural approach is successful in dealing with climate change impact problems [123–126]. Crop diversification has been implemented as a Climate-Smart Agricultural approach to solving climate impact problems in South Africa [126]. Mixed crop planting, agroforestry, and soil treatments using fertilizers have been applied as Climate-Smart Agricultural approaches in many other African regions [127, 128]. These and more suggest increased potential for future expansions in croplands in many African regions, including the Congo Basin. Although CSA constitutes an important climate resilient approach for fostering increase crop productivity, it should not be used as a major justification for cropland expansion.

### 4.1. Study limitations

Although our study provides novel and accurately validated LULC maps for the last 30 years (1990–2020), as well as projected maps for the future (2050), several limitations exist. First, we validated our land cover maps by correlating them with existing datasets from the MODIS global land cover products [33], as well as with single land use products (global forest cover, croplands, and built-up data) generated by Hansen et al. [39], and Potapov et al. [41], Potapov et al. [42]. Although this approach is robust, better would have been to cross-validate our mapped outputs by correlating them with ground-truthed data or datasets classified at a local level. However, these datasets are unavailable for our study area, considering the large geographic extent. To deal with this limitation, we applied the approach of Yuh et al. [58], that utilized a standardized protocol developed for mapping the MODIS global land cover products, as well as the Hansen et al. [39], and Potapov et al. [41], Potapov et al. [42] global forest cover, croplands and built-up datasets respectively. The Hansen and Potapov datasets have been mapped with high levels of accuracy, and are adequately validated through comparison with more conventional datasets from the United Nations Food and Agricultural Organization (FAO), as well as with other global LULC products generated by the NASA Global Ecosystems Dynamics Investigation (GEDI) service. However, for results validated with the MODIS global land cover products, we apply some caution in interpreting them, as the MODIS global land cover data has several limitations due to the misclassification of some land cover features [33].

Second, it is well known that Landsat clear-sky data is limited for many regions of the world, and particularly with parts of the Congo Basin. Our acquired Landsat products for the years 1990, 2000, 2010, and 2020 had extensive cloud cover in over 40% of the study area, which may have reduced our image radiometric resolutions and pixel numbers during cloud removal. Our method of filling areas of missing raster pixels with Landsat data for three-year windows for each study period has the potential cost of creating an overestimation of some LULC classes, and could account for the minor discrepancies between the total LULC estimated for each study period (Table 10, and S13–S18 Tables). One way of dealing with this problem could have been to use Synthetic Aperture Radar (SAR) datasets, such as those from Sentinel 1; which acquire high quality satellite images under all weather conditions (including both day and night), and at very high spatial resolutions of up to 5m (https://sentinels.copernicus.eu/web/sentinel/missions/sentinel-1/overview). However, we could not use this approach because the temporal coverage of Sentinel 1 does not match our study’s coverage. Though our LULC products produced high degrees of accuracy in all four years of study (Tables 2–5), we again interpret our quantified land cover and change detection results with some caution, owing to the effects of pixels overfitting and class overestimation. We recommend that our approach be tested in future research using SAR images.

Third, in our image processing approach, we used image mosaics that covered the entire study region, but however, had some limitations due to minor artefacts created along scene boundaries. These artefacts created background values in our final maps, that could have possibly contributed to the minor discrepancies observed between the total LULC estimated for each study period.

Fourth, our projected LULC products possess some limitations in the methodological approach. Our classification of wetlands, in many cases incorporates mangrove forests; mangroves are an important forest ecosystem whose deforestation may not be well reflected in our models; in future we hope to address this issue with a project focused on mangrove forest mapping within the Congo Basin at smaller spatial grain sizes, and with the use of high resolution satellite images of < 10m, to minimize sampling bias. Also, datasets on logging and forest clearing, fires and distance to built-up areas are unavailable for the future; in projecting future LULC, we therefore used predictor variables of changing human population density and climatic projections, but held distributions of logging, fire, and distance to built-up areas constant at current values. This could result in future changes attributed to population density changes driven historically by other changing human pressures. Furthermore, population density is not in itself the driver of anthropogenic pressures in the Congo Basin, although population growth in the Global South is often erroneously treated as such [48]. We used population density as a partial indicator of human pressures on the local environment, as it was an available quantitative measure correlated with many socioeconomic drivers of environmental change [75]. However, we recognize that population density at best partially represents the set of anthropogenic factors that cause LULC and other environmental changes.

## 5. Conclusion and management recommendations

Our study represents a novel effort to map and quantify decadal changes in forest cover and land use patterns within the Congo Basin, as well as to model and project specific future LULC changes in the region under IPCC climate change scenarios. We generated accurate LULC maps for the Congo Basin for the years 1990, 2000, 2010, 2020 and projected these LULCC to the year 2050, under three climate change scenarios (SSP1-2.6, SSP2-4.5 and SSP5-8.5). We found that large areas of dense forest cover have been lost to woody savannas, croplands, built-up areas, and open savannas in this region between 1990 and 2020, with continued loss projected for the period 2020–2050 under each of three defined climate change scenarios. Historical loss in dense forest cover in the Congo Basic was highly and significantly influenced by human population density, distance to built-up areas, forest clearing and logging, wildland fires, and high maximum temperatures. In particular, croplands and built-up areas have shown rapid expansion over the historical period, with two-fold expansions projected between 2020–2050. We found that historical expansions were strongly associated with human population density, and distance to built-up areas. Over the last 30 years, human population density has doubled within Congo Basin countries, with more than 50% of the population living in rural areas, and seriously lacking economic and social support systems. Therefore, there is an urgent need to provide economic and social support to these rural areas, enable people in these areas to live sustainably, and support the maintenance of important ecosystem services [129]. Recent research has found that integrating environmental and social objectives can lead to improved forest management outcomes (e.g., Nguyen et al. [130]. Several infrastructural development projects are ongoing in this region, aiming to meet the needs of the future population, and our model projects a two-fold expansion in built-up areas over the next 30 years compared to the historical period, emphasizing the need to integrate environmental, social and economic factors in forest conservation management planning.

For other LULC trajectories (e.g. losses and gains in water bodies and wetlands, as well as losses and gains in woody and grassland savannas), we found that climatic factors, including higher maximum temperatures and greater precipitation are significant contributors to these change trajectories, suggesting the importance of climate change in predicting LULC changes within the Congo Basin. Our results thus fill a critical gap in knowledge about the current state of forests and land use in this region and the potential impacts of human activities, changes in population density and climate change. Results from our analysis are also particularly relevant to initiatives such as the United Nations Framework Convention on Climate Change (UNFCCC) REDD+ (Reduce Emissions from Deforestation and Forest Degradation) program, as they could assist in designing a long-term regional strategy and action plan for monitoring deforestation, and urban expansion within Congo Basin countries. Our current forest cover loss data could be used to estimate the amount of carbon that has been emitted from the Congo Basin through deforestation activities over the last 30 years, while datasets for the year 2050 could serve in predicting future emissions, thereby contributing to the important work of the UNFCCC REDD+ program (https://unfccc.int/files/press/backgrounders/application/pdf/fact_sheet_reducing_emissions_from_deforestation.pdf).

As a means of supporting rural communities in this region with important economic and social support systems that could promote sustainable forest management and help limit negatives impacts from forest cover and land use change, we provide science-informed sustainability-based management recommendations that have been proven successful in other regions of the world [130, 131]. We propose and discuss five key management initiatives to address the negative issues and impacts associated with forest loss, including: 1) the strengthening of community forest management programs and land tenure rights; 2) the promotion of small farming projects or plantations for income generation; 3) the establishment of afforestation or greenspace initiatives to support human health and social wellbeing; 4) increase in access to quality and affordable health care and educational systems, as well as increase rural employment prospects as a means of alleviating poverty; and 5) the promotion of equitable forest benefit sharing between local communities, village elites, and the government, and the payment of Environmental Services (PES) to support such equitable benefit sharing.

Community forest management (CFM) programs and increasing land tenure security have been proven successful strategies in promoting economic and social benefits amongst rural communities, as a means of ensuring sustainable forest management [132]. Secured land tenure ensures local communities will invest in small holder farming or small agroforestry plantations projects that improve household income, while limiting deforestation and forest degradation at large scales [133, 134]. Globally, over 1.5 billion local and Indigenous people (landrights holders [135] have secured forest land tenures [136], however, at national levels, very limited efforts have been employed to establish forest land rights for local communities within middle and low income countries [137, 138]. The Congo Basin is one area of the world where these efforts have been limited, and where there is a high need to support such intitiatives as a means of ensuring socioeconomic benefits and the limiting of large-scale deforestation and forest degradation problem. Therefore, strengthening CFM programs, especially through increase establishments of land tenure rights would go a long way towards promoting sustainable forest management initiatives and ensuring socioeconomic benefits to rural communities in this region, as has been demonstrated in forest governance programs in other tropical countries [139–142], as well as other regions of the world [135, 143]. Moreover, promoting small holder farming or plantation projects through CFM initiatives, as well as creating local markets or important transport systems for the marketing of local food products to larger cities could go a long way in improving rural household income and alleviating poverty in this region. Such initiatives have been proven successful in some parts of Asia [130, 144, 145], and Africa [146–148], however, they remain obstacles to forest protection in the Congo Basin that need to be addressed.

Afforestation or greenspace projects, good and affordable education and healthcare facilities [149], and employment opportunities are other forest management initiatives that could potentially support economic, social, and environmental benefits within rural areas in the Congo Basin, as demonstrated in other regions of the world [149–154]. Increasing forest productivity especially through CFM initiatives could support local communities with important basic needs from forest products such as food, heating and cooking fuel, medicinal products, and shelter, which all promote physical and mental health, and alleviate poverty through income generation [151–153]. Afforestation initiatives also provide more green spaces that serve as important habitats for conserving forest biodiversity. Such initiatives could also provide important carbon sinks, that could help limit global climate change problems in this region. The issuance of forest carbon credits to agricultural and industrial logging companies to help sequester the amount of carbon emitted through industrial logging and large-scale agro-industrial projects could best promote afforestation programs in the Congo Basin, as with other regions of the world [131, 155]. However, there is a need to reduce obstacles to forest conservation, such as reducing carbon transaction costs and improving conservation program design [131, 155, 156]. Such initiatives are still weak in this region, and constitute obstacles to forest protection and climate change mitigation. Several stochastic model projections conducted at a range of geographical scales suggest that a REDD+ framework can be achieved through carbon credit programs [157–159]. There is therefore a need to address these obstacles to better contribute to climate goals, such as the 1.5–2°C warming limit set under the Paris Agreement [160].

The provision of quality education and access to affordable, quality health care services within rural sectors could help alleviate poverty, promote positive feelings towards connected conservation initiatives (e.g., Kirumira et al. [149]), and promote good governance within the forestry sector. Such initiatives can also reduce the need to cut down trees or illegally market timber and non-timber forest products to increase household income, or as a means of meeting up with basic educational and health needs [149, 154]. In addition, engaging rural community inhabitants as contract workers in forest management projects or as eco-guard patrol staff [161] could provide wages to subsidize household incomes and reduce the need for forest resource extraction for income generation or as means of meeting with basic social needs. Conservation initiatives with direct links to social equity and benefits are still rare in the Central African region [162], and their implementation is important for biodiversity conservation.

In addition to CFM and afforestation programs, programs led jointly by government and community can facilitate the PES and equitable forest benefit sharing between local communities, village elites, and the government [163–166]. Joint government-community programs, such as those in other developing regions of the world [163–166], could improve the well-being of disadvantaged local people while promoting sustainable forest policy and management initiatives if implemented in the Congo Basin. Such programs also support the UN REDD+ program, as has already been demonstrated in Cameroon, Indonesia, Peru, Tanzania, Brazil, and Vietnam [167–169].

Our proposed management initiatives are also consistent with many of the UN Sustainable Development Goals, including: Goal 1, No poverty; 2, Zero hunger; 3, Good health and well-being; 4, Quality education; 11, Sustainable cities and communities; and 15, Life on land. These management initiatives could be successfully implemented by the national governments in countries of the Congo Basin, in collaboration with local communities and NGOs. For example, the World Wide Fund for Nature (WWF) that is already conducting an impact monitoring program for the Congo Basin (https://cbmonitoringdashboard-wwf2023.streamlit.app/), the German Agency for International Cooperation (GIZ) is involved in sustainable forest management programs within the Congo Basin (https://www.giz.de/en/worldwide/124747.html), the World Economic Forum (WEF) implements socioeconomic benefit projects to support rural communities in Africa (https://www.weforum.org/communities/africa/), and the International Tropical Timber Organization (ITTO) and World Bank provide financial support towards supporting sustainable forest management and socioeconomic benefit programs to Afro-tropical regions (https://www.itto.int/sfm/2005/details/id=1802; https://www.worldbank.org/en/region/afr).

## Supporting information

S1 Fig Land cover classification maps for Congo Basin countries, for the years 1990, 2000, 2010, 2020, and 2050 (projected under three human population change and climate change scenarios: SSP1-2.6, SSP2-4.5, SSP5-8.5). Map generated with the RF Machine learning model, using the UUSGS Landsat 5 and 7 Collection 2 Level 2 data, freely available for public use with no data restrictions nor permissions required: https://www.usgs.gov/faqs/are-there-any-restrictions-use-or-redistribution-landsat-data.
(TIF)

S2 Fig Comparison between our mapped (original) and predicted LULC products between the years 2010 and 2020. Map products show strong correlations between the original and predicted datasets as quantified in S12A and S12B Table, suggesting the reliability of the ILCM in predicting LULCC. Map generated with the RF Machine learning model, using the UUSGS Landsat 5 and 7 Collection 2 Level 2 data, freely available for public use with no data restrictions nor permissions required: https://www.usgs.gov/faqs/are-there-any-restrictions-use-or-redistribution-landsat-data.
(TIF)

S3 Fig Trends and changes in dense forest cover, built-up, cropland, and woody savanna areas within the Central African Republic (CAR) between 1990–2020, projected to the year 2050 under three climate change scenarios: SSP1-2.6, SSP2-4.5, and SSP5-8.5. Figures a and b show trends and changes observed in dense forest areas, including information on total and projected forest cover loss for a 60-year period (1990–2050). Figures c and d, e and f, and g and h show similar trend and change information for built-up areas, croplands, and woody savannas respectively.
(TIF)

S4 Fig Trends and changes in dense forest cover, built-up, cropland, and woody savanna areas within the Republic of Congo (RC) between 1990–2020, projected to the year 2050 under three climate change scenarios: SSP1-2.6, SSP2-4.5, and SSP5-8.5. Figures a and b show trends and changes observed in dense forest areas, including information on total and projected forest cover loss for a 60-year period (1990–2050). Figures c and d, e and f, and g and h show similar trend and change information for built-up areas, croplands, and woody savannas respectively.
(TIF)

S5 Fig Trends and changes in dense forest cover, built-up, cropland, and woody savanna areas within the Democratic Republic of Congo (DRC) between 1990–2020, projected to the year 2050 under three climate change scenarios: SSP1-2.6, SSP2-4.5, and SSP5-8.5. Figures a and b show trends and changes observed in dense forest areas, including information on total and projected forest cover loss for a 60-year period (1990–2050). Figures c and d, e and f, and g and h show similar trend and change information for built-up areas, croplands, and woody savannas respectively.
(TIF)

S6 Fig Trends and changes in dense forest cover, built-up, cropland, and woody savanna areas within Equatorial Guinea (EG) between 1990–2020, projected to the year 2050 under three climate change scenarios: SSP1-2.6, SSP2-4.5, and SSP5-8.5. Figures a and b show trends and changes observed in dense forest areas, including information on total and projected forest cover loss for a 60-year period (1990–2050). Figures c and d, e and f, and g and h show similar trend and change information for built-up areas, croplands, and woody savannas respectively.
(TIF)

S7 Fig Trends and changes in dense forest cover, built-up, cropland, and woody savanna areas within Gabon between 1990–2020, projected to the year 2050 under three climate change scenarios: SSP1-2.6, SSP2-4.5, and SSP5-8.5. Figures a and b show trends and changes observed in dense forest areas, including information on total and projected forest cover loss for a 60-year period (1990–2050). Figures c and d, e and f, and g and h show similar trend and change information for built-up areas, croplands, and woody savannas respectively.
(TIF)

S8 Fig Trends and changes in dense forest cover, built-up, cropland, and woody savanna areas within Cameroon between 1990–2020, projected to the year 2050 under three climate change scenarios: SSP1-2.6, SSP2-4.5, and SSP5-8.5. Figures a and b show trends and changes observed in dense forest areas, including information on total and projected forest cover loss for a 60-year period (1990–2050). Figures c and d, e and f, and g and h show similar trend and change information for built-up areas, croplands, and woody savannas respectively.
(TIF)

S9 Fig Loss and gains in dense forest cover, built-up, cropland, and woody savanna areas within the Democratic Republic of Congo (DRC), the republic of Congo (RC), and the Central African Republic (CAR) between 1990–2020, projected to the year 2050 under three climate change scenarios: SSP1-2.6, SSP2-4.5, and SSP5-8.5. (TIF)

S10 Fig Loss and gains in dense forest cover, built-up, cropland, and woody savanna areas within Equatorial Guinea (EG), Gabon, and Cameroon between 1990–2020, projected to the year 2050 under three climate change scenarios: SSP1-2.6, SSP2-4.5, and SSP5-8.5. (TIF)

S11 Fig Land cover change detection map for the Congo Basin. Map shows detected changes from one land cover class in time (T1) to another in time (T2). Changes are shown for the most important LULC variables that can help support policy planning. * CR = Croplands; DF = Dense forest; GR = Grassland savannas; OP = Open savannas/barelands; WB = Water bodies; WL = Wetlands; WS = Woody savannas; BU = Built-up; Other LUC = Other Land use and Land cover classes. Map generated with the RF Machine learning model, using the UUSGS Landsat 5 and 7 Collection 2 Level 2 data, freely available for public use with no data restrictions nor permissions required: https://www.usgs.gov/faqs/are-there-any-restrictions-use-or-redistribution-landsat-data.
(TIF)

S1 Table Examples of LULC class descriptions, and their importance in global land cover mapping. (PDF)

S2 Table Sample datasets used as predictors in the land use/cover change projections. (PDF)

S3 Table Transition sub-models to be included in the MLP-ANN of the TerrSet ILCM. Table was designed, following the approach used in Gibson et al. [91], and with drivers of change selected from S4–S11 Tables.
(PDF)

S4 Table Model results showing the influence of each land use change driver on the built-up abandonment and intensification transition sub-models. (PDF)

S5 Table Model results showing the influence of each land use change driver on the croplands abandonment and intensification transition sub-models. (PDF)

S6 Table Model results showing the influence of each land use change driver on the deforestation and afforestation transition sub-models. (PDF)

S7 Table Model results showing the influence of each land use change driver on the grassland savannah area increase and decline transition sub-models. (PDF)

S8 Table Model results showing the influence of each land use change driver on the open savannas/barelands area increase and depletion transition sub-models. (PDF)

S9 Table Model results showing the influence of each land use change driver on water bodies increase and loss transition sub-models. (PDF)

S10 Table Model results showing the influence of each land use change driver on wetlands Increase and loss transition sub-models. (PDF)

S11 Table Model results showing the influence of each land use change driver on woody savannah Area Increase and loss transition sub-models. (PDF)

S12 Table Accuracy validation of predicted LULC data: a; Accuracy validation for our predicted LULC datasets for the year 2010. Table shows Correlation strengths between our mapped LULC data for the year 2010, and datasets predicted for the year 2010 by the TerrSet Idrissi Land Change Modeler. **b**; Accuracy validation for our predicted LULC datasets for the year 2020. Table shows Correlation strengths between our mapped LULC data for the year 2020, and datasets predicted for the year 2020 by the TerrSet Idrissi Land Change Modeler.
(PDF)

S13 Table Quantified area and changes in decadal land cover patterns in CAR: a; Area and proportion of land cover classes in each year of study in CAR. **b**; Quantified decadal changes in land cover patterns in CAR, between 1990–2020.
(PDF)

S14 Table Quantified area and changes in decadal land cover patterns in the Republic of Congo: a; Area and proportion of land cover classes in each year of study in the Republic of Congo. **b**; Quantified decadal changes in land cover patterns in the Republic of Congo, between 1990–2020.
(PDF)

S15 Table Quantified area and changes in decadal land cover patterns in the DRC: a; Area and proportion of land cover classes in each year of study in the DRC. **b**; Quantified decadal changes in land cover patterns in the DRC, between 1990–2020.
(PDF)

S16 Table Quantified area and changes in decadal land cover patterns in EG: a; Area and proportion of land cover classes in each year of study in EG. **b**; Quantified decadal changes in land cover patterns in EG, between 1990–2020.
(PDF)

S17 Table Quantified area and changes in decadal land cover patterns in Gabon: a; Area and proportion of land cover classes in each year of study in Gabon. **b**. Quantified decadal changes in land cover patterns in Gabon, between 1990–2020.
(PDF)

S18 Table Quantified area and changes in decadal land cover patterns in Cameroon: a; Area and proportion of land cover classes in each year of study in Cameroon. **b**; Quantified decadal changes in land cover patterns in Cameroon, between 1990–2020.
(PDF)

S19 Table Quantified areas of LULCC detection. Results are shown for the most important LULC variables that can help support policy planning.
(PDF)

## References

[1] IPCC. Summary for Policymakers. In: Climate Change 2023: Synthesis Report. A Report of the Intergovernmental Panel on Climate Change. Contribution of Working Groups I, II and III to the Sixth Assessment Report of the Intergovernmental Panel on Climate Change [Core Writing Team, H. Lee and J. Romero (eds.)]. IPCC, Geneva, Switzerland, 36 pages. (in press). 2023.

[2] Tyukavina A, Hansen MC, Potapov P, Parker D, Okpa C, Stehman SV, et al. Congo Basin forest loss dominated by increasing smallholder clearing. Sci Adv. 2018;4: eaat2993. doi: 10.1126/sciadv.aat2993 30417092 PMC6221539

[3] FAO. Global Forest Resources Assessment 2020: Main report. Rome: FAO; 2020.

[4] Réjou-Méchain M, Mortier F, Bastin J-F, Cornu G, Barbier N, Bayol N, et al. Unveiling African rainforest composition and vulnerability to global change. Nature. 2021;593: 90–94. doi: 10.1038/s41586-021-03483-6 33883743

[5] Aleman JC, Blarquez O, Gourlet-Fleury S, Bremond L, Favier C. Tree cover in Central Africa: determinants and sensitivity under contrasted scenarios of global change. Sci Rep. 2017;7: 41393. doi: 10.1038/srep41393 28134259 PMC5278362

[6] Mayaux P, Pekel J-F, Desclée B, Donnay F, Lupi A, Achard F, et al. State and evolution of the African rainforests between 1990 and 2010. Phil Trans R Soc B. 2013;368: 20120300. doi: 10.1098/rstb.2012.0300 23878331 PMC3720022

[7] Environment CARP for the. The Forests of the Congo Basin: A Preliminary Assessment. CARPE; 2005. Available: https://books.google.ca/books?id=-pPDNwAACAAJ

[8] Estrada A, Garber PA, Rylands AB, Roos C, Fernandez-Duque E, Di Fiore A, et al. Impending extinction crisis of the world’s primates: Why primates matter. Sci Adv. 2017;3: e1600946. doi: 10.1126/sciadv.1600946 28116351 PMC5242557

[9] Saatchi S, Longo M, Xu L, Yang Y, Abe H, André M, et al. Detecting vulnerability of humid tropical forests to multiple stressors. One Earth. 2021;4: 988–1003. doi: 10.1016/j.oneear.2021.06.002

[10] Saatchi SS, Harris NL, Brown S, Lefsky M, Mitchard ETA, Salas W, et al. Benchmark map of forest carbon stocks in tropical regions across three continents. Proc Natl Acad Sci USA. 2011;108: 9899–9904. doi: 10.1073/pnas.1019576108 21628575 PMC3116381

[11] Intergovernmental Panel On Climate Change (Ipcc). Climate Change 2022 –Impacts, Adaptation and Vulnerability: Working Group II Contribution to the Sixth Assessment Report of the Intergovernmental Panel on Climate Change. 1st ed. Cambridge University Press; 2023. doi: 10.1017/9781009325844

[12] Harris NL, Gibbs DA, Baccini A, Birdsey RA, De Bruin S, Farina M, et al. Global maps of twenty-first century forest carbon fluxes. Nat Clim Chang. 2021;11: 234–240. doi: 10.1038/s41558-020-00976-6

[13] Lawrence D, Coe M, Walker W, Verchot L, Vandecar K. The Unseen Effects of Deforestation: Biophysical Effects on Climate. Front For Glob Change. 2022;5: 756115. doi: 10.3389/ffgc.2022.756115

[14] Hurlbert et al. Risk Management and Decision making in Relation to Sustainable Development. In: Climate Change and Land: an IPCC special report on climate change, desertification, land degradation, sustainable land management, food security, and greenhouse gas fluxes in terrestrial ecosystems [P.R. Shukla, J. Skea, E. Calvo Buendia, V. Masson-Delmotte, H.-O. Pörtner, D.C. Roberts, P. Zhai, R. Slade, S. Connors, R. van Diemen, M. Ferrat, E. Haughey, S. Luz, S. Neogi, M. Pathak, J. Petzold, J. Portugal Pereira, P. Vyas, E. Huntley, K. Kissick, M. Belkacemi, J. Malley, (eds.)]. Risk Management and Decision making in Relation to Sustainable Development. 2019. Available: https://www.ipcc.ch/site/assets/uploads/sites/4/2019/11/10_Chapter-7.pdf

[15] Blaser J, Frizzo J, Norgrove L. Not only Timber: the Potential for Managing Non-timber Forest Products in Tropical Production Forests—a Comprehensive Literature Review. Yokohama, Japan, and Precious Forests Foundation, Zürich, Switzerland: International Tropical Timber Organization (ITTO); 2021. Report No.: Vol. 50.

[16] de Wasseige et al. The forests of the Congo Basin: state of the forest 2008. Luxembourg: Publications Office of the European Union; 2009.

[17] Ernst C, Mayaux P, Verhegghen A, Bodart C, Christophe M, Defourny P. National forest cover change in Congo Basin: deforestation, reforestation, degradation and regeneration for the years 1990, 2000 and 2005. Glob Change Biol. 2013;19: 1173–1187. doi: 10.1111/gcb.12092 23504894

[18] Mayaux P. A land cover map of Africa = Carte de l’occupation du sol de l’Afrique. Luxembourg: Office for Official Publications of the European Communities; 2004.

[19] Mayaux P, Grandi GFD, Rauste Y, Simard M, Saatchi S. Large-scale vegetation maps derived from the combined L-band GRFM and C-band CAMP wide area radar mosaics of Central Africa. International Journal of Remote Sensing. 2002;23: 1261–1282. doi: 10.1080/01431160110092894

[20] Molinario G, Hansen MC, Potapov PV, Tyukavina A, Stehman S, Barker B, et al. Quantification of land cover and land use within the rural complex of the Democratic Republic of Congo. Environ Res Lett. 2017;12: 104001. doi: 10.1088/1748-9326/aa8680

[21] Philippe and Karume. Assessing Forest Cover Change and Deforestation Hot-Spots in the North Kivu Province, DR-Congo Using Remote Sensing and GIS. 2019;8: 39–54.

[22] Potapov PV, Turubanova SA, Hansen MC, Adusei B, Broich M, Altstatt A, et al. Quantifying forest cover loss in Democratic Republic of the Congo, 2000–2010, with Landsat ETM+ data. Remote Sensing of Environment. 2012;122: 106–116. doi: 10.1016/j.rse.2011.08.027

[23] Verhegghen A. Review and Combination of Recent Remote Sensing Based Products for Forest Cover Change. International Forestry Review. 2015;18: 14–25.

[24] Verhegghen A, Mayaux P, de Wasseige C, Defourny P. Mapping Congo Basin vegetation types from 300 m and 1 km multi-sensor time series for carbon stocks and forest areas estimation. Biogeosciences. 2012;9: 5061–5079. doi: 10.5194/bg-9-5061-2012

[25] Ygorra B, Frappart F, Wigneron JP, Moisy C, Catry T, Baup F, et al. Monitoring loss of tropical forest cover from Sentinel-1 time-series: A CuSum-based approach. International Journal of Applied Earth Observation and Geoinformation. 2021;103: 102532. doi: 10.1016/j.jag.2021.102532

[26] Yuh YG, Dongmo ZN, N’Goran PK, Ekodeck H, Mengamenya A, Kuehl H, et al. Effects of Land cover change on Great Apes distribution at the Lobéké National Park and its surrounding Forest Management Units, South-East Cameroon. A 13 year time series analysis. Sci Rep. 2019;9: 1445. doi: 10.1038/s41598-018-36225-2 30723223 PMC6363750

[27] López L, Villalba R, Stahle D. High-fidelity representation of climate variations by Amburana cearensis tree-ring chronologies across a tropical forest transition in South America. Dendrochronologia. 2022;72: 125932. doi: 10.1016/j.dendro.2022.125932

[28] Mu JE, Sleeter BM, Abatzoglou JT, Antle JM. Climate impacts on agricultural land use in the USA: the role of socio-economic scenarios. Climatic Change. 2017;144: 329–345. doi: 10.1007/s10584-017-2033-x

[29] Carozzi M, Martin R, Klumpp K, Massad RS. Effects of climate change in European croplands and grasslands: productivity, greenhouse gas balance and soil carbon storage. Biogeosciences. 2022;19: 3021–3050. doi: 10.5194/bg-19-3021-2022

[30] Geist HJ, Lambin EF. Dynamic Causal Patterns of Desertification. BioScience. 2004;54: 817. doi: 10.1641/0006-3568(2004)054[0817:DCPOD]2.0.CO;2

[31] Geist HJ, Lambin EF. Proximate Causes and Underlying Driving Forces of Tropical Deforestation. BioScience. 2002;52: 143. doi: 10.1641/0006-3568(2002)052[0143:PCAUDF]2.0.CO;2

[32] Hellwig N, Walz A, Markovic D. Climatic and socioeconomic effects on land cover changes across Europe: Does protected area designation matter? Joseph S, editor. PLoS ONE. 2019;14: e0219374. doi: 10.1371/journal.pone.0219374 31314769 PMC6636817

[33] Friedl Mark, Sulla-Menashe Damien. MCD12Q1 MODIS/Terra+Aqua Land Cover Type Yearly L3 Global 500m SIN Grid V006. NASA EOSDIS Land Processes DAAC; 2019. doi: 10.5067/MODIS/MCD12Q1.006

[34] Karra K, Kontgis C, Statman-Weil Z, Mazzariello JC, Mathis M, Brumby SP. Global land use / land cover with Sentinel 2 and deep learning. 2021 IEEE International Geoscience and Remote Sensing Symposium IGARSS. Brussels, Belgium: IEEE; 2021. pp. 4704–4707. doi: 10.1109/IGARSS47720.2021.9553499

[35] FAO. Global Forest Resources Assessment 2006: Main report. Rome: FAO; 2006.

[36] FAO. Global Forest Resources Assessment 2018: Main report. Rome: FAO; 2018.

[37] Grainger A. Difficulties in tracking the long-term global trend in tropical forest area. Proc Natl Acad Sci USA. 2008;105: 818–823. doi: 10.1073/pnas.0703015105 18184819 PMC2206620

[38] Matthews. Evaluation of FAO’s Global Forest Resources Assessment from the user perspective. 2003;53: 42–55.

[39] Hansen MC, Potapov PV, Moore R, Hancher M, Turubanova SA, Tyukavina A, et al. High-Resolution Global Maps of 21st-Century Forest Cover Change. Science. 2013;342: 850–853. doi: 10.1126/science.1244693 24233722

[40] Hansen MC, Stehman SV, Potapov PV. Quantification of global gross forest cover loss. Proc Natl Acad Sci USA. 2010;107: 8650–8655. doi: 10.1073/pnas.0912668107 20421467 PMC2889354

[41] Potapov P, Li X, Hernandez-Serna A, Tyukavina A, Hansen MC, Kommareddy A, et al. Mapping global forest canopy height through integration of GEDI and Landsat data. Remote Sensing of Environment. 2021;253: 112165. doi: 10.1016/j.rse.2020.112165

[42] Potapov P, Turubanova S, Hansen MC, Tyukavina A, Zalles V, Khan A, et al. Global maps of cropland extent and change show accelerated cropland expansion in the twenty-first century. Nat Food. 2022;3: 19–28. doi: 10.1038/s43016-021-00429-z 37118483

[43] CSC. Climate Change Scenarios for the Congo Basin. 2013.

[44] Aloysius NR, Sheffield J, Saiers JE, Li H, Wood EF. Evaluation of historical and future simulations of precipitation and temperature in central Africa from CMIP5 climate models. J Geophys Res Atmos. 2016;121: 130–152. doi: 10.1002/2015JD023656

[45] Fotso-Nguemo TC, Vondou DA, Pokam WM, Djomou ZY, Diallo I, Haensler A, et al. On the added value of the regional climate model REMO in the assessment of climate change signal over Central Africa. Clim Dyn. 2017;49: 3813–3838. doi: 10.1007/s00382-017-3547-7

[46] Anchang JY, Prihodko L, Kaptué AT, Ross CW, Ji W, Kumar SS, et al. Trends in Woody and Herbaceous Vegetation in the Savannas of West Africa. Remote Sensing. 2019;11: 576. doi: 10.3390/rs11050576

[47] Brandt M, Hiernaux P, Rasmussen K, Tucker CJ, Wigneron J-P, Diouf AA, et al. Changes in rainfall distribution promote woody foliage production in the Sahel. Commun Biol. 2019;2: 133. doi: 10.1038/s42003-019-0383-9 31044158 PMC6478729

[48] Hughes AC, Tougeron K, Martin DA, Menga F, Rosado BHP, Villasante S, et al. Smaller human populations are neither a necessary nor sufficient condition for biodiversity conservation. Biological Conservation. 2023;277: 109841. doi: 10.1016/j.biocon.2022.109841

[49] United Nations, Department of Economic and Social Affairs, Population Division. World Population Prospects. United Nations, New York, 10017, USA: United Nations; 2022. Available: file:///D:/Downloads/WPP2022_Data_Sources.pdf

[50] FAO. Trade and Sustainable Forest Management: Impacts and Interactions. Food and Agriculture Organization; 2004. Available: https://enb.iisd.org/crs/tsfm/

[51] Midekisa A, Holl F, Savory DJ, Andrade-Pacheco R, Gething PW, Bennett A, et al. Mapping land cover change over continental Africa using Landsat and Google Earth Engine cloud computing. Schumann GJ-P, editor. PLoS ONE. 2017;12: e0184926. doi: 10.1371/journal.pone.0184926 28953943 PMC5617164

[52] Cook M, Schott J, Mandel J, Raqueno N. Development of an Operational Calibration Methodology for the Landsat Thermal Data Archive and Initial Testing of the Atmospheric Compensation Component of a Land Surface Temperature (LST) Product from the Archive. Remote Sensing. 2014;6: 11244–11266. doi: 10.3390/rs61111244

[53] Goward SN, Masek JG, Loveland TR, Dwyer JL, Williams DL, Arvidson T, et al. Semi-Centennial of Landsat Observations & Pending Landsat 9 Launch. photogramm eng remote sensing. 2021;87: 533–539. doi: 10.14358/PERS.87.8.533

[54] Vermote E, Justice C, Claverie M, Franch B. Preliminary analysis of the performance of the Landsat 8/OLI land surface reflectance product. Remote Sensing of Environment. 2016;185: 46–56. doi: 10.1016/j.rse.2016.04.008 32020955 PMC6999666

[55] Savory DJ, Andrade-Pacheco R, Gething PW, Midekisa A, Bennett A, Sturrock HJW. Intercalibration and Gaussian Process Modeling of Nighttime Lights Imagery for Measuring Urbanization Trends in Africa 2000–2013. Remote Sensing. 2017;9: 713. doi: 10.3390/rs9070713

[56] Elvidge CD, Baugh KE, Kihn EA, Kroehl HW, Davis ER. Mapping city lights with nighttime data from the DMSP Operational Linescan System. Photogramm Eng Remote Sens. 1997;63: 727–734.

[57] Elvidge CD, Baugh KE, Zhizhin M, Hsu F-C. Why VIIRS data are superior to DMSP for mapping nighttime lights. Proceedings of the Asia-Pacific Advanced Network. 2013;35: 62.

[58] Yuh YG, Tracz W, Matthews HD, Turner SE. Application of machine learning approaches for land cover monitoring in northern Cameroon. Ecological Informatics. 2023;74: 101955. doi: 10.1016/j.ecoinf.2022.101955

[59] FAO. On Definitions of Forest and Forest Change, Forest Resources Assessment Programme Working Paper 33, November, 2000. 2000.

[60] Breiman. Random forests. 2001;45: 5–32.

[61] Mellor A, Haywood A, Stone C, Jones S. The Performance of Random Forests in an Operational Setting for Large Area Sclerophyll Forest Classification. Remote Sensing. 2013;5: 2838–2856. doi: 10.3390/rs5062838

[62] Rodriguez-Galiano VF, Ghimire B, Rogan J, Chica-Olmo M, Rigol-Sanchez JP. An assessment of the effectiveness of a random forest classifier for land-cover classification. ISPRS Journal of Photogrammetry and Remote Sensing. 2012;67: 93–104. doi: 10.1016/j.isprsjprs.2011.11.002

[63] Walton. Subpixel urban land cover estimation: comparing cubist, random forests, and support vector regression. Photogrammetric Engineering & Remote Sensing. 2008;74: 1213–1222.

[64] DeFries R, Hansen M, Steininger M, Dubayah R, Sohlberg R, Townshend J. Subpixel forest cover in central Africa from multisensor, multitemporal data. Remote Sensing of Environment. 1997;60: 228–246. doi: 10.1016/S0034-4257(96)00119-8

[65] Hansen MC, Roy DP, Lindquist E, Adusei B, Justice CO, Altstatt A. A method for integrating MODIS and Landsat data for systematic monitoring of forest cover and change in the Congo Basin. Remote Sensing of Environment. 2008;112: 2495–2513. doi: 10.1016/j.rse.2007.11.012

[66] Bwangoy J-RB, Hansen MC, Roy DP, Grandi GD, Justice CO. Wetland mapping in the Congo Basin using optical and radar remotely sensed data and derived topographical indices. Remote Sensing of Environment. 2010;114: 73–86. doi: 10.1016/j.rse.2009.08.004

[67] Midekisa A, Senay GB, Wimberly MC. Multisensor earth observations to characterize wetlands and malaria epidemiology in Ethiopia. Water Resources Research. 2014;50: 8791–8806. doi: 10.1002/2014WR015634 25653462 PMC4303930

[68] Shelestov A, Lavreniuk M, Kussul N, Novikov A, Skakun S. Exploring Google Earth Engine Platform for Big Data Processing: Classification of Multi-Temporal Satellite Imagery for Crop Mapping. Front Earth Sci. 2017;5. doi: 10.3389/feart.2017.00017

[69] Gessner U, Machwitz M, Esch T, Tillack A, Naeimi V, Kuenzer C, et al. Multi-sensor mapping of West African land cover using MODIS, ASAR and TanDEM-X/TerraSAR-X data. Remote Sensing of Environment. 2015;164: 282–297. doi: 10.1016/j.rse.2015.03.029

[70] Olofsson P, Foody GM, Herold M, Stehman SV, Woodcock CE, Wulder MA. Good practices for estimating area and assessing accuracy of land change. Remote Sensing of Environment. 2014;148: 42–57. doi: 10.1016/j.rse.2014.02.015

[71] Chu L, Grafton RQ, Nelson H. Accounting for forest fire risks: global insights for climate change mitigation. Mitig Adapt Strateg Glob Change. 2023;28: 48. doi: 10.1007/s11027-023-10087-0

[72] Cafaro P, Hansson P, Götmark F. Overpopulation is a major cause of biodiversity loss and smaller human populations are necessary to preserve what is left. Biological Conservation. 2022;272: 109646. doi: 10.1016/j.biocon.2022.109646

[73] Juárez-Orozco SM, Siebe C, Fernández y Fernández D. Causes and Effects of Forest Fires in Tropical Rainforests: A Bibliometric Approach. Tropical Conservation Science. 2017;10: 194008291773720. doi: 10.1177/1940082917737207

[74] Kleinschroth F, Laporte N, Laurance WF, Goetz SJ, Ghazoul J. Road expansion and persistence in forests of the Congo Basin. Nat Sustain. 2019;2: 628–634. doi: 10.1038/s41893-019-0310-6

[75] Steffen W, Broadgate W, Deutsch L, Gaffney O, Ludwig C. The trajectory of the Anthropocene: The Great Acceleration. The Anthropocene Review. 2015;2: 81–98. doi: 10.1177/2053019614564785

[76] Nkrumah K. Neo-colonialism: the last stage of imperialism. 6. print. New York: International Publ; 1976.

[77] Laurance W. EMERGING THREATS TO TROPICAL FORESTS. ANNALS OF THE MISSOURI BOTANICAL GARDEN. 2015;100: 159–169. doi: 10.3417/2011087

[78] Laurance WF, Goosem M, Laurance SGW. Impacts of roads and linear clearings on tropical forests. Trends in Ecology & Evolution. 2009;24: 659–669. doi: 10.1016/j.tree.2009.06.009 19748151

[79] Laurance WF, Carolina Useche D, Rendeiro J, Kalka M, Bradshaw CJA, Sloan SP, et al. Averting biodiversity collapse in tropical forest protected areas. Nature. 2012;489: 290–294. doi: 10.1038/nature11318 22832582

[80] Laurance WF, Clements GR, Sloan S, O’Connell CS, Mueller ND, Goosem M, et al. A global strategy for road building. Nature. 2014;513: 229–232. doi: 10.1038/nature13717 25162528

[81] Weinhold D, Reis E. Transportation costs and the spatial distribution of land use in the Brazilian Amazon. Global Environmental Change. 2008;18: 54–68. doi: 10.1016/j.gloenvcha.2007.06.004

[82] Nuissl H, Siedentop S. Urbanisation and Land Use Change. In: Weith T, Barkmann T, Gaasch N, Rogga S, Strauß C, Zscheischler J, editors. Sustainable Land Management in a European Context. Cham: Springer International Publishing; 2021. pp. 75–99. doi: 10.1007/978-3-030-50841-8_5

[83] Sang CC, Olago DO, Nyumba TO, Marchant R, Thorn JPR. Assessing the Underlying Drivers of Change over Two Decades of Land Use and Land Cover Dynamics along the Standard Gauge Railway Corridor, Kenya. Sustainability. 2022;14: 6158. doi: 10.3390/su14106158

[84] Sarfo I, Shuoben B, Otchwemah HB, Darko G, Kedjanyi EAG, Oduro C, et al. Validating local drivers influencing land use cover change in Southwestern Ghana: a mixed-method approach. Environ Earth Sci. 2022;81: 367. doi: 10.1007/s12665-022-10481-y 35875811 PMC9296760

[85] Olén NB, Lehsten V. High-resolution global population projections dataset developed with CMIP6 RCP and SSP scenarios for year 2010–2100. Data Brief. 2022;40: 107804. doi: 10.1016/j.dib.2022.107804 35071702 PMC8762352

[86] Eyring V, Bony S, Meehl GA, Senior CA, Stevens B, Stouffer RJ, et al. Overview of the Coupled Model Intercomparison Project Phase 6 (CMIP6) experimental design and organization. Geosci Model Dev. 2016;9: 1937–1958. doi: 10.5194/gmd-9-1937-2016

[87] Thrasher B, Wang W, Michaelis A, Melton F, Lee T, Nemani R. NASA Global Daily Downscaled Projections, CMIP6. Sci Data. 2022;9: 262. doi: 10.1038/s41597-022-01393-4 35654862 PMC9163132

[88] Eastman JR, Toledano J. A Short Presentation of the Land Change Modeler (LCM). In: Camacho Olmedo MT, Paegelow M, Mas J-F, Escobar F, editors. Geomatic Approaches for Modeling Land Change Scenarios. Cham: Springer International Publishing; 2018. pp. 499–505. doi: 10.1007/978-3-319-60801-3_36

[89] TerrSet. TerrSet 2020 Geospatial Monitoring and Modeling Software. Clark University 950 Main St., Worcester MA 01610 USA: Clark Labs; 2020. Available: https://clarklabs.org/terrset/

[90] Atkinson PM, Tatnall ARL. Introduction Neural networks in remote sensing. International Journal of Remote Sensing. 1997;18: 699–709. doi: 10.1080/014311697218700

[91] Gibson L, Münch Z, Palmer A, Mantel S. Future land cover change scenarios in South African grasslands–implications of altered biophysical drivers on land management. Heliyon. 2018;4: e00693. doi: 10.1016/j.heliyon.2018.e00693 30035238 PMC6052193

[92] Gagniuc PA. Markov chains: from theory to implementation and experimentation. Hoboken, NJ: John Wiley & Sons; 2017.

[93] Pérez-Vega A, Mas J-F, Ligmann-Zielinska A. Comparing two approaches to land use/cover change modeling and their implications for the assessment of biodiversity loss in a deciduous tropical forest. Environmental Modelling & Software. 2012;29: 11–23. doi: 10.1016/j.envsoft.2011.09.011

[94] IPCC. Synthesis Report. Contribution of Working Groups I, II and III to the Fifth Assessment Report of the Intergovernmental Panel on Climate Change. Geneva; 2014.

[95] Kirilenko AP, Sedjo RA. Climate change impacts on forestry. Proc Natl Acad Sci USA. 2007;104: 19697–19702. doi: 10.1073/pnas.0701424104 18077403 PMC2148360

[96] Da Cruz DC, Benayas JMR, Ferreira GC, Santos SR, Schwartz G. An overview of forest loss and restoration in the Brazilian Amazon. New Forests. 2021;52: 1–16. doi: 10.1007/s11056-020-09777-3

[97] Gaveau DLA, Sloan S, Molidena E, Yaen H, Sheil D, Abram NK, et al. Four Decades of Forest Persistence, Clearance and Logging on Borneo. Bawa K, editor. PLoS ONE. 2014;9: e101654. doi: 10.1371/journal.pone.0101654 25029192 PMC4100734

[98] Mosnier et al. Modelling Land Use Changes in the Democratic Republic of the Congo 2000–2030. 2016. Available: www.redd-pac.org

[99] Achancho V. Review and analysis of national investment strategies and agricultural policies in central Africa: the Case of Cameroun. Elbehri A. (ed.). Rebuilding West Africa’s Food Potential. A. Elbehri (ed.). FAO/IFAD; 2013. pp. 117–148. Available: https://www.fao.org/3/i3222e/i3222e04.pdf

[100] Ordway EM, Naylor RL, Nkongho RN, Lambin EF. Oil palm expansion and deforestation in Southwest Cameroon associated with proliferation of informal mills. Nat Commun. 2019;10: 114. doi: 10.1038/s41467-018-07915-2 30631076 PMC6328567

[101] Sassen M, van Soesbergen A, Arnell AP, Scott E. Patterns of (future) environmental risks from cocoa expansion and intensification in West Africa call for context specific responses. Land Use Policy. 2022;119: 106142. doi: 10.1016/j.landusepol.2022.106142

[102] Rainforest foundation. Roads to Ruin: the emerging impacts of infrastructure development in Congo Basin forests. 2021. Available: https://www.rainforestfoundationuk.org/wp-content/uploads/2021/10/infrastructure-report.pdf

[103] Durrieu de Madron et al. Dégâts d’exploitation et de débardage en fonction de l’intensité d’exploitation en forêt dense humide d’Afrique Centrale Bois et Forêts des Tropiques. 2000. Available: https://revues.cirad.fr/index.php/BFT/article/view/20052

[104] Ruiz Pérez M, Ezzine de Blas D, Nasi R, Sayer JA, Sassen M, Angoué C, et al. Logging in the Congo Basin: A multi-country characterization of timber companies. Forest Ecology and Management. 2005;214: 221–236. doi: 10.1016/j.foreco.2005.04.020

[105] Cerutti PO, Tacconi L. Forests, Illegality, and Livelihoods in Cameroon. Situ Gede, Sindang Barang, Bogor Barat 16680, Indonesia.: Center for International Forestry Research Jl (CIFOR); 2006. Available: https://pdfs.semanticscholar.org/304a/d82ad40d36fa0415b7c959ef9d09a9d2000c.pdf

[106] Eba’a Atyi R. Cameroon’s Logging Industry: Structure, Economic Importance and Effects of Devaluation. 1998. Report No.: 14. Available: https://www.cifor.org/publications/pdf_files/OccPapers/OP-14.pdf

[107] Lescuyer G, P.O. C, P. T, F. B, B. A-A, R. T, et al. The domestic market for small-scale chainsaw milling in the Democratic Republic of Congo: Present situation, opportunities and challenges. Center for International Forestry Research (CIFOR); 2014. doi: 10.17528/cifor/005040

[108] Kolden CA, Abatzoglou JT. Wildfire Consumption and Interannual Impacts by Land Cover in Alaskan Boreal Forest. fire ecol. 2012;8: 98–114. doi: 10.4996/fireecology.0801098

[109] Stralberg D, Wang X, Parisien M-A, Robinne F-N, Sólymos P, Mahon CL, et al. Wildfire-mediated vegetation change in boreal forests of Alberta, Canada. Ecosphere. 2018;9: e02156. doi: 10.1002/ecs2.2156

[110] Ameray A, Cavard X, Bergeron Y. Climate change may increase Quebec boreal forest productivity in high latitudes by shifting its current composition. Front For Glob Change. 2023;6: 1020305. doi: 10.3389/ffgc.2023.1020305

[111] Cox PM, Betts RA, Collins M, Harris PP, Huntingford C, Jones CD. Amazonian forest dieback under climate-carbon cycle projections for the 21st century. Theor Appl Climatol. 2004;78. doi: 10.1007/s00704-004-0049-4

[112] Chaturvedi RK, Gopalakrishnan R, Jayaraman M, Bala G, Joshi NV, Sukumar R, et al. Impact of climate change on Indian forests: a dynamic vegetation modeling approach. Mitig Adapt Strateg Glob Change. 2011;16: 119–142. doi: 10.1007/s11027-010-9257-7

[113] Upgupta S, Sharma J, Jayaraman M, Kumar V, Ravindranath NH. Climate change impact and vulnerability assessment of forests in the Indian Western Himalayan region: A case study of Himachal Pradesh, India. Climate Risk Management. 2015;10: 63–76. doi: 10.1016/j.crm.2015.08.002

[114] Beaumont LJ, Duursma D. Global Projections of 21st Century Land-Use Changes in Regions Adjacent to Protected Areas. Evans DM, editor. PLoS ONE. 2012;7: e43714. doi: 10.1371/journal.pone.0043714 22952744 PMC3431375

[115] Chen M, Vernon CR, Graham NT, Hejazi M, Huang M, Cheng Y, et al. Global land use for 2015–2100 at 0.05° resolution under diverse socioeconomic and climate scenarios. Sci Data. 2020;7: 320. doi: 10.1038/s41597-020-00669-x 33009403 PMC7532189

[116] Hurtt GC, Chini LP, Frolking S, Betts RA, Feddema J, Fischer G, et al. Harmonization of land-use scenarios for the period 1500–2100: 600 years of global gridded annual land-use transitions, wood harvest, and resulting secondary lands. Climatic Change. 2011;109: 117–161. doi: 10.1007/s10584-011-0153-2

[117] Gomez-Zavaglia A, Mejuto JC, Simal-Gandara J. Mitigation of emerging implications of climate change on food production systems. Food Research International. 2020;134: 109256. doi: 10.1016/j.foodres.2020.109256 32517948 PMC7176580

[118] Mangaza L, Sonwa DJ, Batsi G, Ebuy J, Kahindo J-M. Building a framework towards climate-smart agriculture in the Yangambi landscape, Democratic Republic of Congo (DRC). IJCCSM. 2021;13: 320–338. doi: 10.1108/IJCCSM-08-2020-0084

[119] Dove et al. Climate Risk Country Profile: Congo, Democratic Republic. Washington, WA, USA: In The World Bank Group; World Bank; 2021. Available: https://climateknowledgeportal.worldbank.org/sites/default/files/2021-06/15883-WB_Congo%2C%20Democratic%20Republic%20Country%20Profile-WEB.pdf

[120] Sonwa DJ, Nkem JN, Idinoba ME, Bele MY, Jum C. Building regional priorities in forests for development and adaptation to climate change in the Congo Basin. Mitig Adapt Strateg Glob Change. 2012;17: 441–450. doi: 10.1007/s11027-011-9335-5

[121] Karume K, Mondo JM, Chuma GB, Ibanda A, Bagula EM, Aleke AL, et al. Current Practices and Prospects of Climate-Smart Agriculture in Democratic Republic of Congo: A Review. Land. 2022;11: 1850. doi: 10.3390/land11101850

[122] Megevand C, Mosnier A, Hourticq J, Sanders K, Doetinchem N, Streck C. Deforestation Trends in the Congo Basin: Reconciling Economic Growth and Forest Protection. The World Bank; 2013. doi: 10.1596/978-0-8213-9742-8

[123] Abegunde VO, Obi A. The Role and Perspective of Climate Smart Agriculture in Africa: A Scientific Review. Sustainability. 2022;14: 2317. doi: 10.3390/su14042317

[124] Ariom TO, Dimon E, Nambeye E, Diouf NS, Adelusi OO, Boudalia S. Climate-Smart Agriculture in African Countries: A Review of Strategies and Impacts on Smallholder Farmers. Sustainability. 2022;14: 11370. doi: 10.3390/su141811370

[125] Ogunyiola A, Gardezi M, Vij S. Smallholder farmers’ engagement with climate smart agriculture in Africa: role of local knowledge and upscaling. Climate Policy. 2022;22: 411–426. doi: 10.1080/14693062.2021.2023451

[126] Ziervogel G, Cartwright A, Tas A, Adejuwon J, Zermoglio F, Shale M, et al. Climate change and adaptation in African agriculture. Stockholm environment institute; 2008 Mar pp. 17–19.

[127] Mendelsohn R, Dinar A, Dalfelt A. Climate change impacts on African agriculture. Preliminary analysis prepared for the World Bank, Washington, Districtof Columbia. 2000 p. 25.

[128] Lema MA, Majule AE. Impacts of climate change, variability and adaptation strategies on agriculture in semi arid areas of Tanzania: The case of Manyoni District in Singida Region, Tanzania. Afr J Environ Sci Technol. 2009;3: 206–218. doi: 10.5897/AJEST09.099

[129] Abernethy K, Maisels F, White LJT. Environmental Issues in Central Africa. Annu Rev Environ Resour. 2016;41: 1–33. doi: 10.1146/annurev-environ-110615-085415

[130] Nguyen H, Chu L, Harper RJ, Dell B, Hoang H. Mangrove-shrimp farming: A triple-win approach for communities in the Mekong River Delta. Ocean & Coastal Management. 2022;221: 106082. doi: 10.1016/j.ocecoaman.2022.106082

[131] Chu L, Grafton RQ, Nguyen H. A global analysis of the break-even prices to reduce atmospheric carbon dioxide via forest plantation and avoided deforestation. Forest Policy and Economics. 2022;135: 102666. doi: 10.1016/j.forpol.2021.102666

[132] Miller DC, Rana P, Nakamura K, Irwin S, Cheng SH, Ahlroth S, et al. A global review of the impact of forest property rights interventions on poverty. Global Environmental Change. 2021;66: 102218. doi: 10.1016/j.gloenvcha.2020.102218

[133] Karsenty A, Ongolo S. Can “fragile states” decide to reduce their deforestation? The inappropriate use of the theory of incentives with respect to the REDD mechanism. Forest Policy and Economics. 2012;18: 38–45. doi: 10.1016/j.forpol.2011.05.006

[134] Barbier EB, Tesfaw AT. Tenure Constraints and Carbon Forestry in Africa. American J Agri Economics. 2013;95: 964–975. doi: 10.1093/ajae/aat014

[135] Liboiron M, Cotter R. Review of participation of Indigenous peoples in plastics pollution governance. Camb prisms Plast. 2023;1: e16. doi: 10.1017/plc.2023.16

[136] Gilmour DA. Forty years of community-based forestry: a review of its extent and effectiveness. Rome: Food and agriculture organization of the United Nations; 2016.

[137] World Bank. Securing Forest Tenure Rights for Rural Development: An Analytical Framework. 2019.

[138] Ginsburg C, Keene S. At a crossroads: consequential trends in recognition of community-based forest tenure from 2002–2017. China Economic Journal. 2020;13: 223–248. doi: 10.1080/17538963.2020.1755129

[139] Baynes J, Herbohn J, Smith C, Fisher R, Bray D. Key factors which influence the success of community forestry in developing countries. Global Environmental Change. 2015;35: 226–238. doi: 10.1016/j.gloenvcha.2015.09.011

[140] Mukul SA, Herbohn J, Rashid AZMM, Uddin MB. Comparing the effectiveness of forest law enforcement and economic incentives to prevent illegal logging in Bangladesh. Int Forest Rev. 2014;16: 363–375. doi: 10.1505/146554814812572485

[141] Mukul SA, Rashid AZMM, Quazi SA, Uddin MB, Fox J. Local peoples’ responses to co-management regime in protected areas: A case study from Satchari National Park, Bangladesh. Forests, Trees and Livelihoods. 2012;21: 16–29. doi: 10.1080/14728028.2012.669132

[142] Oldekop JA, Sims KRE, Karna BK, Whittingham MJ, Agrawal A. Reductions in deforestation and poverty from decentralized forest management in Nepal. Nat Sustain. 2019;2: 421–428. doi: 10.1038/s41893-019-0277-3

[143] Hajjar R, Oldekop JA, Cronkleton P, Newton P, Russell AJM, Zhou W. A global analysis of the social and environmental outcomes of community forests. Nat Sustain. 2020;4: 216–224. doi: 10.1038/s41893-020-00633-y

[144] Midgley SJ, Stevens PR, Arnold RJ. Hidden assets: Asia’s smallholder wood resources and their contribution to supply chains of commercial wood. Australian Forestry. 2017;80: 10–25. doi: 10.1080/00049158.2017.1280750

[145] Frey GE, Cubbage FW, Ha TTT, Davis RR, Carle JB, Thon VX, et al. Financial analysis and comparison of smallholder forest and state forest enterprise plantations in Central Vietnam. int forest rev. 2018;20: 181–198. doi: 10.1505/146554818823767582

[146] Tadesse W, Gezahgne A, Tesema T, Shibabaw B, Tefera B, Kassa H. Plantation forests in Amhara region: challenges and best measures for future improvements. World J Agric Res. 2019;7: 149–157.

[147] Arvola A, Brockhaus M, Kallio M, Pham TT, Chi DTL, Long HT, et al. What drives smallholder tree growing? Enabling conditions in a changing policy environment. Forest Policy and Economics. 2020;116: 102173. doi: 10.1016/j.forpol.2020.102173

[148] Kimambo NE, L’Roe J, Naughton-Treves L, Radeloff VC. The role of smallholder woodlots in global restoration pledges–Lessons from Tanzania. Forest Policy and Economics. 2020;115: 102144. doi: 10.1016/j.forpol.2020.102144

[149] Kirumira D, Baranga D, Hartter J, Valenta K, Tumwesigye C, Kagoro W, et al. Evaluating a Union between Health Care and Conservation: a Mobile Clinic Improves Park-People Relations, Yet Poaching Increases. Conservat Soc. 2019;17: 51. doi: 10.4103/cs.cs_17_72

[150] Turner‐Skoff JB, Cavender N. The benefits of trees for livable and sustainable communities. Plants People Planet. 2019;1: 323–335. doi: 10.1002/ppp3.39

[151] Miller: Daniel C, Mansourian S, Wildburger C. Forests, trees and the eradication of poverty: potential and limitations. A global assessment report. 2020.

[152] Miller DC, Hajjar R. Forests as pathways to prosperity: Empirical insights and conceptual advances. World Development. 2020;125: 104647. doi: 10.1016/j.worlddev.2019.104647

[153] Konijnendijk C, Devkota D, Mansourian S, Wildburger C. Forests and Trees for Human Health: Pathways, Impacts, Challenges and Response Options. A Global Assessment Report. 8th Scientific assessment undertaken in the framework of the Global Forest Expert Panels (GFEP) initiative, 21 March 2023. 2023.

[154] Jones IJ, MacDonald AJ, Hopkins SR, Lund AJ, Liu ZY-C, Fawzi NI, et al. Improving rural health care reduces illegal logging and conserves carbon in a tropical forest. Proc Natl Acad Sci USA. 2020;117: 28515–28524. doi: 10.1073/pnas.2009240117 33106399 PMC7668090

[155] Quentin Grafton R, Chu HL, Nelson H, Bonnis G. Household transport choices: New empirical evidence and policy implications for sustainable behaviour. 2024 Jul. Report No.: 246. doi: 10.1787/0e8469ed-en

[156] Chu L, Quentin Grafton R, Keenan R. Increasing Conservation Efficiency While Maintaining Distributive Goals With the Payment for Environmental Services. Ecological Economics. 2019;156: 202–210. doi: 10.1016/j.ecolecon.2018.10.003

[157] Austin KG, Baker JS, Sohngen BL, Wade CM, Daigneault A, Ohrel SB, et al. The economic costs of planting, preserving, and managing the world’s forests to mitigate climate change. Nat Commun. 2020;11: 5946. doi: 10.1038/s41467-020-19578-z 33262324 PMC7708837

[158] Busch J, Engelmann J. Cost-effectiveness of reducing emissions from tropical deforestation, 2016–2050. Environ Res Lett. 2017;13: 015001. doi: 10.1088/1748-9326/aa907c

[159] Kindermann G, Obersteiner M, Sohngen B, Sathaye J, Andrasko K, Rametsteiner E, et al. Global cost estimates of reducing carbon emissions through avoided deforestation. Proc Natl Acad Sci USA. 2008;105: 10302–10307. doi: 10.1073/pnas.0710616105 18650377 PMC2483236

[160] Ipcc. Global Warming of 1.5°C: IPCC Special Report on Impacts of Global Warming of 1.5°C above Pre-industrial Levels in Context of Strengthening Response to Climate Change, Sustainable Development, and Efforts to Eradicate Poverty. 1st ed. Cambridge University Press; 2022. doi: 10.1017/9781009157940

[161] Yuh YG, N’Goran KP, Beukou GB, Wendefeuer J, Neba TF, Ndotar AM, et al. Recent decline in suitable large mammal habitats within the Dzanga Sangha Protected Areas, Central African Republic. Global Ecology and Conservation. 2023;42: e02404. doi: 10.1016/j.gecco.2023.e02404

[162] Eisen J, Counsell S, Thornberry F. Rainforest Foundation UK–Rethinking community based forest management in the Congo Basin, NOV 2014. Rainforest Foundation UK; 2014. Available: https://www.rainforestfoundationuk.org/media.ashx/rethinkingcommunitybasedforestmanagementinthecongobasinnovember2014.pdf

[163] Naime J, Angelsen A, Molina-Garzón A, Carrilho CD, Selviana V, Demarchi G, et al. Enforcement and inequality in collective PES to reduce tropical deforestation: Effectiveness, efficiency and equity implications. Global Environmental Change. 2022;74: 102520. doi: 10.1016/j.gloenvcha.2022.102520

[164] Naeem S, Ingram JC, Varga A, Agardy T, Barten P, Bennett G, et al. Get the science right when paying for nature’s services. Science. 2015;347: 1206–1207. doi: 10.1126/science.aaa1403 25766222

[165] Schomers S, Matzdorf B. Payments for ecosystem services: A review and comparison of developing and industrialized countries. Ecosystem Services. 2013;6: 16–30. doi: 10.1016/j.ecoser.2013.01.002

[166] Calvet-Mir L, Corbera E, Martin A, Fisher J, Gross-Camp N. Payments for ecosystem services in the tropics: a closer look at effectiveness and equity. Current Opinion in Environmental Sustainability. 2015;14: 150–162. doi: 10.1016/j.cosust.2015.06.001

[167] Brown D, Seymour F, Peskett L. How do we achieve REDD co-benefits and avoid doing harm. Moving ahead with REDD: issues, options and implications. 2008; 107–118.

[168] Andersson KP, Smith SM, Alston LJ, Duchelle AE, Mwangi E, Larson AM, et al. Wealth and the distribution of benefits from tropical forests: Implications for REDD+. Land Use Policy. 2018;72: 510–522. doi: 10.1016/j.landusepol.2018.01.012

[169] G. W, M. B, M. M, C. P, T.T. P. Equity, REDD+ and Benefit Sharing in Social Forestry. Center for International Forestry Research (CIFOR); 2016. doi: 10.17528/cifor/006127

