# Supplementary figures and images for: Monitoring forest cover and land use change in the Congo Basin under IPCC climate change scenarios
Source: PLoS One. 2024 Dec 2;19(12):e0311816. doi: 10.1371/journal.pone.0311816 (PMC11611213; doi:10.1371/journal.pone.0311816)

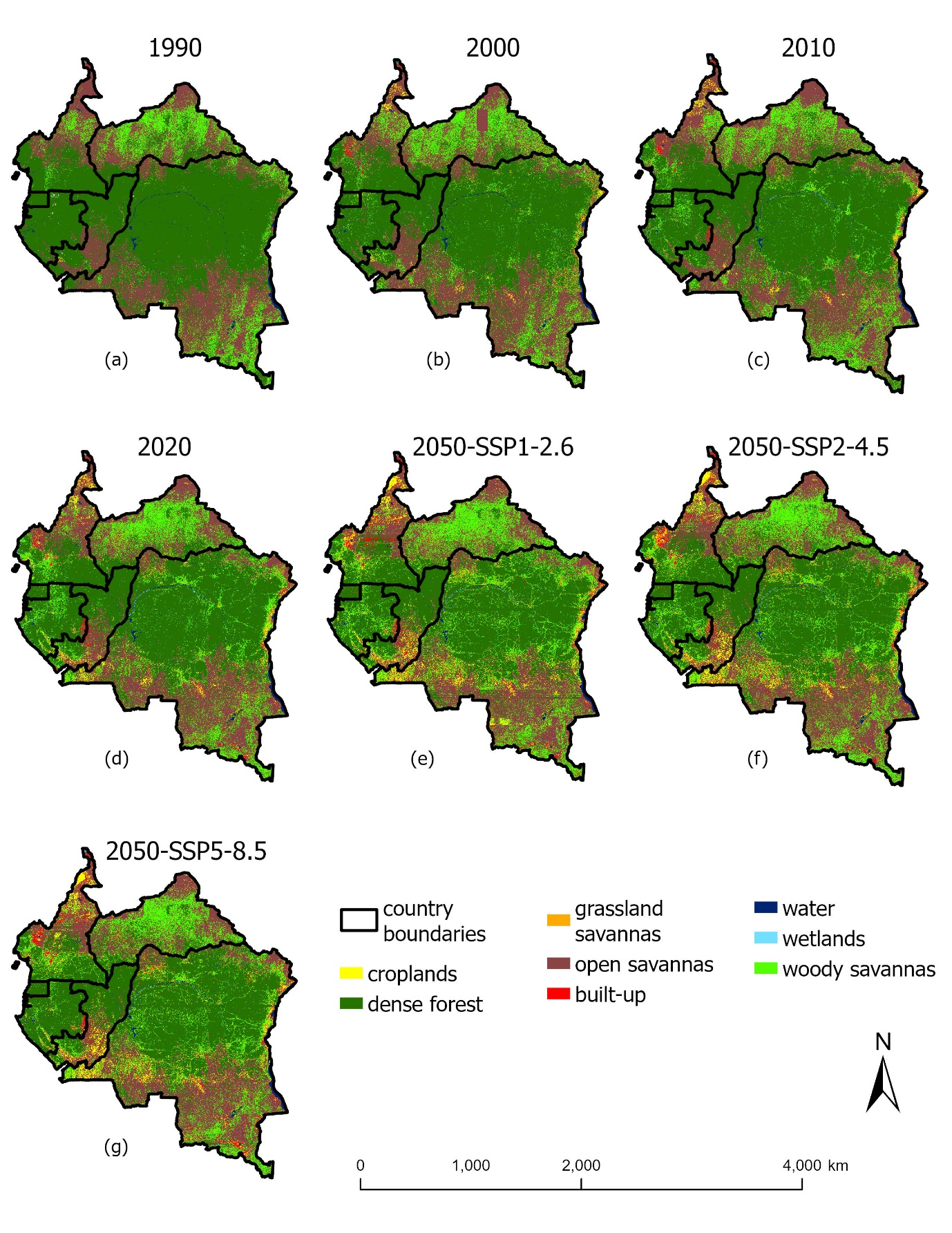

Supplement: S1 Fig — Map generated with the RF Machine learning model, using the UUSGS Landsat 5 and 7 Collection 2 Level 2 data, freely available for public use with no data restrictions nor permissions required: https://www.usgs.gov/faqs/are-there-any-restrictions-use-or-redistribution-landsat-data. (TIF) [file pone.0311816.s001.tif]

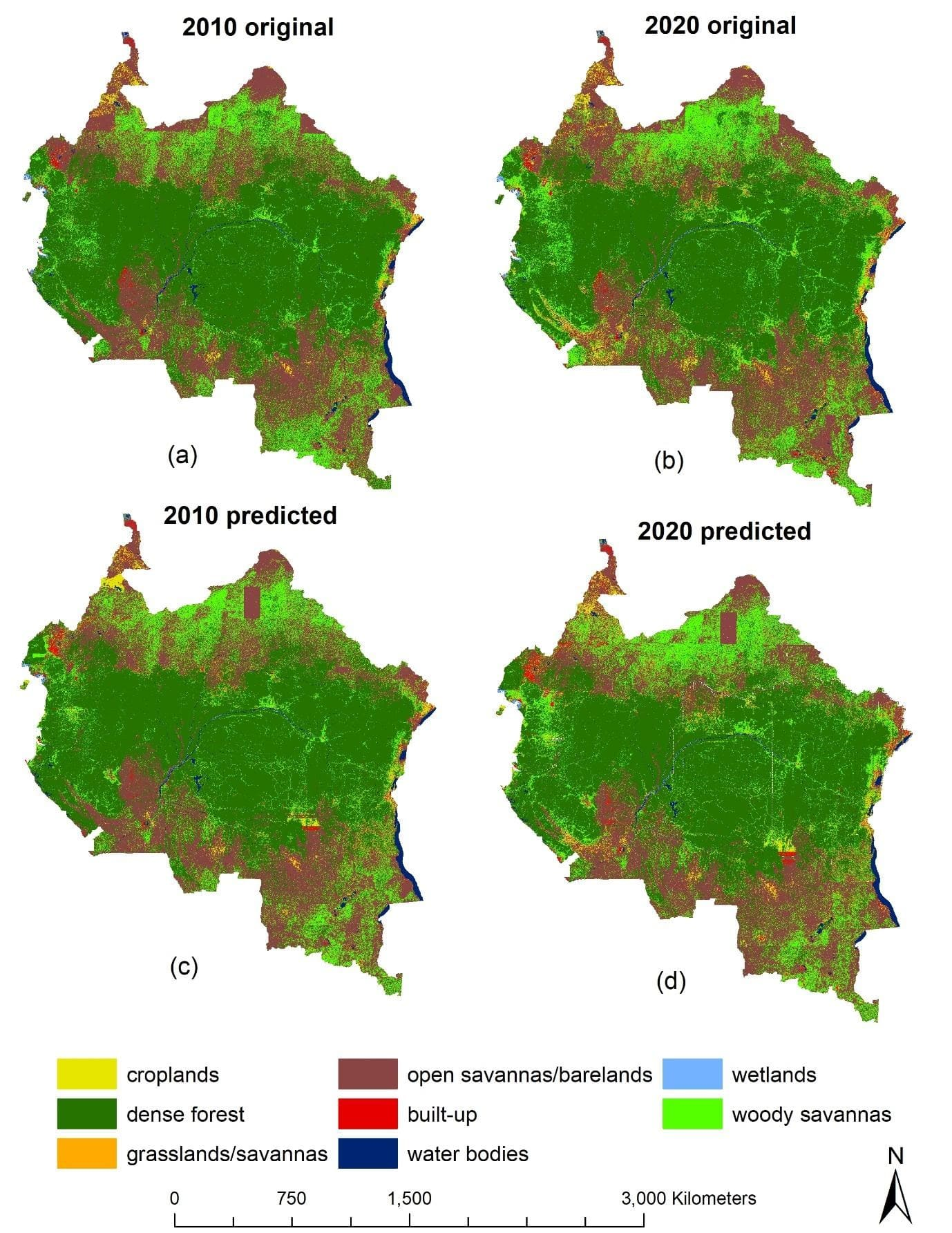

Supplement: S2 Fig — Map products show strong correlations between the original and predicted datasets as quantified in S12A and S12B Table, suggesting the reliability of the ILCM in predicting LULCC. Map generated with the RF Machine learning model, using the UUSGS Landsat 5 and 7 Collection 2 Level 2 data, freely available for public use with no data restrictions nor permissions required: https://www.usgs.gov/faqs/are-there-any-restrictions-use-or-redistribution-landsat-data. (TIF) [file pone.0311816.s002.tif]

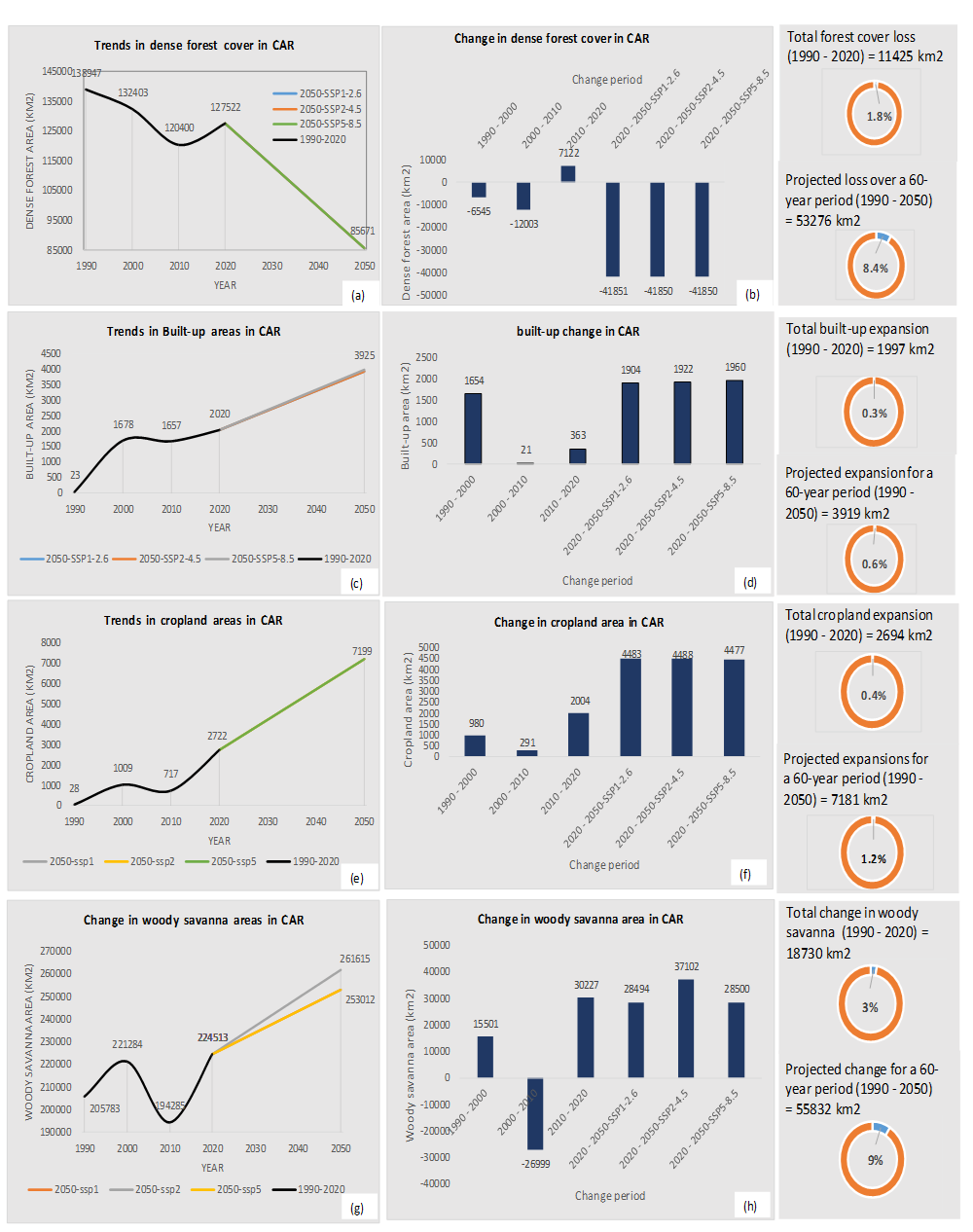

Supplement: S3 Fig — Figures a and b show trends and changes observed in dense forest areas, including information on total and projected forest cover loss for a 60-year period (1990–2050). Figures c and d, e and f, and g and h show similar trend and change information for built-up areas, croplands, and woody savannas respectively. (TIF) [file pone.0311816.s003.tif]

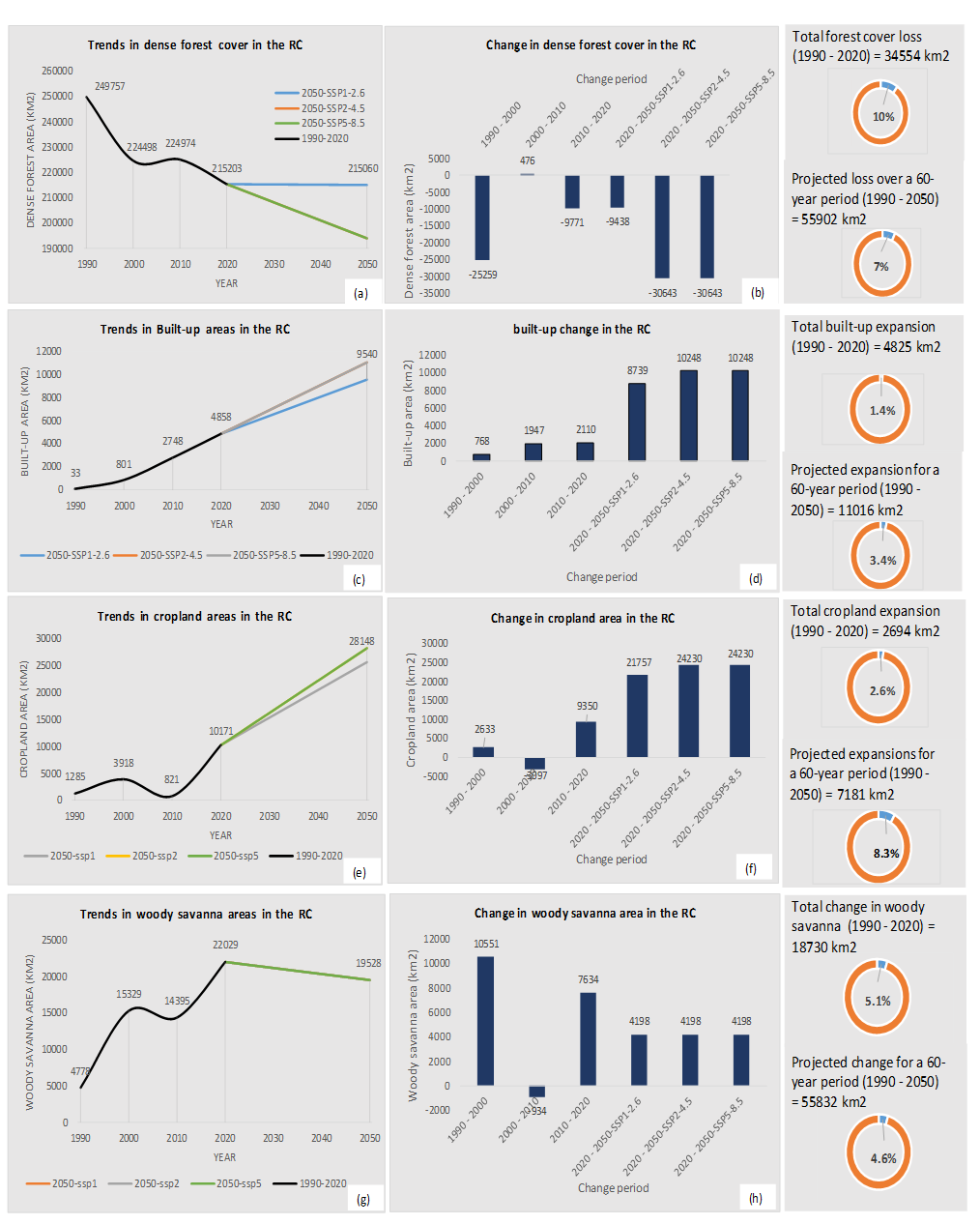

Supplement: S4 Fig — Figures a and b show trends and changes observed in dense forest areas, including information on total and projected forest cover loss for a 60-year period (1990–2050). Figures c and d, e and f, and g and h show similar trend and change information for built-up areas, croplands, and woody savannas respectively. (TIF) [file pone.0311816.s004.tif]

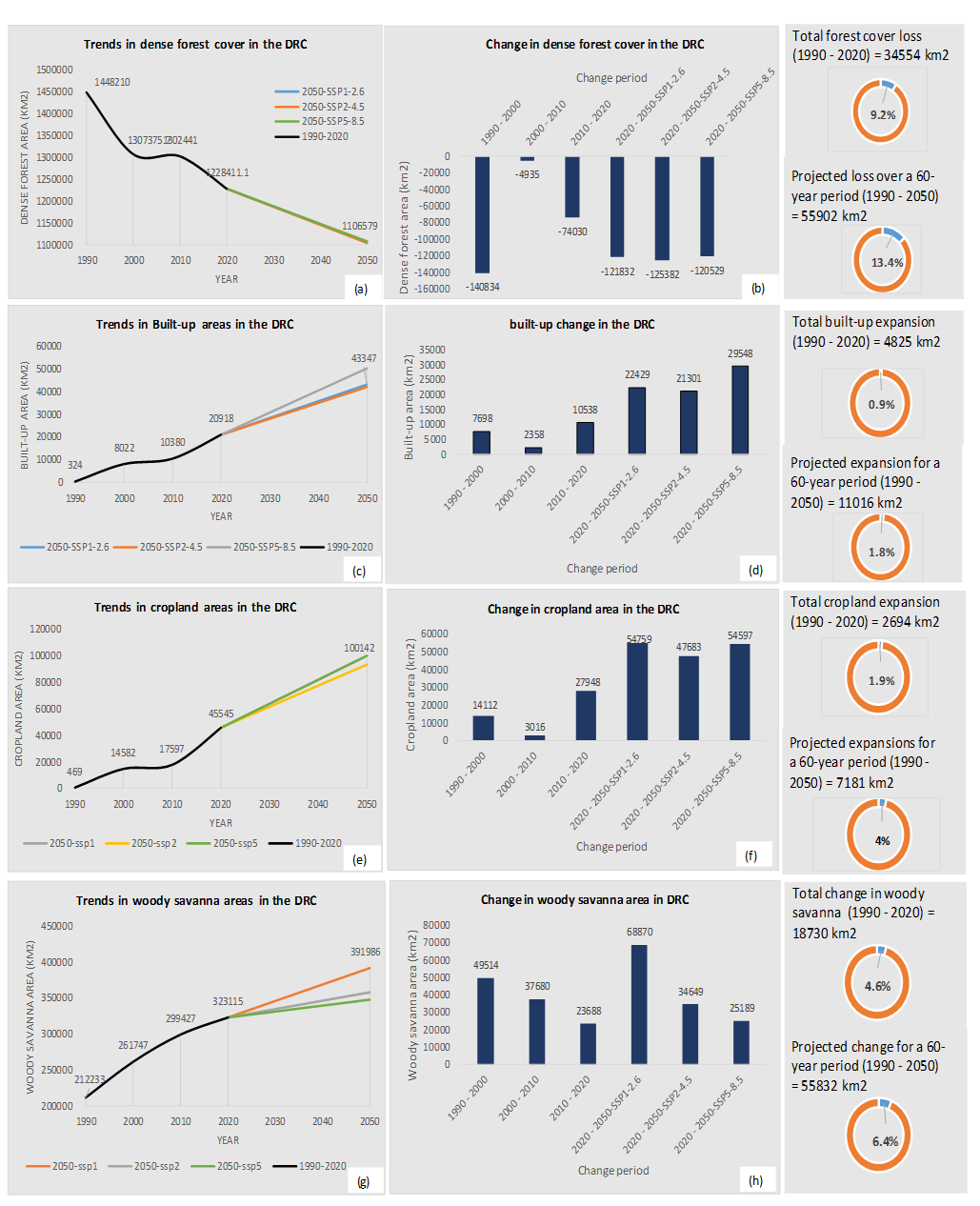

Supplement: S5 Fig — Figures a and b show trends and changes observed in dense forest areas, including information on total and projected forest cover loss for a 60-year period (1990–2050). Figures c and d, e and f, and g and h show similar trend and change information for built-up areas, croplands, and woody savannas respectively. (TIF) [file pone.0311816.s005.tif]

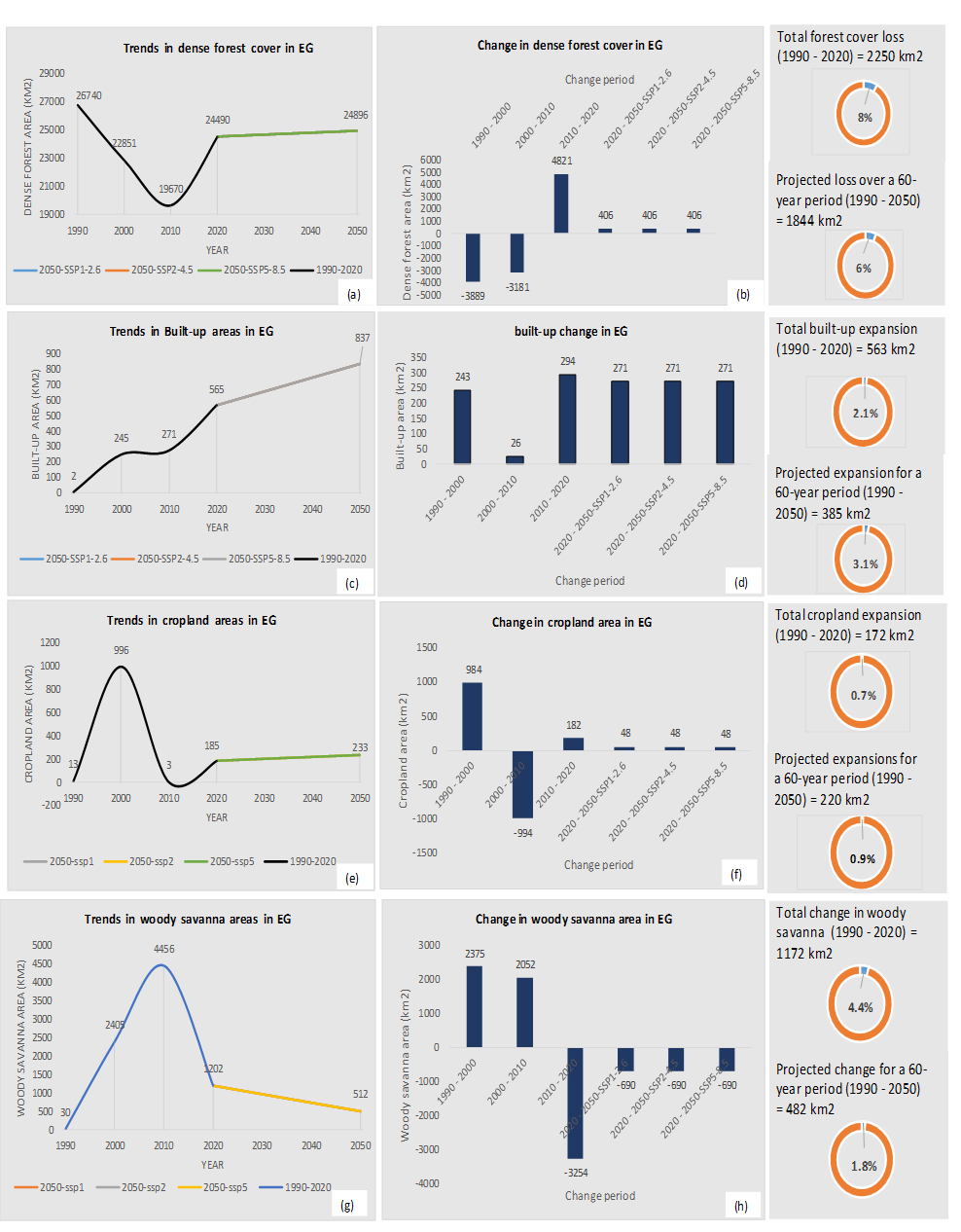

Supplement: S6 Fig — Figures a and b show trends and changes observed in dense forest areas, including information on total and projected forest cover loss for a 60-year period (1990–2050). Figures c and d, e and f, and g and h show similar trend and change information for built-up areas, croplands, and woody savannas respectively. (TIF) [file pone.0311816.s006.tif]

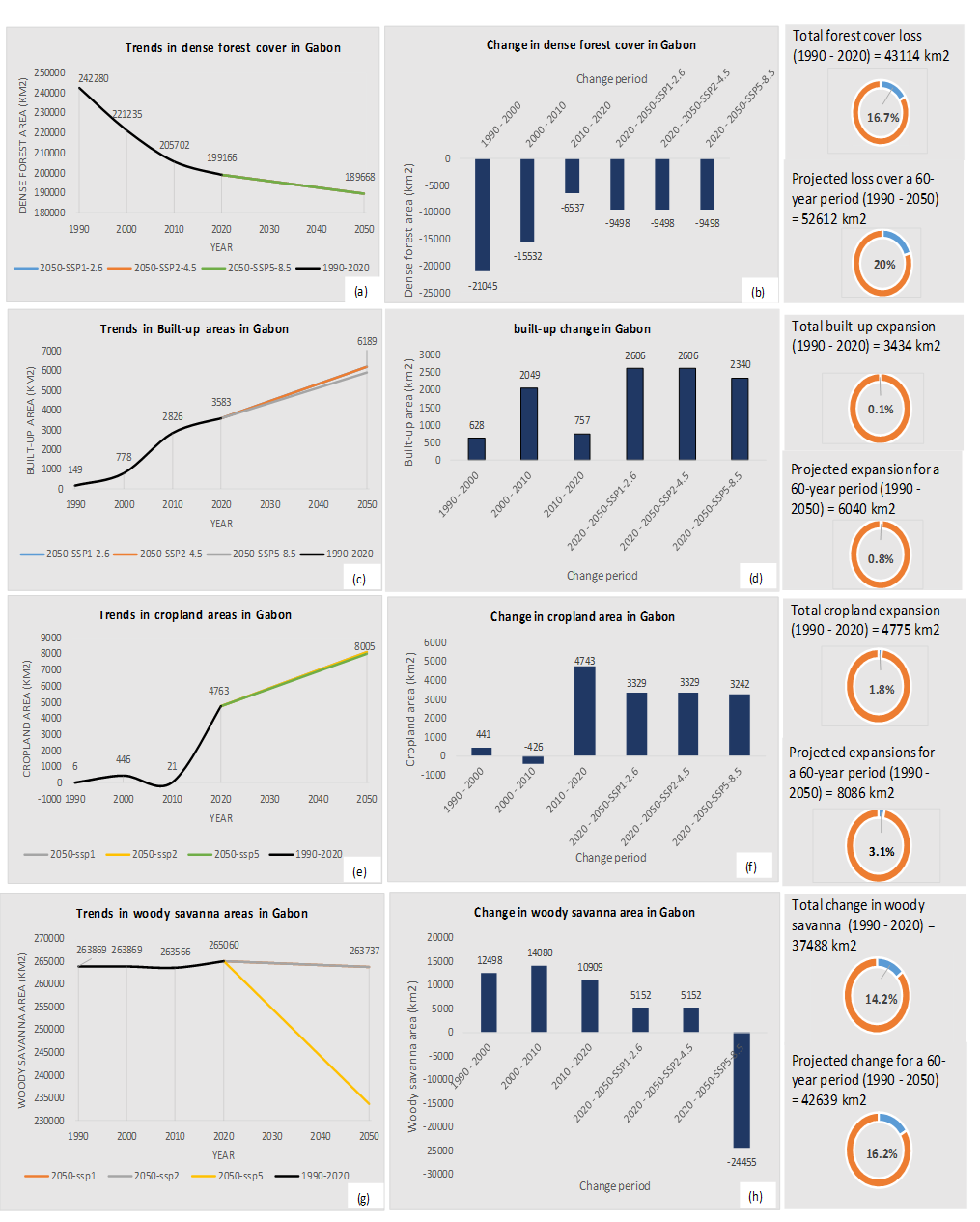

Supplement: S7 Fig — Figures a and b show trends and changes observed in dense forest areas, including information on total and projected forest cover loss for a 60-year period (1990–2050). Figures c and d, e and f, and g and h show similar trend and change information for built-up areas, croplands, and woody savannas respectively. (TIF) [file pone.0311816.s007.tif]

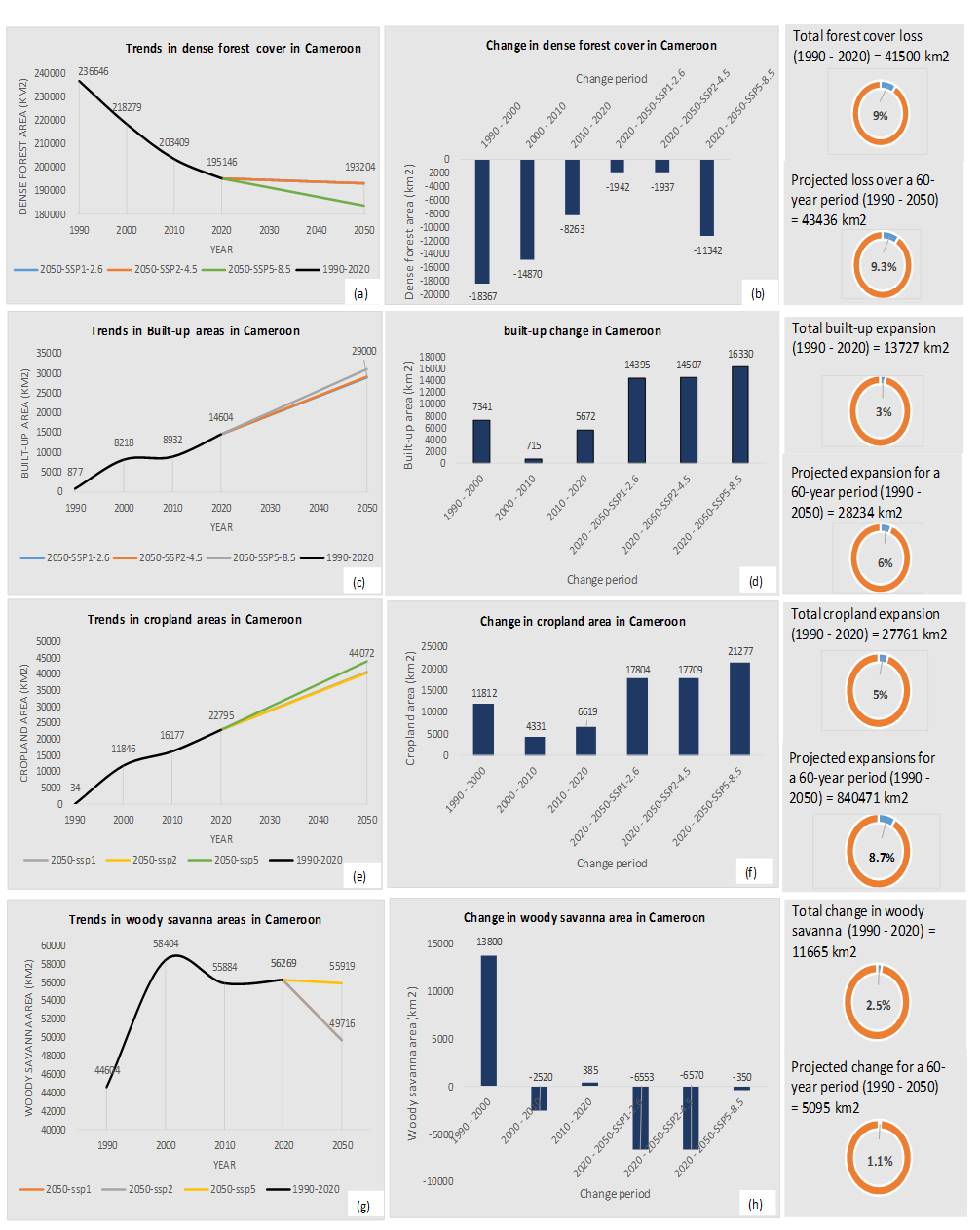

Supplement: S8 Fig — Figures a and b show trends and changes observed in dense forest areas, including information on total and projected forest cover loss for a 60-year period (1990–2050). Figures c and d, e and f, and g and h show similar trend and change information for built-up areas, croplands, and woody savannas respectively. (TIF) [file pone.0311816.s008.tif]

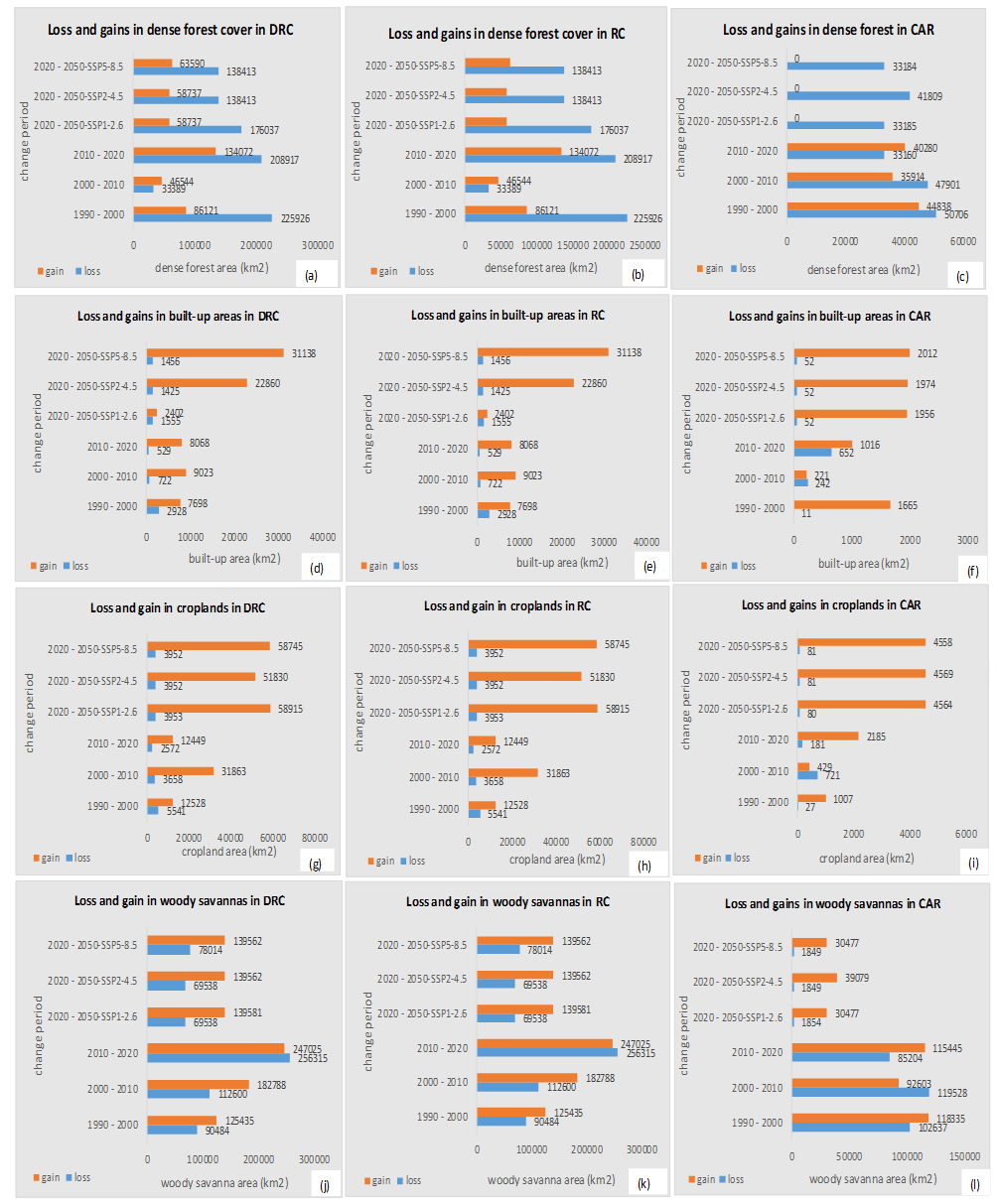

Supplement: S9 Fig — (TIF) [file pone.0311816.s009.tif]

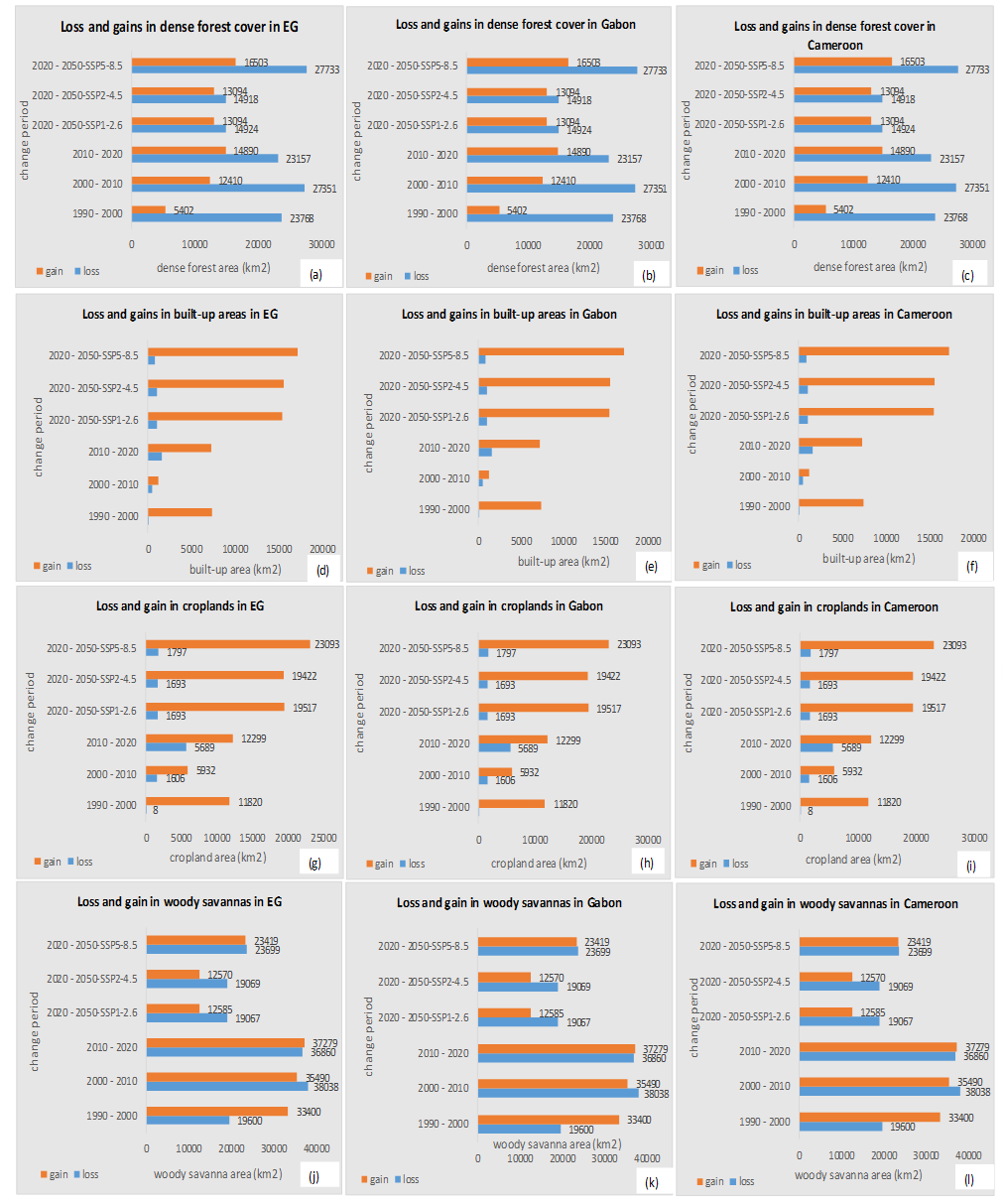

Supplement: S10 Fig — (TIF) [file pone.0311816.s010.tif]

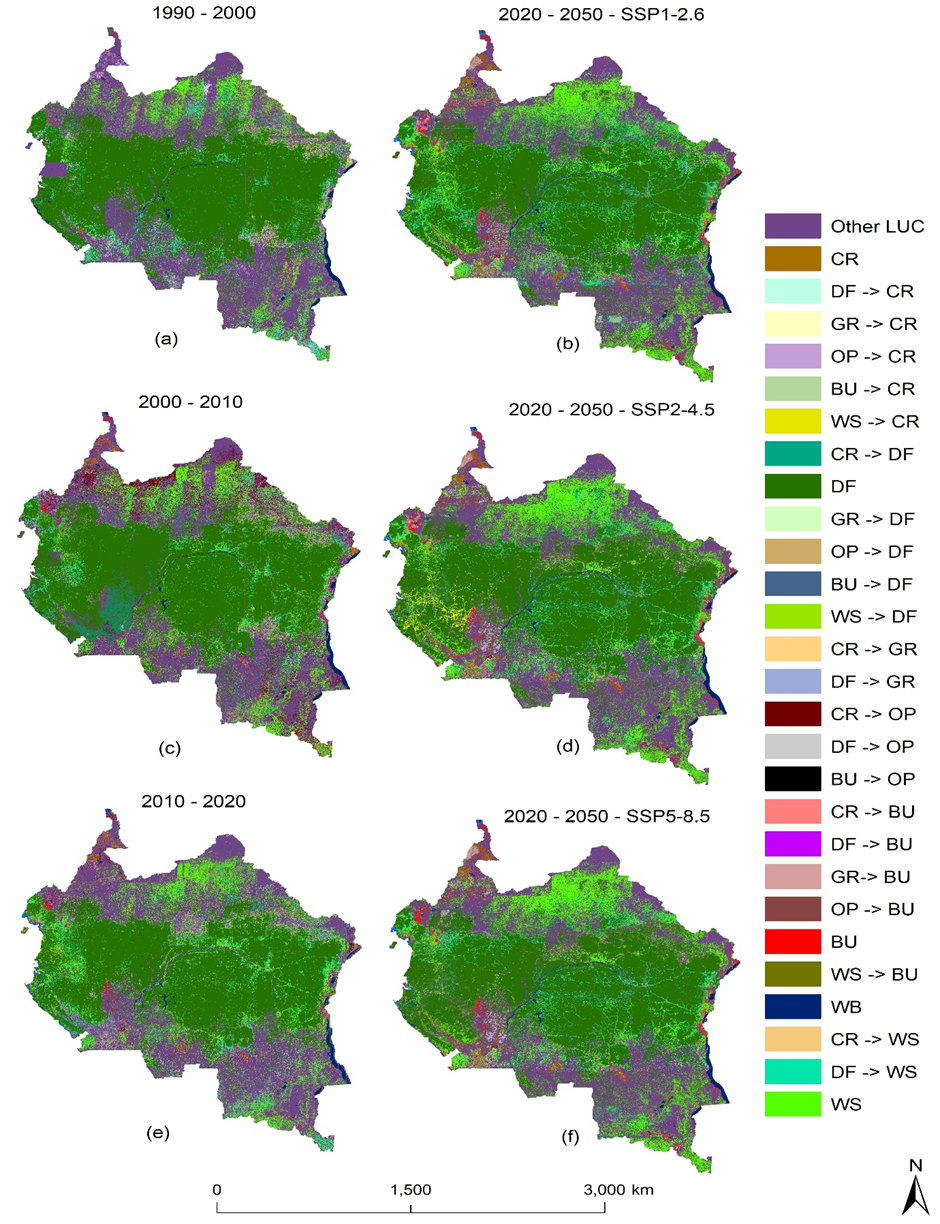

Supplement: S11 Fig — Map shows detected changes from one land cover class in time (T1) to another in time (T2). Changes are shown for the most important LULC variables that can help support policy planning. * CR = Croplands; DF = Dense forest; GR = Grassland savannas; OP = Open savannas/barelands; WB = Water bodies; WL = Wetlands; WS = Woody savannas; BU = Built-up; Other LUC = Other Land use and Land cover classes. Map generated with the RF Machine learning model, using the UUSGS Landsat 5 and 7 Collection 2 Level 2 data, freely available for public use with no data restrictions nor permissions required: https://www.usgs.gov/faqs/are-there-any-restrictions-use-or-redistribution-landsat-data. (TIF) [file pone.0311816.s011.tif]
