# Supplementary material for: Monitoring forest cover and land use change in the Congo Basin under IPCC climate change scenarios
Source: PLoS One. 2024 Dec 2;19(12):e0311816. doi: 10.1371/journal.pone.0311816 (PMC11611213; doi:10.1371/journal.pone.0311816)
Supplement: S1 Table — (PDF) [file pone.0311816.s012.pdf]

S1 Table

Name	Description	References
Croplands	Lands with at least 60% of the area cultivated as croplands.	Friedl and Sulla-Menashe [1]
Dense forest	Areas of land with trees ≥ 5m in height,[3,4] and a canopy cover > 60%.	Friedl and Sulla-Menashe [1]
Grasslands/savannas	Land use areas dominated by herbaceous vegetation or grasslands	Friedl and Sulla-Menashe [1]
Open savannas/barelands	Land use areas that are non-vegetated or contain less than 10% vegetation.	Friedl and Sulla-Menashe [1]
Built-up areas	Man-made land surfaces associated with built-up lands such as commercial and residential infrastructures, and roads.	Friedl and Sulla-Menashe [1]
Water bodies	inland areas covered with atleast 60% permanent water, and not obscured by objects above the surface such as buildings, tree canopies, and bridges.	Friedl and Sulla-Menashe [1], and Potapov et al. [4]
Wetlands	Vegetated and non-vegetated lands inundated with between 30-60% water and usually forming swampy or peatlands .	Friedl and Sulla-Menashe [1]
Woody savannas	Areas of land with a canopy cover of between 30 - 60%.	Friedl and Sulla-Menashe [1]
