# Supplementary material for: Monitoring forest cover and land use change in the Congo Basin under IPCC climate change scenarios
Source: PLoS One. 2024 Dec 2;19(12):e0311816. doi: 10.1371/journal.pone.0311816 (PMC11611213; doi:10.1371/journal.pone.0311816)
Supplement: S2 Table — (PDF) [file pone.0311816.s013.pdf]

S2 Table

Data category	Data type	Source	Data years/ projections	Description
Climate-related	Annual temperatures (maximum and minimum temperatures), and annual precipitations	IPCC-AR6 ensemble climate projections of the CMIP6 project [5,6]	1990-2020, 2050	Gridded climatic data for the Congo Basin, obtained from the IPCC-AR6 ensemble climate projections of the CMIP6 project.
	Forest fires	MODIS fire products: (https://modis.gsfc.nasa.gov/data/dataprod/mod14.php	2000-2020	Daily fire data recorded at 1km spatial resolution
Socioeconomic	Industrial selective logging, small scale clearing for agriculture, large scale agro-industrial clearing, mining, roads, settlements	Tyukavina et al.[7]	2000-2018	Mapped socioeconomic data for the Congo Basin. Data available only for current conditions. Roads and settlement data calculated as distance to built-up areas, using the Euclidean distance tool in ArcGIS.
Biophysical	Landscape topography (slope Elevation)	http://srtm.usgs.gov/index.php	Not applicable	Digital elevation models were acquired for the Congo Basin, from which slope data was extracted.
Demographic data	Population density	https://dataguru.lu.se/app#worldpop	2010-2020, 2050	Current and projected population density data acquired from the Veiko Lehsten climate and population projection data, and modeled under three three shared socioeconomic pathways (SSP1, SSP2 and SSP3), through the Coupled Model Intercomparison Project 6 (CMIP6) of the IPCC, at a 1 km spatial resolution
