# Supplementary material for: Monitoring forest cover and land use change in the Congo Basin under IPCC climate change scenarios
Source: PLoS One. 2024 Dec 2;19(12):e0311816. doi: 10.1371/journal.pone.0311816 (PMC11611213; doi:10.1371/journal.pone.0311816)
Supplement: S3 Table — Table was designed, following the approach used in Gibson et al. [91], and with drivers of change selected from S4–S11 Tables. (PDF) [file pone.0311816.s014.pdf]

S3 Table

LULC transitions or change	Transition sub-model and label	Description	Drivers of change
Dense forest to Croplands; Dense forest to Built-up areas; Dense forest to Open savannas/Barelands; Dense forest to Grassland savannas; Dense forest to Wetlands; Dense forest to Water bodies; and Dense forest to Woody savannas	Dense forest loss or deforestation and forest degradation (DD)	Conversion or change from forest cover to other LULC types	Wildland fires; Population density; Maximum temperatures; Minimum Temperatures; Logging and forest clearing; and Distance to built-up areas
Croplands to Forest; Open savannas/Barelands to Forest; Grasslands savannas to Forest; Wetlands to Forest; Water bodies to Forest; and Woody savannas to Forest	Dense forest gain or afforestation and forest enrichment (AE)	Conversion or change from other land use types to forest	Precipitation; Slope;
Dense forest to Croplands; Open savannas/Barelands to Croplands; Grassland savannas to croplands; Built-up areas to Croplands Wetlands to croplands; Water bodies to croplands; and Woody savannas to Croplands	Croplands Intensification (CI)	Conversion or change from other land use types to croplands	Population density
Croplands to Dense forest; Croplands to open savannas/Barelands; Croplands to Grassland savannas; Croplands to Built-up areas; Croplands to Water bodies; Croplands to Wetlands; and Croplands to Woody savannas	Croplands Abandonment (CA)	Conversion or change from croplands to other land use types	Population density, and Maximum temperatures
Dense forest to Built-up areas; Croplands to Built-up areas; Open savannas/Barelands to Built-up areas; Grassland savannas to Built-up areas; Water bodies to Built-up areas; Wetlands to Built-up areas; and Woody savannas to Built-up areas	Built-up areas Intensification (BUI)	Conversion or change from other LULC types to built-up areas	Population density; and distance to built-up areas
Built-up to Dense forest; Built-up areas to Grassland savannas; Built-up areas to Open savannas/Barelands; Built-up areas to Water bodies; Built-up areas to Wetlands; and Built-up areas to Woody savannas	Built-up areas Abandonment (BUA)	Conversion or change from built-up areas to other LULC types	Distance to built-up areas; and slope
Woody savannas to Dense forest; Woody savannas to Croplands; Woody savannas to Built-up areas; Woody savannas to open savannas/Barelands; Woody savannas to Grassland savannas; Woody savannas to Water bodies; and Woody savannas to Wetlands	Woody savannah Area loss (WAL)	Conversion or change from woody savannas areas to other LULC types	Wildland fires, maximum temperatures, population density, and logging and forest clearing
Dense forest to Woody savannas; Croplands to Woody savannas;	Woody savannah Area Increase (WAI)	Conversion or change from other LULC	Precipitation; maximum and

Built-up to Woody savannas; Open savannas/Barelands to Woody savannas; Grassland savannas to Woody savannas; Water bodies to Woody savannas; and Wetlands to Woody savannas		types to woody savannas	minimum temperatures; slope, and logging and forest clearing
Open savannas/Barelands to Woody savannas; Open savannas/Barelands to Built-up areas; Open savannas/Barelands to Grassland savannas; Open savannas/Barelands to Water bodies; Open savannas/Barelands to Wetlands to Woody savannas; Open savannas/Barelands to Croplands; and Open savannas/Barelands to Dense forest	Open savannas/Barelands Depletion (OBD)	Conversion or change from other LULC types to open savannas/barelands	Population density, distance to built-up areas, precipitation, minimum temperatures, elevation and slope
Woody savannas to Open savannas/Barelands; Built-up areas to Open savannas/Barelands; Dense forest to Open savannas/Barelands; Croplands to Open savannas/Barelands; Water bodies to Open savannas/Barelands; Wetlands to Open savannas/Barelands; and Grassland savannas to Open savannas/Barelands	Open savannas/Barelands Area Increase (OBAI)	Conversion or change from open savannas/barelands to other LULC types	Logging and forest clearing, distance to built-up areas, population density, wildland fires, and elevation
Woody savannas to Grassland savannas; Built-up areas to Grassland savannas; Dense forest to Grassland savannas; Croplands to Grassland savannas; Water bodies to Grassland savannas; Wetlands to Grassland savannas; and Open savannas/Barelands to Grassland savannas	Grassland savannah Area Increase (GSAI)	Conversion or change from other LULC types to grassland savannas	Elevation, slope, maximum and minimum temperatures, distance to built-up areas, population density, and logging and forest clearing
Grassland savannas to Open savannas/Barelands; Grassland savannas to Croplands; Grassland savannas to Built-up areas; Grassland savannas to Dense forest; Grassland savannas to Woody savannas; Grassland savannas to Water bodies; and Grassland savannas to Wetlands	Grassland savannah Area Decline (GSAD)	Conversion or change from grassland savannas to other LULC types	Population density, wildland fires, distance to built-up areas, and maximum temperatures
Water bodies to Open savannas/Barelands; Water bodies to Croplands; Water bodies to Built-up areas; Water bodies to Dense forest; Water bodies to Woody savannas; Water bodies to Grassland savannas; and Water bodies to Wetlands	Water body Loss (WL)	Conversion or change from water bodies to other LULC types	Maximum temperatures; and slope
Open savannas/Barelands to Water bodies; Croplands to Water bodies; Built-up areas to Water bodies; Dense forest to Water	Water body Increase (WI)	Conversion or change from other LULC types to water bodies	Precipitation; and logging and forest clearing

bodies; Woody savannas to Water bodies; Grassland savannas to Water bodies; and Wetlands to Water bodies			
Open savannas/Barelands to Wetlands; Croplands to Wetlands; Built-up to Wetlands; Dense forest to Wetlands; Woody savannas to Wetlands; Grassland savannas to Wetlands; and Water bodies to Wetlands	Wetland Increase (WI)	Conversion or change from other LULC types to wetlands	Precipitation
Wetlands to Open savannas/Barelands; Wetlands to Croplands; Wetlands to Built-up areas; Wetlands to Dense forest; Wetlands to Woody savannas; Wetlands to Grassland savannas; and Wetlands to Water bodies	Wetlands Loss (WetL)	Conversion or change from wetlands to other LULC types	Maximum temperatures, wildland fires and slope
