# Supplementary material for: Monitoring forest cover and land use change in the Congo Basin under IPCC climate change scenarios
Source: PLoS One. 2024 Dec 2;19(12):e0311816. doi: 10.1371/journal.pone.0311816 (PMC11611213; doi:10.1371/journal.pone.0311816)
Supplement: S4 Table — (PDF) [file pone.0311816.s015.pdf]

S4 Table

**Target variables**	**Built-up area Intensification**			**Built-up area Abandonment**		
**Predictor variables**	**R^2**	**Adjusted R^2**	**p-value**	**R^2**	**Adjusted R^2**	**p-value**
Logging and forest clearing	0.03	-0.01	0.4088	0.003	-0.001	0.4088
Distance to built-up areas	0.4	0.4	2.2e-16	0.4	0.4	2.2e-16
Elevation	0.08	-0.03	0.6679	0.009	-0.004	0.6679
Slope	0.07	0.03	0.2064	0.7	0.6	0.00206
Wildland fires	0.02	-0.02	0.5024	0.002	-0.002	0.5024
Population density	0.3	0.2	0.0006	0.002	-0.003	0.6104
precipitation	0.05	-0.04	0.9165	0.005	-0.004	0.9165
Maximum temperature	0.01	0.01	0.1088	0.01	0.007	0.1088
Minimum temperature	0.02	0.01	0.05392	0.02	0.01	0.05392
