# Supplementary material for: Monitoring forest cover and land use change in the Congo Basin under IPCC climate change scenarios
Source: PLoS One. 2024 Dec 2;19(12):e0311816. doi: 10.1371/journal.pone.0311816 (PMC11611213; doi:10.1371/journal.pone.0311816)
Supplement: S5 Table — (PDF) [file pone.0311816.s016.pdf]

S5 Table

**Target variables**	**Croplands Intensification**			**Croplands Abandonment**		
**Predictor variables**	**R^2**	**Adjusted R^2**	**p-value**	**R^2**	**Adjusted R^2**	**p-value**
Logging and forest clearing	0.001	-0.001	0.5492	0.002	-0.001	0.4385
Distance to built-up areas	0.09	0.06	0.0601	0.004	-0.003	0.9704
Elevation	0.05	0.02	0.182	0.05	0.02	0.1768
Slope	0.03	0.09	0.2451	0.007	-0.003	0.8739
Wildland fires	0.04	0.02	0.1718	0.36	0.31	0.00714
Population density	0.2	0.2	0.04246	0.002	-0.002	0.7663
precipitation	0.005	-0.002	0.6663	0.02	-0.02	0.7651
Maximum temperature	0.09	-0.02	0.5343	0.66	0.63	0.00119
Minimum temperature	0.08	-0.02	0.561	0.001	-0.001	0.489
