# Supplementary material for: Monitoring forest cover and land use change in the Congo Basin under IPCC climate change scenarios
Source: PLoS One. 2024 Dec 2;19(12):e0311816. doi: 10.1371/journal.pone.0311816 (PMC11611213; doi:10.1371/journal.pone.0311816)
Supplement: S6 Table — (PDF) [file pone.0311816.s017.pdf]

S6 Table

Target variables	Afforestation and forest enrichment (dense forest gain)			Deforestation and forest degradation (dense forest loss)		
**Predictor variables**	**R^2**	**Adjusted R^2**	**p-value**	**R^2**	**Adjusted R^2**	**p-value**
Logging and forest clearing	0.01	0.01	1.07e-14	0.67	0.65	0.00071
Distance to built-up areas	0.12	0.12	2.2e-16	0.26	0.26	2.2e-16
Elevation	0.16	0.16	2.2e-16	0.2	0.2	2.2e-16
Slope	0.69	0.67	6.86e-08	0.14	0.13	2.2e-16
Wildland fires	0.08	0.08	2.2e-16	0.42	0.42	2.2e-16
Population density	0.03	0.03	2.2e-16	0.21	0.19	0.00139
precipitation	0.59	0.58	0.00091	0.02	0.02	0.001266
Maximum temperature	0.05	0.03	0.00118	0.66	0.22	0.00922
Minimum temperature	0.07	0.06	0.053	0.22	0.64	0.00077
