# Supplementary material for: Monitoring forest cover and land use change in the Congo Basin under IPCC climate change scenarios
Source: PLoS One. 2024 Dec 2;19(12):e0311816. doi: 10.1371/journal.pone.0311816 (PMC11611213; doi:10.1371/journal.pone.0311816)
Supplement: S7 Table — (PDF) [file pone.0311816.s018.pdf]

S7 Table

Target variables	Grassland savanna area increase			Grassland savanna area decline		
**Predictor variables**	**R^2**	**Adjusted R^2**	**p-value**	**R^2**	**Adjusted R^2**	**p-value**
Logging and forest clearing	0.16	0.15	0.04997	0.001	-0.007	0.6942
Distance to built-up areas	0.45	0.36	0.02351	0.16	0.11	0.01698
Elevation	0.48	0.41	0.01855	0.07	-0.08	0.7743
Slope	0.96	0.84	0.00297	0.04	-0.04	0.5003
Wildland fires	0.11	0.1	0.00038	0.44	0.36	0.0018
Population density	0.18	0.11	0.1546	0.24	0.2	0.00962
precipitation	0.1	0.1	0.01945	0.07	-0.02	0.3794
Maximum temperature	0.19	0.19	4.43e-05	0.22	0.21	0.01117
Minimum temperature	0.17	0.17	0.000171	0.12	0.12	8.5e-05
