# Supplementary material for: Monitoring forest cover and land use change in the Congo Basin under IPCC climate change scenarios
Source: PLoS One. 2024 Dec 2;19(12):e0311816. doi: 10.1371/journal.pone.0311816 (PMC11611213; doi:10.1371/journal.pone.0311816)
Supplement: S8 Table — (PDF) [file pone.0311816.s019.pdf]

S8 Table

Target variables	Open savannas/Barelands Area Increase			Open savannas/Barelands Area Depletion		
**Predictor variables**	**R^2**	**Adjusted R^2**	**p-value**	**R^2**	**Adjusted R^2**	**p-value**
Logging and forest clearing	0.31	0.29	0.00021	0.003	0.002	0.00019
Distance to built-up areas	0.3	0.3	0.01367	0.18	0.13	0.01367
Elevation	0.28	0.25	0.00040	0.28	0.25	0.00040
Slope	0.02	0.02	0.00059	0.26	0.24	0.00062
Wildland fires	0.17	0.17	0.03361	0.002	-0.002	0.3361
Population density	0.41	0.41	0.04632	0.21	0.21	0.04632
precipitation	0.01	0.01	6.4e-12	0.19	0.19	6.45e-12
Maximum temperature	0.00	0.00	0.9	0.002	-0.002	0.9231
Minimum temperature	0.02	0.02	2.2e-16	0.15	0.15	2.2e-16
