# Supplementary material for: Monitoring forest cover and land use change in the Congo Basin under IPCC climate change scenarios
Source: PLoS One. 2024 Dec 2;19(12):e0311816. doi: 10.1371/journal.pone.0311816 (PMC11611213; doi:10.1371/journal.pone.0311816)
Supplement: S9 Table — (PDF) [file pone.0311816.s020.pdf]

S9 Table

**Target variables**	**Water body increase**			**Water body loss**		
**Predictor variables**	**R^2**	**Adjusted R^2**	**p-value**	**R^2**	**Adjusted R^2**	**p-value**
Logging and forest clearing	0.47	0.37	0.02723	0.05	0.04	0.02723
Distance to built-up areas	0.002	-0.009	0.967	0.002	-0.009	0.967
Elevation	0.004	-0.005	0.5026	0.004	-0.005	0.5026
Slope	0.08	0.08	0.00196	0.91	0.82	0.0023
Wildland fires	0.04	0.03	0.03466	0.04	0.03	0.03466
Population density	0.007	-0.009	0.5276	0.007	-0.009	0.5276
precipitation	0.44	0.43	0.00832	0.004	-0.009	0.8527
Maximum temperature	0.18	0.17	0.004601	0.7	0.7	0.0056
Minimum temperature	0.16	0.11	0.1949	0.02	0.01	0.29
