# Supplementary material for: Monitoring forest cover and land use change in the Congo Basin under IPCC climate change scenarios
Source: PLoS One. 2024 Dec 2;19(12):e0311816. doi: 10.1371/journal.pone.0311816 (PMC11611213; doi:10.1371/journal.pone.0311816)
Supplement: S10 Table — (PDF) [file pone.0311816.s021.pdf]

S10 Table

Target variables	Wetland gain			Wetland loss		
**Predictor variables**	**R^2**	**Adjusted R^2**	**p-value**	**R^2**	**Adjusted R^2**	**p-value**
Logging and forest clearing	0.02	0.004	0.2546	0.008	-0.004	0.4177
Distance to built-up areas	0.02	0.008	0.2174	0.03	0.02	0.1319
Elevation	0.05	0.04	0.05686	0.11	0.1	0.00249
Slope	0.05	0.04	0.07496	0.24	0.21	0.01671
Wildland fires	0.02	0.02	3.9e-06	0.29	0.28	2.6e-07
Population density	0.004	-0.02	0.6649	0.01	-0.001	0.3426
precipitation	0.94	0.81	0.01074	0.11	0.11	0.00208
Maximum temperature	0.002	-0.01	0.9149	0.2	0.2	0.00208
Minimum temperature	0.07	0.06	0.02538	0.13	0.12	0.00122
