# Supplementary material for: Monitoring forest cover and land use change in the Congo Basin under IPCC climate change scenarios
Source: PLoS One. 2024 Dec 2;19(12):e0311816. doi: 10.1371/journal.pone.0311816 (PMC11611213; doi:10.1371/journal.pone.0311816)
Supplement: S11 Table — (PDF) [file pone.0311816.s022.pdf]

S11 Table

Target variables	Woody savanna gain			Woody savanna loss		
**Predictor variables**	**R^2**	**Adjusted R^2**	**p-value**	**R^2**	**Adjusted R^2**	**p-value**
Logging and forest clearing	0.83	0.79	5.4e-05	0.35	0.31	0.008273
Distance to built-up areas	0.01	0.01	0.0661	0.04	0.04	0.003014
Elevation	0.12	0.11	1.2e-06	0.01	0.01	7.28e-08
Slope	0.31	0.26	0.01368	0.03	0.02	0.01529
Wildland fires	0.14	0.14	6.9e-08	0.23	0.22	1.25e-11
Population density	0.002	-0.002	0.5017	0.77	0.76	0.002195
precipitation	0.93	0.88	2.0e-05	0.15	0.11	0.08551
Maximum temperature	0.28	0.23	0.0183	0.41	0.36	0.004641
Minimum temperature	0.22	0.21	6.3e-11	0.13	0.13	2.84e-07
