# Supplementary material for: Monitoring forest cover and land use change in the Congo Basin under IPCC climate change scenarios
Source: PLoS One. 2024 Dec 2;19(12):e0311816. doi: 10.1371/journal.pone.0311816 (PMC11611213; doi:10.1371/journal.pone.0311816)
Supplement: S12 Table — Table shows Correlation strengths between our mapped LULC data for the year 2010, and datasets predicted for the year 2010 by the TerrSet Idrissi Land Change Modeler. b; Accuracy validation for our predicted LULC datasets for the year 2020. Table shows Correlation strengths between our mapped LULC data for the year 2020, and datasets predicted for the year 2020 by the TerrSet Idrissi Land Change Modeler. (PDF) [file pone.0311816.s023.pdf]

S12a Table

LULC classes	Croplands predicted 2010	Dense forest predicted 2010	Grassland savanna predicted 2010	Open savannas/ barelands predicted 2010	Built-up area predicted 2010	Water bodies predicted 2010	Wetlands predicted 2010	Woody savanna predicted 2010
Croplands	0.7							
Dense forest		0.9						
Grassland savanna			0.6					
Open savannas/ barelands				0.8				
Built-up areas					0.8			
Water bodies						0.8		
Wetlands							0.8	
Woody savanna								0.7

Overall correlation strength = 0.8

S12b Table

LULC classes	Croplands predicted 2020	Dense forest predicted 2020	Grassland savanna predicted 2020	Open savannas/ barelands predicted 2020	Built-up area predicted 2020	Water bodies predicted 2020	Wetlands predicted 2020	Woody savanna predicted 2020
Croplands	0.8							
Dense forest		0.9						
Grassland savanna			0.5					
Open savannas/ barelands				0.8				
Built-up areas					0.8			
Water bodies						0.9		
Wetlands							0.8	
Woody savanna								0.7

Overall correlation strength = 0.8
