# Supplementary material for: Monitoring forest cover and land use change in the Congo Basin under IPCC climate change scenarios
Source: PLoS One. 2024 Dec 2;19(12):e0311816. doi: 10.1371/journal.pone.0311816 (PMC11611213; doi:10.1371/journal.pone.0311816)
Supplement: S13 Table — b; Quantified decadal changes in land cover patterns in CAR, between 1990–2020. (PDF) [file pone.0311816.s024.pdf]

S13a Table

	1990		2000		2010		2020		2050					
									SSP1-2.6		SSP2-4.5		SSP5-8.5	
LULC class	Area (km2)	% Area	Area (km2)	% Area	Area (km2)	% Area	Area (km2)	% Area	Area (km2)	% Area	Area (km2)	% Area	Area (km2)	% Area
croplands	28	0	1008.5	0.2	717.3	0.1	2721.5	0.4	7204.3	1.2	7209.7	1.2	7198.5	1.2
dense forest	138947.1	22.2	132402.5	21.1	120399.7	19.2	127521.6	20.4	85670.7	13.9	85671.2	13.7	85671.2	13.9
grassland/savannas	3697.5	0.6	12930.1	2.1	8323.3	1.3	11215.7	1.8	12902.5	2.1	12950.4	2.1	12902.5	2.1
open savannas/barelands	277273.3	44.2	252308	40.3	299309	47.8	256369.9	41	252769.5	41	252713.7	40.4	252713.7	40.9
built-up areas	23.3	0	1677.6	0.3	1657	0.3	2020.2	0.3	3924.5	0.6	3942.6	0.6	3980.1	0.6
water bodies	1055.5	0.2	2576.6	0.4	1169.5	0.2	1046	0.2	1029.7	0.2	1030.5	0.2	1029.7	0.2
wetlands	83.5	0	2190.3	0.3	360.9	0.1	636.9	0.1	627.1	0.1	627	0.1	627	0.1
woody savannas	205782.8	32.8	221284.2	35.3	194285.4	31	224512.5	35.9	253006.8	41	261614.7	41.8	253012.2	41
Total	626891.1	100	626377.8	100	626222	100	626044.3	100	617135.1	100	625759.9	100	617135.1	100

S13b Table

	1990-2000		2000-2010		2010-2020		2020-2050					
							SSP1-2.6		SSP2-4.5		SSP5-8.5	
LULC classes	Area (km2)	% Area	Area (km2)	% Area	Area (km2)	% Area	Area (km2)	% Area	Area (km2)	% Area	Area (km2)	% Area
croplands	980.4	0.2	-291.1	0	2004.2	0.3	4482.8	0.8	4488.2	0.8	4477	0.8
dense forest	-6544.7	-1	-12002.8	-1.9	7121.9	1.1	-41850.9	-6.5	-41850.4	-6.7	-41850.4	-6.5
grassland/savannas	9232.6	1.5	-4606.8	-0.7	2892.4	0.5	1686.8	0.3	1734.7	0.3	1686.8	0.3
open savannas/barelands	-24965.3	-3.9	47001	7.5	-42939.1	-6.8	-3600.4	0.0	-3656.2	-0.6	-3656.2	-0.1
built-up areas	1654.3	0.3	-20.6	0	363.2	0.1	1904.3	0.3	1922.4	0.3	1959.9	0.3
water bodies	1521.1	0.2	-1407.1	-0.2	-123.5	0	-16.3	0.0	-15.5	0.0	-16.3	0.0
wetlands	2106.9	0.3	-1829.4	-0.3	276	0	-9.8	0.0	-9.9	0.0	-9.9	0.0
woody savannas	15501.4	2.5	-26998.8	-4.3	30227.1	4.8	28494.3	5.1	37102.2	5.9	28499.7	5.1
