# Supplementary material for: Monitoring forest cover and land use change in the Congo Basin under IPCC climate change scenarios
Source: PLoS One. 2024 Dec 2;19(12):e0311816. doi: 10.1371/journal.pone.0311816 (PMC11611213; doi:10.1371/journal.pone.0311816)
Supplement: S14 Table — b; Quantified decadal changes in land cover patterns in the Republic of Congo, between 1990–2020. (PDF) [file pone.0311816.s025.pdf]

S14a Table

	1990		2000		2010		2020		2050					
									SSP1-2.6		SSP2-4.5		SSP5-8.5	
**LULC class**	**Area (km2)**	**% Area**	**Area (km2)**	**% Area**	**Area (km2)**	**% Area**	**Area (km2)**	**% Area**	Area (km2)	% Area	Area (km2)	% Area	Area (km2)	% Area
croplands	1284.9	0.4	3917.7	1.1	821	0.2	10170.9	3	25675	7.5	28147.8	8.7	28147.8	8.7
dense forest	249757.2	73.2	224498.4	65.8	224974	65.9	215203.1	63	215060.2	63	193855.3	59.8	193855.3	59.8
grassland/savannas	422.5	0.1	618.8	0.2	394	0.1	3519.6	1	3518.5	1	3518.5	1.1	3518.5	1.1
open savannas/barelands	81643.3	23.9	91908.8	26.9	93961.5	27.5	80364.2	23.5	62917.2	18.4	62914.6	19.4	62914.6	19.4
built-up areas	33.1	0	800.8	0.2	2747.5	0.8	4857.6	1.4	9539.9	2.8	11048.9	3.4	11048.9	3.4
water bodies	3258.8	1	4264.7	1.2	3437.4	1	4111.7	1.2	3879	1.1	3878.7	1.2	3878.7	1.2
wetlands	33.8	0	60.1	0	822.1	0.2	1138.9	0.3	1061.9	0.3	1062	0.3	1062	0.3
woody savannas	4778.4	1.4	15329.3	4.5	14395.1	4.2	22029	6.5	19527.7	5.7	19527.3	6	19527.3	6
Total	341211.9	100	341398.7	100	341552.6	100	341395	100	341179.5	100	323953.1	100	323953.1	100

S14b Table

	1990-2000		2000-2010		2010-2020		2020-2050					
							SSP1-2.6		SSP2-4.5		SSP5-8.5	
**LULC classes**	**Area (km2)**	**% Area**	**Area (km2)**	**% Area**	**Area (km2)**	**% Area**	**Area (km2)**	**% Area**	**Area (km2)**	**% Area**	**Area (km2)**	**% Area**
croplands	2632.8	0.8	-3096.6	-0.9	9349.9	2.7	21757.3	6.4	24230.1	7.6	24230.1	7.6
dense forest	-25258.8	-7.4	475.6	0.1	-9770.9	-2.8	-9438.2	-2.8	-30643.1	-6.0	-30643.1	-6.0
grassland/savannas	196.3	0.1	-224.8	-0.1	3125.5	0.9	2899.7	0.8	2899.7	0.9	2899.7	0.9
open savannas/barelands	10265.5	3	2052.7	0.6	-13597.3	-4	-28991.6	-8.5	-28994.2	-7.5	-28994.2	-7.5
built-up areas	767.7	0.2	1946.7	0.6	2110.1	0.6	8739.1	2.6	10248.1	3.2	10248.1	3.2
water bodies	1006	0.3	-827.3	-0.2	674.3	0.2	-385.7	-0.1	-386.0	0.0	-386.0	0.0
wetlands	26.3	0.0	762.0	0.2	316.8	0.1	1001.8	0.3	1001.9	0.3	1001.9	0.3
woody savannas	10550.9	3.1	-934.3	-0.3	7634	2.2	4198.4	1.2	4198.0	1.5	4198.0	1.5
