# Supplementary material for: Monitoring forest cover and land use change in the Congo Basin under IPCC climate change scenarios
Source: PLoS One. 2024 Dec 2;19(12):e0311816. doi: 10.1371/journal.pone.0311816 (PMC11611213; doi:10.1371/journal.pone.0311816)
Supplement: S15 Table — b; Quantified decadal changes in land cover patterns in the DRC, between 1990–2020. (PDF) [file pone.0311816.s026.pdf]

S15a Table

	**1990.0**		**2000**		**2010**		**2020**		**2050**					
									**SSP1-2.6**		**SSP2-4.5**		**SSP5-8.5**	
**LULC class**	**Area (km2)**	**% Area**	**Area (km2)**	**% Area**	**Area (km2)**	**% Area**	**Area (km2)**	**% Area**	**Area (km2)**	**% Area**	**Area (km2)**	**% Area**	**Area (km2)**	**% Area**
croplands	469.3	0	14581.6	0.6	17597.1	0.7	45544.8	1.9	100303	4.2	93227.7	4	100141.7	4.3
dense forest	1448210	60.6	1307375.7	54.7	1302441	54.5	1228411.1	51.4	1106579	46.5	1103029.5	47.2	1107882.2	47.4
grassland/savannas	16833.5	0.7	34191.9	1.4	25847.4	1.1	33424.2	1.4	45150.4	1.9	38094.7	1.6	38094.7	1.6
open savannas/ barelands	668915.2	28.0	714160.5	29.9	689246.5	28.8	690630.4	28.9	646924.2	27.2	655715.8	28.1	645227.8	27.6
built-up areas	324	0	8022.1	0.3	10380	0.4	20917.9	0.9	43347.3	1.8	42218.5	1.8	50466	2.2
water bodies	41992.5	1.8	46023.7	1.9	43404.1	1.8	43787.5	1.8	43044.9	1.8	43122.6	1.8	43067.1	1.8
wetlands	451.6	0	2116.3	0.1	2149.3	0.1	4513.8	0.2	4375.1	0.2	4408.6	0.2	4398	0.2
woody savannas	212233.1	8.9	261746.7	11	299426.9	12.5	323115.1	13.5	391985.5	16.5	357764.1	15.3	348304	14.9
Total	2389429.2	100	2388218.5	100	2390492.1	100	2390344.8	100	2381710	100	2337581	100	2337581	100

S15b Table

	**1990-2000**		**2000-2010**		**2010-2020**		**2020-2050**					
							**SSP1-2.6**		**SSP2-4.5**		**SSP5-8.5**	
**LULC classes**	**Area (km2)**	**% Area**	**Area (km2)**	**% Area**	**Area (km2)**	**% Area**	**Area (km2)**	**% Area**	**Area (km2)**	**% Area**	**Area (km2)**	**% Area**
croplands	14112.3	0.6	3015.5	0.1	27947.7	1.2	54758.5	2.3	47682.9	2.1	54596.9	2.4
dense forest	-140834.3	-5.9	-4934.7	-0.3	-74029.9	-3.1	-121831.7	-4.9	-125381.6	-4.2	-120528.9	-4.0
grassland/savannas	17358.4	0.7	-8344.5	-0.4	7576.9	0.3	11726.2	0.5	4670.5	0.2	4670.5	0.2
open savannas/barelands	45245.3	1.9	-24914	-1.1	1383.9	0.1	-43706.2	-1.7	-34914.6	-0.8	-45402.6	-1.3
built-up areas	7698.1	0.3	2357.9	0.1	10538	0.4	22429.4	0.9	21300.6	0.9	29548.1	1.3
water bodies	4031.2	0.2	-2619.6	-0.1	383.4	0	-742.6	0.0	-664.9	0.0	-720.4	0.0
wetlands	1664.7	0.1	32.9	0	2364.5	0.1	-138.7	0.0	-105.2	0.0	-115.8	0.0
woody savannas	49513.6	2.1	37680.3	1.6	23688.2	1	68870.4	3.0	34649	1.8	25188.9	1.4
