# Supplementary material for: Monitoring forest cover and land use change in the Congo Basin under IPCC climate change scenarios
Source: PLoS One. 2024 Dec 2;19(12):e0311816. doi: 10.1371/journal.pone.0311816 (PMC11611213; doi:10.1371/journal.pone.0311816)
Supplement: S16 Table — b; Quantified decadal changes in land cover patterns in EG, between 1990–2020. (PDF) [file pone.0311816.s027.pdf]

S16a Table

	1990		2000		2010		2020		2050					
									**SSP1-2.6**		**SSP2-4.5**		**SSP5-8.5**	
**LULC class**	**Area (km2)**	**% Area**	**Area (km2)**	**% Area**	**Area (km2)**	**% Area**	**Area (km2)**	**% Area**	Area (km2)	% Area	Area (km2)	% Area	Area (km2)	% Area
croplands	13.1	0	996.9	3.7	3.2	0	185.3	0.7	233.4	0.9	233.4	0.9	233.4	0.9
dense forest	26739.6	98.7	22850.8	84.4	19669.4	73.2	24490	91.1	24896.1	92.6	24896.1	92.6	24896.1	92.6
grassland/savannas	1.9	0	42.1	0.2	58.9	0.2	24	0.1	5.7	0	5.7	0	5.7	0
open savannas/barelands	126.5	0.5	245.2	0.9	1758.6	6.5	149.6	0.6	135.5	0.5	135.5	0.5	135.5	0.5
built-up areas	1.6	0	244.9	0.9	270.9	1	565.1	2.1	836.5	3.1	836.5	3.1	836.5	3.1
water bodies	169.1	0.6	298.1	1.1	229.8	0.9	259.7	1.0	256.7	1	256.7	1	256.7	1
wetlands	0.2	0	1.9	0	437.2	1.6	1.2	0.0	1.2	0	1.2	0	1.2	0
woody savannas	30.1	0.1	2404.6	8.9	4456.2	16.6	1202.1	4.5	512	1.9	512	1.9	512	1.9
Total	27082	100	27084.6	100	26884.2	100	26877.1	100	26877.1	100	26877.1	100	26877.1	100

S16b Table

	1990-2000		2000-2010		2010-2020		2020-2050					
							**SSP1-2.6**		**SSP2-4.5**		**SSP5-8.5**	
**LULC classes**	**Area (km2)**	**% Area**	**Area (km2)**	**% Area**	**Area (km2)**	**% Area**	**Area (km2)**	**% Area**	**Area (km2)**	**% Area**	**Area (km2)**	**% Area**
croplands	983.8	3.6	-993.7	-3.7	182.1	0.7	48.1	0.2	48.1	0.2	48.1	0.2
dense forest	-3888.8	-14.4	-3181.4	-11.2	4820.6	18	406.1	1.5	406.1	1.5	406.1	1.5
grassland/savannas	40.2	0.1	16.8	0.1	-34.9	-0.1	-18.3	-0.1	-18.3	-0.1	-18.3	-0.1
open savannas/barelands	118.7	0.4	1513.4	5.6	-1609	-6.0	-14.1	-0.1	-14.1	-0.1	-14.1	-0.1
built-up areas	243.3	0.9	26	0.1	294.2	1.1	271.4	1.0	271.4	1.0	271.4	1.0
water bodies	129	0.5	-68.3	-0.2	29.9	0.1	-3.0	0.0	-3.0	0.0	-3.0	0.0
wetlands	1.7	0	435.2	1.6	-435.9	-1.6	0.0	0.0	0.0	0.0	0.0	0.0
woody savannas	2374.6	8.8	2051.6	7.7	-3254.1	-12.1	-690.1	-2.6	-690.1	-2.6	-690.1	-2.6
