# Supplementary material for: Monitoring forest cover and land use change in the Congo Basin under IPCC climate change scenarios
Source: PLoS One. 2024 Dec 2;19(12):e0311816. doi: 10.1371/journal.pone.0311816 (PMC11611213; doi:10.1371/journal.pone.0311816)
Supplement: S17 Table — b. Quantified decadal changes in land cover patterns in Gabon, between 1990–2020. (PDF) [file pone.0311816.s028.pdf]

S17a Table

	1990		2000		2010		2020		2050					
									SSP1		SSP2		SSP5	
**LULC class**	**Area (km2)**	**% Area**	**Area (km2)**	**% Area**	**Area (km2)**	**% Area**	**Area (km2)**	**% Area**	Area (km2)	% Area	Area (km2)	% Area	Area (km2)	% Area
croplands	5.5	0	446.1	0.2	20.5	0	4763	1.8	8091.5	3.1	8091.5	3.1	8004.9	3.4
dense forest	242279.9	91.8	221234.6	83.8	205702.3	78	199165.8	75.1	189667.8	71.9	189667.8	71.9	189667.8	81.2
grassland/savannas	76.1	0	267.2	0.1	212.3	0.1	1031.3	0.4	1169.9	0.4	1169.9	0.4	1030.2	0.4
open savannas/barelands	17362.8	6.6	22842.6	8.7	20889.7	7.9	10612.8	4	7926.9	3.0	7926.9	3.0	7926.9	3.4
built-up areas	149.3	0.1	777.6	0.3	2826.3	1.1	3583.2	1.4	6189.1	2.3	6189.1	2.3	5923.5	2.5
water bodies	3134.1	1.2	4929.5	1.9	4379.1	1.7	5224.1	2	5152.1	2.0	5152.1	2.0	5187.9	2.2
wetlands	14.3	0	26.6	0	2110.7	0.8	2345.8	0.9	2054.1	0.8	2054.1	0.8	2054.1	0.9
woody savannas	847.4	0.3	13345.2	5.1	27425.3	10.4	38334.5	14.5	43486.2	16.5	43486.2	16.5	13879.4	5.9
Total	263869.4	100	263869.4	100	263566.2	100	265060.3	100	263737.4	100	263737.4	100	233674.6	100

S17b Table

	1990-2000		2000-2010		2010-2020		2020-2050					
							SSP1		SSP2		SSP5	
**LULC classes**	**Area (km2)**	**% Area**	**Area (km2)**	**% Area**	**Area (km2)**	**% Area**	**Area (km2)**	**% Area**	**Area (km2)**	**% Area**	**Area (km2)**	**% Area**
croplands	440.6	0.2	-425.6	-0.2	4742.6	1.8	3328.5	1.3	3328.5	1.3	3241.9	1.6
dense forest	-21045.3	-8.0	-15532.3	-5.8	-6536.5	-2.9	-9498	-2.7	-9498	-2.7	-9498	6.6
grassland/savannas	191.1	0.1	-54.9	0.0	818.9	0.3	138.6	0.1	138.6	0.1	-1.1	0.1
open savannas/barelands	5479.8	2.1	-1952.9	-0.7	-10277.0	-3.9	-2685.9	-1.0	-2685.9	-1.0	-2685.9	-0.6
built-up areas	628.3	0.2	2048.7	0.8	756.9	0.3	2605.9	1.0	2605.9	1.0	2340.3	1.2
water bodies	1795.4	0.7	-550.3	-0.2	844.9	0.3	-72.0	0.0	-72.0	0.0	-36.2	0.3
wetlands	12.3	0.0	2084.1	0.8	235.0	0.1	-291.7	-0.1	-291.7	-0.1	-291.7	0.0
woody savannas	12497.8	4.7	14080.1	5.3	10909.2	4.1	5151.7	2.1	5151.7	2.1	-24455.1	-8.4
