# Supplementary material for: Monitoring forest cover and land use change in the Congo Basin under IPCC climate change scenarios
Source: PLoS One. 2024 Dec 2;19(12):e0311816. doi: 10.1371/journal.pone.0311816 (PMC11611213; doi:10.1371/journal.pone.0311816)
Supplement: S18 Table — b; Quantified decadal changes in land cover patterns in Cameroon, between 1990–2020. (PDF) [file pone.0311816.s029.pdf]

S18a Table

	**1990**		**2000**		**2010**		**2020**		**2050**					
									**SSP1-2.6**		**SSP2-4.5**		**SSP5-8.5**	
**LULC class**	**Area (km2)**	**% Area**	**Area (km2)**	**% Area**	**Area (km2)**	**% Area**	**Area (km2)**	**% Area**	Area (km2)	% Area	Area (km2)	% Area	Area (km2)	% Area
croplands	33.6	0	11846	2.5	16176.8	3.5	22795.3	4.9	40599.4	8.7	40504.6	8.7	44072.2	9.4
dense forest	236645.9	50.7	218279.3	46.8	203409.5	43.6	195146.1	41.8	193203.9	41.4	193209.6	41.4	183804.1	39.4
grassland/savannas	1775.1	0.4	2453.6	0.5	3293.6	0.7	3693.3	0.8	3108.6	0.7	3107.9	0.7	3339.8	0.7
open savannas/barelands	179040.7	38.4	163023.1	34.9	172200.1	36.9	167130.1	35.8	144000.1	30.9	143994.7	30.9	141579	30.3
built-up areas	876.7	0.2	8217.6	1.8	8932.3	1.9	14604.3	3.1	28999.7	6.2	29111.2	6.2	30934.3	6.6
water bodies	3100.2	0.7	4024.5	0.9	3595.9	0.8	3541.2	0.8	3352.5	0.7	3352.5	0.7	3362.3	0.7
wetlands	700	0.1	527.7	0.1	3431.1	0.7	3751.5	0.8	3564.9	0.8	3564.9	0.8	3534.2	0.8
woody savannas	44603.6	9.6	58403.9	12.5	55883.6	12	56268.7	12.1	49715.6	10.7	49699.2	10.7	55918.7	12
Total	466775.7	100	466775.7	100	466923	100	466930.4	100	466544.6	100	466544.6	100	466544.6	100

S18b Table

	**1990-2000**		**2000-2010**		**2010-2020**		**2020-2050**					
							**SSP1-2.6**		**SSP2-4.5**		**SSP5-8.5**	
**LULC classes**	**Area (km2)**	**% Area**	**Area (km2)**	**% Area**	**Area (km2)**	**% Area**	**Area (km2)**	**% Area**	**Area (km2)**	**% Area**	**Area (km2)**	**% Area**
croplands	11812.4	2.5	4330.8	0.9	6618.5	1.4	17804.1	3.8	17709.3	3.8	21276.9	4.5
dense forest	-18366.5	-3.9	-14869.8	-3.2	-8263.4	-1.8	-1942.2	-0.4	-1936.5	-0.4	-11342	-2.4
grassland/savannas	678.6	0.1	840.0	0.2	399.7	0.1	-584.7	-0.1	-585.4	-0.1	-353.5	-0.1
open savannas/barelands	-16017.6	-3.4	9177.1	2.0	-5070.1	-1.1	-23130	-4.9	-23135.4	-4.9	-25551.1	-5.5
built-up areas	7340.9	1.6	714.8	0.2	5671.9	1.2	14395.4	3.1	14506.9	3.1	16330	3.5
water bodies	924.3	0.2	-428.6	-0.1	-54.7	0.0	-188.7	-0.1	-188.7	-0.1	-178.9	-0.1
wetlands	-172.3	0	2903.5	0.6	320.3	0.1	-186.6	0	-186.6	0	-217.3	0
woody savannas	13800.2	3	-2520.3	-0.5	385.1	0.1	-6553.1	-1.4	-6569.5	-1.4	-350	-0.1
