# Supplementary material for: Monitoring forest cover and land use change in the Congo Basin under IPCC climate change scenarios
Source: PLoS One. 2024 Dec 2;19(12):e0311816. doi: 10.1371/journal.pone.0311816 (PMC11611213; doi:10.1371/journal.pone.0311816)
Supplement: S19 Table — Results are shown for the most important LULC variables that can help support policy planning. (PDF) [file pone.0311816.s030.pdf]

S19 Table

LULCC categories	1990 - 2000	2000 - 2010	2010 - 2020	2020 - 2050 - SSP1-2.6	2020 - 2050 - SSP2-4.5	2020 - 2050 - SSP5-8.5
CR	1716.5	20790.1	22019.2	80081.6	79671.7	79567.5
DF -> CR	4993.9	1011.9	5608.6	19233.1	21425.7	23290.3
GR -> CR	190.9	760.5	1582.3	2509.4	2308.5	2151.2
OP -> CR	22595.6	10083.2	46550.8	60311.7	55841.8	61757
BU -> CR	13.7	140.6	935.5	2889.6	2860.4	2702.6
WS -> CR	3276.6	2473.4	8935.2	16099.7	14463.5	15801.2
CR -> DF	7.9	1759.9	742.2	0	0	1657
DF (unchanged)	1986951	1853177	1769114	1719433.2	1701767.4	1688956.3
GR -> DF	3017.8	10868.7	6792	13762.0	239.0	176.6
OP -> DF	74175.9	79180.8	70060.2	0	0	0
BU -> DF	3.4	118.5	1247.8	0	0	0
WS -> DF	59118.5	121462.4	129135.2	65746.2	65745.5	96643.8
CR -> GR	9.1	291.6	571.9	0	0.0	0
DF -> GR	19513	8561.1	11373.1	10075.3	9048.1	9145.6
CR -> OP	56.9	7773.5	8742.4	0	0	0
DF -> OP	132092	82981.1	77987	0	0	0
BU -> OP	335.4	960.9	2803.5	0	0	362.8
CR -> BU	77940.2	405.7	1685.1	5852.1	6262.1	6003.6
DF -> BU	3212.9	430.4	2708.4	7360.2	13029.2	12961.9
GR -> BU	176.2	93.2	404.5	916	709.9	2408.3
OP -> BU	13183.3	7159.9	17997.5	27896.8	23636	30897.2
BU (unchanged)	1294.9	18260.7	20275.1	43510.2	43539.5	41530.4
WS -> BU	1823.7	408.1	2976.6	6575.3	5508.6	8342.4
WB	48226.2	50590.7	29401.2	56715.5	56793.1	56782.4
WL	249.2	250.4	20363.1	11684.3	11717.7	11676.3
CR -> WS	9.7	1697.3	1160.8	0	0	0
DF -> WS	182873.5	168286.1	196065.5	212724.5	177286.4	149925.2
WS (unchanged)	202590.8	228902.4	262859.6	531645.6	555319.2	541228.5
Other LULCC	1275611	1434689	1386582	1192730.7	1214506.7	1209087.1

* CR = Croplands; DF = Dense forest; GR = Grassland savannas; OP = Open savannas/barelands; WB = Water bodies; WL = Wetlands; WS = Woody savannas; BU = Built-up areas
